# High‐Spin Iron(VI), Low‐Spin Ruthenium(VI), and Magnetically Bistable Osmium(VI) in Molecular Group 8 Nitrido Trifluorides NMF_3_

**DOI:** 10.1002/chem.202101404

**Published:** 2021-06-26

**Authors:** Tony Stüker, Xiya Xia, Helmut Beckers, Sebastian Riedel

**Affiliations:** ^1^ Institut für Chemie und Biochemie Anorganische Chemie Freie Universität Berlin Fabeckstr. 34/36 14195 Berlin Germany

**Keywords:** ab initio calculations, high oxidation states, matrix isolation, N ligands, transition metals

## Abstract

Pseudo‐tetrahedral nitrido trifluorides N≡MF_3_ (M=Fe, Ru, Os) and square pyramidal nitrido tetrafluorides N≡MF_4_ (M=Ru, Os) were formed by free‐metal‐atom reactions with NF_3_ and subsequently isolated in solid neon at 5 K. Their IR spectra were recorded and analyzed aided by quantum‐chemical calculations. For a d^2^ electron configuration of the N≡MF_3_ compounds in *C*_3v_ symmetry, Hund's rule predict a high‐spin ^3^A_2_ ground state with two parallel spin electrons and two degenerate metal d(δ)‐orbitals. The corresponding high‐spin ^3^A_2_ ground state was, however, only found for N≡FeF_3_, the first experimentally verified neutral nitrido Fe^VI^ species. The valence‐isoelectronic N≡RuF_3_ and N≡OsF_3_ adopt different angular distorted singlet structures. For N≡RuF_3_, the triplet ^3^A_2_ state is only 5 kJ mol^−1^ higher in energy than the singlet ^1^A′ ground state, and the magnetically bistable molecular N≡OsF_3_ with two distorted near degenerate ^1^A′ and ^3^A“ electronic states were experimentally detected at 5 K in solid neon.

## Graphical abstract

**In different states**: Molecular hexavalent group 8 nitrido fluorides feature different electronic ground‐state d^2^ configurations in solid Ne matrices. While NFeF_3_ adopts a high symmetry structure, the heavier homologues are subject to symmetry lowering and spin‐crossover caused by a pseudo Jahn−Teller effect “hidden” in the excited states. For NOsF_3_ two coexisting high‐ and low‐spin structures show triplet–singlet spin crossover and magnetic bistability. A weakened covalent N−Fe bond in NFe^VI^F_3_ indicates the non‐innocent character of the nitrido ligand.

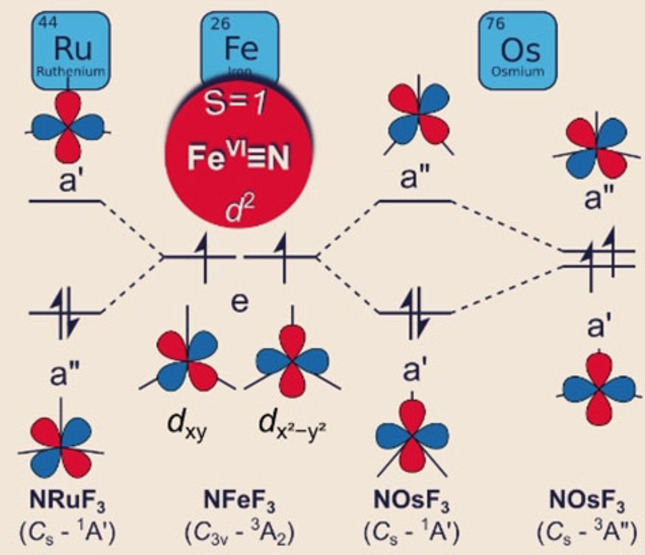

## Introduction

The group 8 transition metals have eight electrons in their valence shell, but in addition to the well‐known strong oxidizers RuO_4_ and OsO_4_, only Os has a variety of different complexes in oxidation state VIII.^[1]^ While the oxidation state VI is abundant for ruthenium and osmium, the complex anion [FeO_4_]^2−^ was the only known Fe^VI^ compound for a long time.^[2]^ In 2007 the neutral, dioxo Fe^VI^ peroxide O_2_Fe(η^2^‐O_2_) was reported to be formed from molecular FeO_2_ and O_2_ under cryogenic conditions.^[3]^ Tetrahedral Fe^VIII^O_4_ was shown to be metastable with respect to O_2_Fe^VI^(η^2^‐O_2_) in the gas phase,^[4]^ and the oxidation state VII is so far the highest oxidation state of iron observed experimentally for the tetrahedral tetroxide anion FeO_4_^−^.^[5]^ In addition to oxygen, nitrogen ligands are also able to stabilize high oxidation states of iron. Such terminal iron‐nitrido complexes have already been the subject of several up‐to‐date reviews.^[6]^ We restrict ourselves to some representative examples such as the square‐pyramidal [(TPP)Fe^V^N] (TPP^2−^=tetraphenylporphyrinate dianion), characterized by Raman spectroscopy,^[7]^ the tetragonal nitrido Fe^VI^ dication [(Me_3_cyac)FeN]^2+^ ([Me_3_cyac]^−^=*N,N,N*‐tri‐methyl‐1,4,8,11‐tetraazacyclotetra‐decane‐1‐acetate), confirmed by Mössbauer and X‐ray spectroscopy,^[8]^ the pseudo‐tetrahedral [(PhB(PCH_2_P*i*Pr_2_)_3_)Fe^IV^N] (PhB(PCH_2_P*i*Pr_2_)_3_=tris(diisopropylphosphinophenyl)borane),^[9]^ and, very recently, the crystal structure of a thermally stable four‐coordinate Fe^VI^ bis(imido) cation, [(H_2_B(MesIm)_2_)Fe(=NMes)_2_]^+^ ([H_2_B[MesIm]_2_]^−^=dihydrobis‐[1‐(2,4,6‐trimethylphenyl) imidazol‐2‐ylidene]borato).^[10]^

Nitrido iron complexes play an important role in a number of chemical and biological processes, for example in the catalytic cycle of cytochrome P450,^[11]^ in the FeMo cofactor of the nitrogenase enzyme^[6a]^ and in the Haber–Bosch process.^[12]^ In analogy to the active iron surface nitride in the Haber–Bosch process, ammonia synthesis has also successfully achieved under mild conditions using the ruthenium pincer nitrido complex [(PNP)RuN] (PNP^−^=[N(CH_2_CH_2_P^t^Bu_2_)_2_)]^−^).^[13]^ Quite recently osmium(VI) nitrides have emerged as a new class of potential anticancer and antitumor agents.^[14]^ Examples include [(bipy)Cl_3_Os^VI^N]^[15]^ (bipy=2,2’‐bipyridine)^[16]^ and [(sap)(py)ClOs^VI^N] (sap=deprotonated *N*‐salicylidene‐2‐aminophenol).^[17]^ The wide field of possible applications of group 8 nitrido complexes underline the importance of a deeper understanding of the properties of this class of compounds. Especially the nitrido metal–ligand multiple bond and the valency of the metal are key factors for the reactivity and structure of these compounds.

**Scheme 1:**
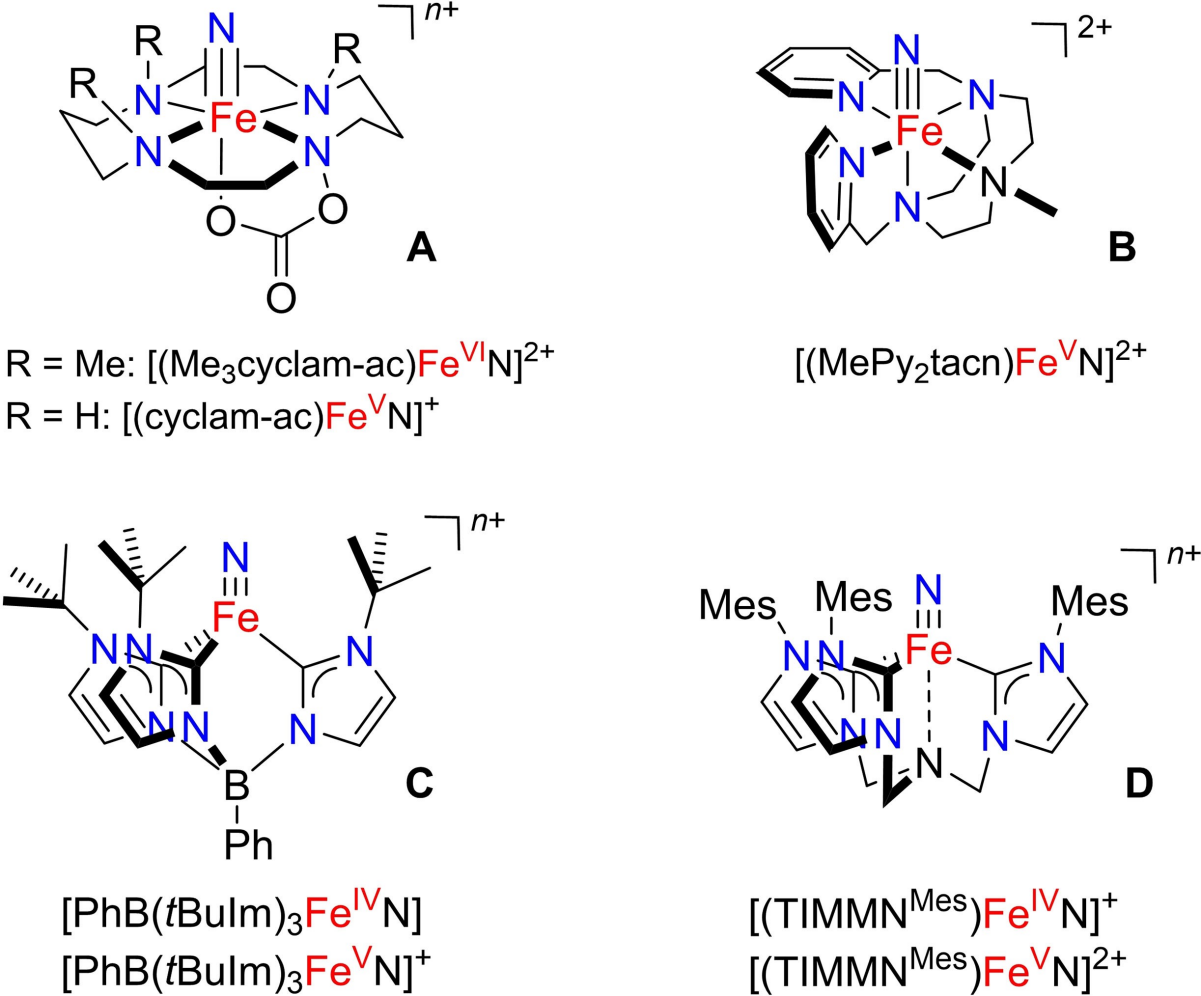
Representative examples of ligand‐supported tetragonal (**A**[^8^, ^22^] and **B** ^[19d]^) and trigonal (**C** ^[19a]^ and **D** ^[19b]^) coordinated high‐valent iron nitrido complexes.

In particular, there has been a tremendous progress in the synthesis and the chemistry of molecular Fe^IV^ and Fe^V^ nitrido compounds in the recent years that have been described in detail in several review articles.^[6]^ They are supported by sterically encumbered macrocyclic or chelating ligands involving nitrogen or *N*‐heterocyclic carbene donors based on, for example porphyrin or nitrogen‐ and boron‐anchored tri‐ and tetrapodal chelates to protect the reactive Fe≡N moiety (see Scheme 1 for representative examples). The most common route to these nitrido compounds is the photolysis of an iron azido precursor and concomitant N_2_ evolution, whereby the one‐electron oxidation of the Fe^IV^ nitrido complexes often represents an alternative route to Fe^V^ nitrido complexes.[^6a^, ^6b^]

The reactivity of these high‐valent nitrido iron compounds in chemical transformations have been thoroughly explored,[^6^, ^18^] their structures, and their electronic properties have been investigated in detail using a variety of experimental and quantum mechanical methods.^[19]^ While these studies contributed greatly to the understanding of the iron nitride bonding motif, our knowledge about the behavior, the nature, and bond‐strengths of the Fe≡N triple bond in high valent iron compounds upon iron oxidization is, however, still very limited and contradictory. Two questions arise here: *Is there a nitrido wall* ^[20]^ *from which the nitrido ligand gives up its innocent behavior*,^[21]^ *and does the Fe≡N bond become stronger and stronger through oxidation of the iron center?*

It should be emphasized that the known iron nitrido species can be divided into trigonal (pseudo‐tetrahedral) and tetragonal (pseudo‐octahedral) complexes (Scheme 1), since these two groups show different ligand field splitting of the Fe(3d) orbitals.[^9^, ^23^] In a trigonal *C*_3v_ ligand field there are two purely Fe≡N nonbonding *e*‐type orbitals (d_*xy*,*x*2−*y*2_), which allow the accommodation of up to four electrons energetically below the antibonding Fe≡N orbitals.[^6a^, ^6c^, ^9^, ^23^] This results in a relatively strong Fe≡N triple bonds, for example, low spin Fe^IV^ derivatives, for which very short experimental Fe−N distances (Table S1 in the Supporting Information) and Fe−N stretching vibrations at 1008–1034 cm^−1^ were found.[^9^, ^24^]

Conversely, in the tetragonal *C*_4v_ ligand field there is only one purely nonbonding (d_*xy*_) orbital with respect to the Fe≡N bond energetically below the π*‐antibonding (d_*xz*,*yz*_) MOs.[^6a^, ^6c^] A d‐electron count larger than two results here in the occupation of π*(Fe≡N) orbitals, and, accordingly, Fe^IV^ (d^4^) and Fe^V^ (d^3^) nitrido complexes in tetragonal symmetry are generally thermally less stable and more reactive.[^6b^, ^18a^, ^18c^] Note that the d^3^ ground‐state electron configuration of Fe^V^ nitrido complexes is subject to a Jahn−Teller distortion.[^19b^, ^19c^] To overcome the thermal instability and high reactivity of such tetragonal Fe^V^ nitride complexes their Fe≡N distances and stretching frequencies were obtained by a variety of spectroscopic methods either at cryogenic temperatures or at the gas phase (for representative examples, see Table S1). As expected, the experimental Fe−N distances for the two tetragonal complexes [Fe^V^(N)(MePy_2_tacn)]^2+^ (Scheme 1, 3d^4^ configuration, Fe−N: 164(1) pm)^[19d]^ and [Fe^V^(N) (cyclam‐ac)]^+^ (Scheme 1, cyclam‐ac=1,4,8,11‐tetraazacyclotetradecane‐1‐acetato, Fe−N: 161(1) pm),^[22]^ estimated from extended X‐ray absorption fine structure (EXAFS) analysis, were found to be longer than the Fe−N distance of the analogous Fe^VI^ dication [Fe^VI^(N)(Me_3_cyclam‐ac)]^2+^ (Scheme 1, 157(2) pm) with a singlet 3d^2^ configuration.^[8]^

In contrast, the formal Fe≡N bond order in trigonal Fe‐nitrido complexes does not change by increasing the iron oxidation state from singlet Fe^IV^ to triplet Fe^VI^, making predictions about the bond lengths less intuitive as other factors such as the geometry and the nature of the ligands come to the fore. X‐ray structure analysis of the Fe^IV^N/Fe^V^N derivatives of the two redox pairs [PhB(*t*BuIm)_3_FeN]^0/+^ (Scheme 1)[^19a^, ^24b^] and [(TIMMN^MES^)FeN]^+/2+^ (Scheme 1)^[19b]^ show different trends. While the Fe−N length decreases slightly from 151.2(1) pm to 150.6(2) pm for the former, it increases from 151.3(3) pm to 152.9(1) pm for the latter. The different trend in these Fe≡N distances during oxidation of Fe^IV^ to Fe^V^ was attributed to a possibly stronger interaction between the ligand N anchor with the more electrophilic Fe^V^ center in [Fe(N)(TIMMN^MES^)]^2+^ (Scheme 1).^[19b]^ On the other hand, also coordinated solvent molecules can make it difficult to compare the Fe≡N distances of different complexes, since this leads to shortened experimental Fe≡N distances.^[19e]^

In this work, we describe the preparation of the molecular, neutral nitrido trifluorides NM^VI^F_3_ of the group 8 metals M=Fe, Ru, Os from IR laser ablated metal atoms and gaseous NF_3_ and their IR‐spectroscopic characterization under cryogenic conditions in a noble gas matrix. These trigonal nitrido trifluorides bear genuine M≡N triple bonds, unsupported by sterically encumbered electron donor substituents with the innocent fluoride ligand. The M≡N stretching vibration of theses derivatives is energetically sufficiently isolated from other fundamentals. Hence, it is considered to be a reliable experimental signature for M−N bond strength and M−N bond length in these nitrido complexes. This analysis overcomes the difficulties described above and also has the advantage that the experimental results can be supported and analyzed by reliable and accurate quantum mechanical calculations of these molecular, neutral compounds. Furthermore, this analysis enables a direct comparison of experimental M≡N stretching frequencies of M=Fe^VI^ and its heavier group 8 congeners with those of the analogous nitrido trifluorides N≡MF_3_ of group 6 (M=Cr, Mo, W)^[25]^ and group 9 (Co, Rh, Ir)^[26]^ transition metals which have been studied previously. To the best of our knowledge, N≡Fe^VI^F_3_ is the first experimentally verified neutral, nitrido iron(VI) complex. In addition, we have evidence for the formation of NM^VII^F_4_ (M=Ru, Os).

For an electronic metal d^2^ configuration of these N≡MF_3_ compounds in *C*_3v_ symmetry Hund's rule predict that two parallel spin electrons occupy the degenerate M(d_*xy*,*x*2−*y*2_) orbitals of *e*‐type symmetry resulting in a non‐degenerate high‐spin ^3^A_2_ ground state. Although this ^3^A_2_ state is not Jahn−Teller (JT) active, an electronic e^2^ configuration can generally lead to a Jahn−Teller distorted ground state as a result of a strong pseudo‐Jahn−Teller (PJT) mixing of two excited singlet electronic states.^[27]^ This is because an electronic e^2^ configuration in *C*_3v_ symmetry, in addition to the ^3^A_2_ state, is generally associated with two electronic singlet states ^1^A_1_ and ^1^E. These electronic states are reminiscent of the well‐known singlet excited states of molecular oxygen.^[28]^

It has been noted that the JT stabilization energy of the excited ^1^E state is usually much weaker than the PJT stabilization resulting from mixing of the two excited ^1^A_1_ and ^1^E states. The stabilization energy of this PJT interaction can be so large that the lower of these excited states crosses the ^3^A_2_ potential energy surfaces and become the distorted global minimum configuration.[^27^, ^31^] We observed such a “hidden” PJT distortion for N≡RuF_3_ and N≡OsF_3_ but not for NFe≡F_3_. Note that this distortion is also associated with a PJT‐induced triplet‐singlet spin crossover.^[27a]^

## Results

### Vibrational wavenumbers of group 8 nitrido trifluorides NM^VI^F_3_ and tetrafluorides NM^VII^F_4_

**Figure 1:**
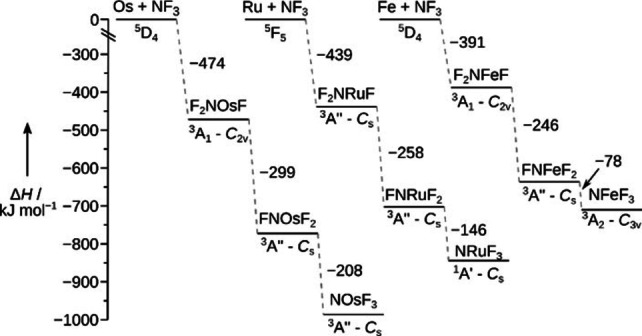
Stationary points on the reaction coordinate obtained at the BP86 level of theory for the formation of the nitrido metal complexes N≡MF_3_ starting from the free metal atoms M and NF_3_ (*C*_3v_ – ^1^A_1_). See Table S2 for more details.

The IR spectra of the novel group 8 metal nitrido trifluorides, N≡MF_3_ (M=Fe, Ru, Os) were recorded from the products obtained from laser‐ablated free metal atoms with NF_3_ seeded in a 1 : 1000 excess of neon after their deposition at 5 K on a gold‐plated copper mirror (for experimental details see the Supporting Information). According to density functional theory calculations, the direct insertion of the metal atoms into an F−N bond of NF_3_ to yield F_2_N−MF, and the subsequent fluorine migration from nitrogen to the metal center to FN=MF_2_ is highly exothermic for all three metals (Figure 1, Table S2).

**Figure 2:**
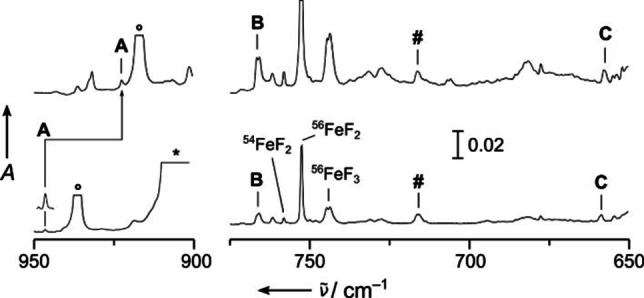
IR absorption spectra obtained from co‐deposition of laser‐ablated iron with 0.1 % ^14^NF_3_ (bottom) and 0.1 % ^15^NF_3_ (top) in solid Ne. Bands labeled with **A**, **B** and **C** are assigned to NFeF_3_ (Table 1). Band **A** is enhanced by a factor of five. Known bands of binary iron fluorides^[29]^ are labeled, and an unassigned band showing no ^14/15^N isotopic shift is labeled with a hash mark. The bands associated with NF_2_ and NF_3_ are marked with circles and asterisks, respectively.^[30]^ For more details, see Figure S1.

**Figure 3:**
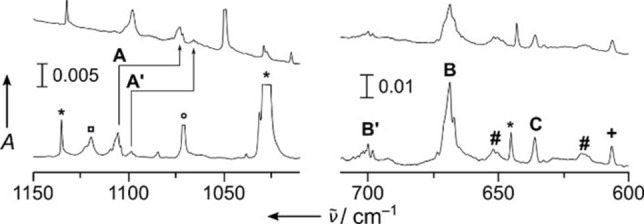
IR absorption spectra obtained from co‐deposition of laser ablated ruthenium with 0.1 % ^14^NF_3_ (bottom), and ^15^NF_3_ (top) in solid Ne, respectively. Bands labeled **A**–**C** are attributed to NRuF_3_ and **A’** and **B’** are due to NRuF_4_. Unknown bands are labeled by a pound and a plus sign, respectively. The bands associated with ^14^NF, ^14^NF_2_ and ^14^NF_3_ are marked with squares, circles, and asterisks, respectively.^[30]^ For more details, see Figure S2.

**Figure 4:**
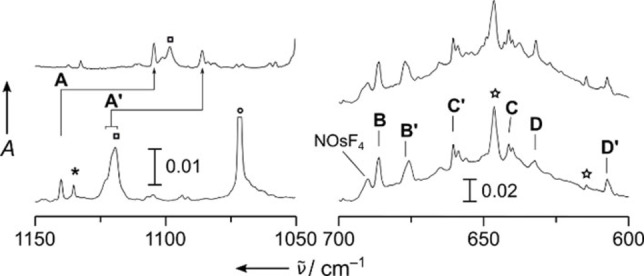
IR absorption spectra of laser ablated osmium co‐deposited with 0.1 % ^14^NF_3_ in solid Ne (bottom), with 0.1 % ^15^NF_3_ in Ne (top). Bands labeled **A**–**D** are attributed to NOsF_3_ (^1^A’) and **A’**–**D’** to NOsF_3_ (^3^A”). The bands marked with a pentagram sign are binary osmium fluorides. The bands associated with NF, NF_2_ and NF_3_ are marked with squares, circles and asterisks, respectively.^[30]^ For more details see Figure S3.

The rearrangement of the fluorimido complexes to the hexavalent nitrido trifluorides N≡MF_3_ is found to be considerably exothermic for osmium (−208 kJ mol^−1^), ruthenium (−146 kJ mol^−1^), and iron (−78 kJ mol^−1^) at the BP86/def2‐QZVP^[32]^ level of theory (details see the Supporting Information). Experimental IR spectra are shown from the deposits obtained in solid neon for the iron (Figures 2 and S1), ruthenium (Figures 3 and S2), and the osmium experiments (Figures 4 and S3), respectively. Experimental band positions are compared with predicted ones from quantum‐chemical calculations in Tables 1 and S4 (for a detailed band assignment refer to the Supporting Information). The formation of molecular NFeF_3_ (*C*_3v_) is clearly proved by the assignment of all its stretching vibrations marked **A** (*ν*(NFe): 946.4 cm^−1^), **B** (*ν*_as_(FeF_3_): 766.8 cm^−1^), and **C** (*ν*_s_(FeF_3_): 658.8 cm^−1^) in Figure 2 (Table 1). Bands at 743.6/744.7, 752.6 and 785.1 cm^−1^ were assigned to the known molecular binary iron fluorides ^56^FeF_3_, ^56^FeF_2_ and ^54^FeF_2_, respectively.^[29]^ Their high intensity and the high yield of these binary fluorides compared to the NFeF_3_ product bands indicate the lower stability of NFeF_3_ under the harsh conditions of the laser ablation process. The spectra recorded in the ruthenium experiment (Figure 3), clearly revealed the presence of two different nitrido ruthenium complexes, finally assigned to NRuF_3_ (*C*_s_) and NRuF_4_ (*C*_4v_). The characteristic Ru≡N stretching bands of NRuF_3_ (*C*_s_) and NRuF_4_ (*C*_4v_) are labeled **A** (1105.4 cm^−1^, NRuF_3_) and **A’** (1098.5 cm^−1^, NRuF_4_) in Figure 3. The RuF_3_ stretching modes of *C*_s_ symmetric NRuF_3_ split into three modes. The strong antisymmetric F−Ru−F appears at 668.5 cm^−1^ (labeled **B** in Figure 3) and likely overlaps with the nearby weaker F’−Ru band. The symmetric F−Ru−F mode is attributed to the band labeled **C** in Figure 3 at 635.8 cm^−1^ (Table 1).

**Table 1:** Calculated and experimental vibrational wavenumbers (*ν*(^14^N) in cm^−1^) and ^14/15^N isotopic shifts (Δ*ν* in parentheses) for NFeF_3_, NRuF_3_, NRuF_4_, NOsF_3_ and NOsF_4_.

Exp.^[a]^	CCSD (T)^[b]^	Assignment
**NFeF_3_ (*C***_**3v**_, ^**3**^**A_2_)^[c]^**		
946.4 (−23.7)^[d]^	1028 (−26)^[e,f]^	NFe str., a_1_
766.8/766.7 (0)	737 (0)^[f]^	FeF_3_ str., e
658.8 (−1.1)	689 (−2)^[f]^	FeF_3_ str., a_1_
**N^102^RuF_3_ (*C***_**s**_, ^**1**^**A’)^[g]^**		
1105.4 (−32.7)	1085 (−32)	NRu str., a’
–^[h]^	682 (0)	F’‐Ru str., a’
668.5 (0)	678 (0)	antisym. F−Ru‐F str., a”
635.8 (0)	649 (0)	sym. F−Ru‐F str., a’
**N^102^RuF_4_ (*C***_**4v**_, ^**2**^**B_1_)^[g]^**		
1098.5 (−32.5)	1080 (−32)	NRu str., a_1_
700.1 (0)	711 (0)	RuF_4_ stretch, e
–^[h]^	681 (0)	RuF_4_ stretch, a_1_
–^[j]^	598 (0)	RuF_4_ stretch, b_2_
**NOsF_3_ (*C***_**s**_, ^**1**^**A’)**		
1140.1 (−35.5)	1152 (−36)	NOs str., a’
686.0/686.6 (0)	689 (0)	OsF_2_ sym. str., a’
641.3/640.0 (0)	664 (0)	OsF’ sym. str., a’
632.3 (0)	652 (0)	OsF_2_ antisym. str., a”
**NOsF_3_ (*C*s,** ^**3**^ **A”)**		
1086.0 (−) ^[i]^	1095 (−36) ^[i]^	^15^NOs str, a’
675.8/677.0	675 (0)	OsF_2_ antisym. str., a”
660.5/658.9 (0)	668 (0)	OsF_2_ sym. str., a’
607.4 (0.0)	614 (0)	OsF’ sym. str., a’
**NOsF_4_ (*C***_**4v**_, ^**2**^**B_1_)**		
–^[k]^	1145 (−36)	NOs str., a_1_
–^[k]^	706 (0)	OsF_4_ stretch, a_1_
689.9 (0)	693 (0)	OsF_4_ stretch, e
–^[j]^	635 (0)	OsF_4_ stretch, b_2_

[a] Neon matrix; matrix sites are separated by a slash. [b] Intensities from DFT calculations available in Table S4. [c] M06‐L/def2‐QZVP: 785 a_1_ (−11) [12], 703 e (0) [200], 617 a_1_ (−1) [40]. [d] ^14/15^N isotopic ratio: 1.0256. [e] ^14/15^N isotopic ratio: 1.0257. [f] NEVPT2/aug‐cc‐pwCVTZ‐DK. [g] For the experimentally observed Ru isotope splitting see Tables S5–S7 and Figures S4 and S5). [h] Band is likely hidden by the stronger antisymmetric F−Ru‐F stretching mode (a’’). [i] *ν*(^15^N−Os) in cm^−1^, see text. [j] Not IR active. [k] Too weak or overlapped.

For NRuF_4_ only the strongest RuF_4_ stretching band, the degenerate *e*‐type mode could safely be assigned to the band labeled **B’** in Figure 3 centered at 700.0 cm^−1^.

In the spectra obtained from the reaction of osmium atoms with isotopic labeled ^15^NF_3_ two Os≡N stretching bands appeared at 1104.6 and 1086 cm^−1^, which are labeled **A** and **A’**, respectively, in Figure 4, and which are finally assigned to different “spin‐isomers” of NOsF_3_ in near‐degenerate singlet ^1^A’ and triplet ^3^A” electronic states (Table 1). In the ^14^NF_3_ experiment **A** is observed at 1140 cm^−1^ (Figure 4), while **A’** is overlapped by a stronger band due to the ^14^NF radical at 1120.8 cm^−1^.^[30b]^ All three Os−F stretching bands of singlet NOsF_3_ (^1^A’) are assigned (Table 1) and labeled **B** (*ν*_s_(OsF_2_): 686.0 cm^−1^), **C** (*ν*(OsF′): 641.3 cm^−1^), and **D** (*ν*_as_(OsF_2_): 632.3 cm^−1^) in Figure 4, respectively. Bands labeled **B’**, **C’** and **D’** at 675.8 cm^−1^, 660.5 cm^−1^ and 607.4 cm^−1^, respectively, are assigned to the three Os−F stretching modes of triplet NOsF_3_ (^3^A^”^, Table 1). Finally, a band at 689.6 cm^−1^ in Figure 4 is tentatively assigned to the strongest vibrational mode of NOsF_4_ (*C*_4v_, Table 1). The tetrafluorides N≡MF_4_ (M=Ru, Os) are likely formed by the exothermic addition of a fluorine atom to N≡MF_3_ (Table S2).

### Pseudo‐Jahn−Teller distortion of molecular group 8 nitrido fluorides NM^VI^F_3_

The group 8 nitrido fluorides NM^VI^F_3_ adopt metal *d* ^2^ configurations, for which Hund's rule predicts a high‐spin ^3^A_2_ ground state in an undistorted *C*_3v_ symmetry and two parallel spin electrons in the twofold degenerate *e*(d_*xy*,*x*2‐*y*2_)‐orbital (|*e*_ϵ_↑;*e*_θ_↑⟩), labeled 9e for NFeF_3_ in the Supporting Information Figure S6. Three *e* ^2^ terms (four states) can be formed, ^3^A_2_ (|*e*_ϵ_↑;*e*_θ_↑⟩), ^1^A_1_ (√1/2 [|*e*_ϵ_↑;*e*_ϵ_↓⟩+|*e*_θ_↑;*e*_θ_↓⟩]), ^1^E_θ_ (√1/2 [|*e*_ϵ_↑;*e*_ϵ_↓⟩–|*e*_θ_↑;*e*_θ_↓⟩]) and ^1^E_ϵ_ (√1/2 [|*e*_θ_↑;*e*_ϵ_↓⟩+|*e*_θ_↓;*e*_ϵ_↑⟩]). Due to the nondegenerate nature and totally symmetric charge distribution of the ^3^A_2_ state no Jahn−Teller distortion is expected.^[31]^ Other distributions of the electrons, as outlined above, result in configurations with lower spin and the absence of low‐lying triplet excited states rule out obvious ground state pseudo‐Jahn−Teller distortions.

**Figure 5:**
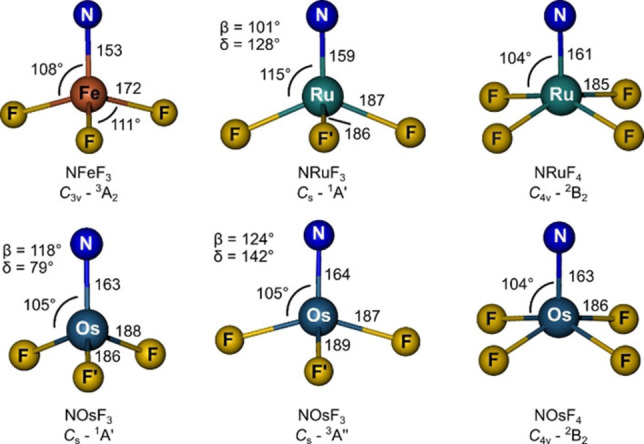
Ground‐state structures of NMF_3_ and NMF_4_ calculated at the CCSD(T)/aVTZ (M=Ru, Os, M: aVTZ‐PP) and the NEVPT2/aVTZ‐DK (NFeF_3_, Fe: awCVTZ‐DK) levels of theory. Bond lengths are given in pm and angles in degrees. β denotes the N–M–F’ and δ the F−M−F angle for structures with *C*_s_ symmetry.

Nevertheless, as shown in Figure 5 and in agreement with experimental vibrational assignments, all four NMF_3_ species possess surprisingly different structures and the *C*_3v_ symmetric ground state was only verified for NFeF_3_. In case of NRuF_3_, extensive CCSD(T)/CBS calculations (Table S10) find the high symmetric ^3^A_2_ is just about 5 kJ mol^−1^ higher than the distorted ^1^A’ ground state. According to our experimental data, NOsF_3_ features two quasi‐degenerate, distorted structures in ^1^A’ and ^3^A” electronic states, separated by only Δ*E*_T−S_=−1.3 kJ mol^−1^ (CCSD(T)/CBS, Table S11).

**Figure 6:**
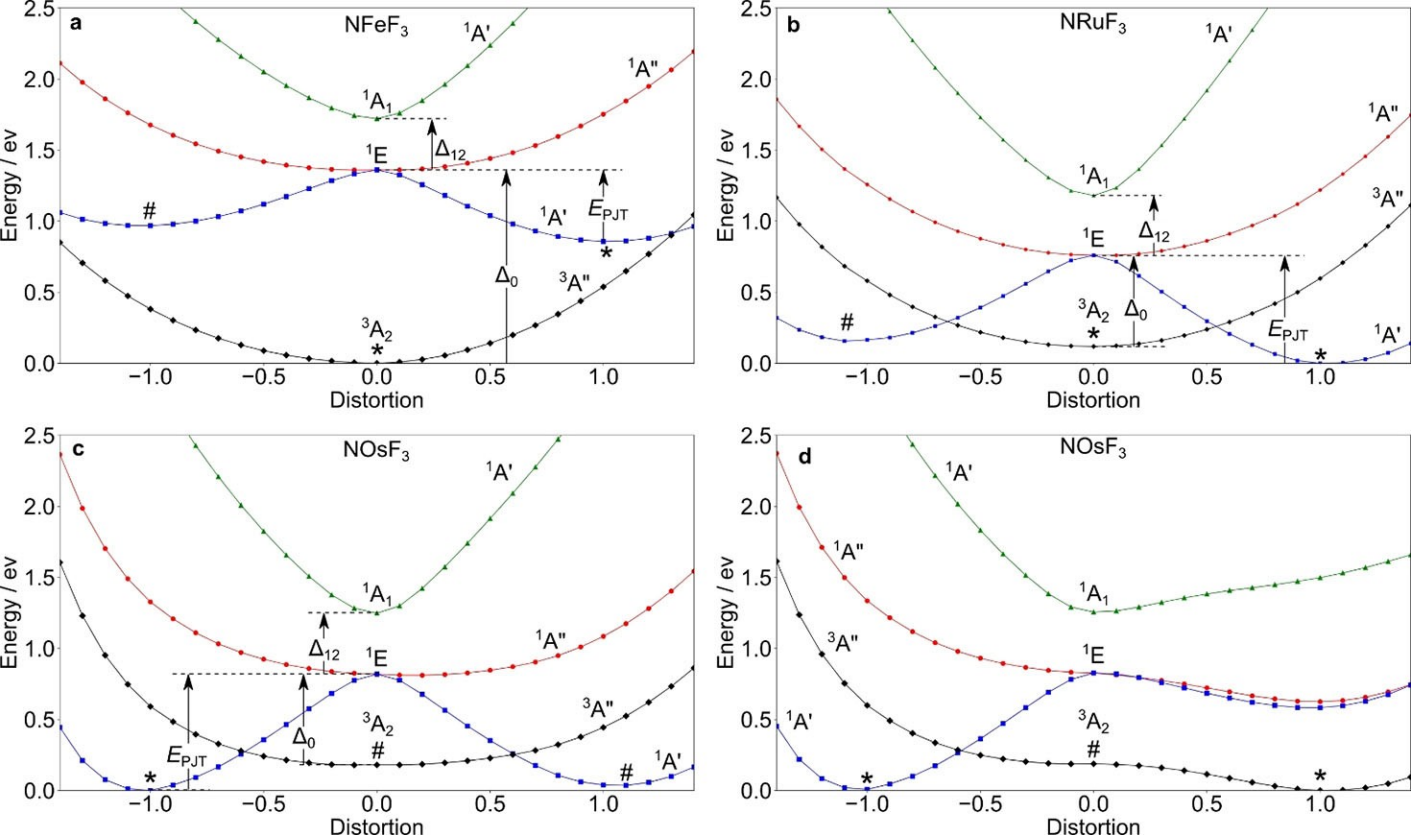
Cross section of the APES for the terms arising from the electronic *e* ^2^ configurations of a) NFeF_3_, b) NRuF_3_, c), d) NOsF_3_ along the distortion coordinate (*D*) connecting stationary points located at *D*=1, 0 and −1, respectively, on the ^1^A’ (blue line) and the ^3^A_2_ (^3^A”, black line) surfaces. Minimum points are marked with an asterisk, and first‐order saddle points with a hash mark. The PJT stabilization energy (*E*_PJT_) of the lowest excited state, its excitation energy at *C*_3v_ symmetry (Δ_0_), and the ^1^E–^1^A_1_ energy gap (Δ_12_) are indicated in (a)–(c).

To elucidate these findings, adiabatic potential energy surface (APES) scans were carried out using state‐averaged complete active space self‐consistent field calculations by distributing eight electrons in the eight molecular orbits formed by the metal (n‐1)*d* and N(2*p*) orbitals (SA‐CASSCF(8,8)) with subsequent NEVPT2 treatment to recover dynamic correlation. Shown in Figure 6a–c are cross sections along a distortion coordinate (*D*) that connects the two stationary points of the ^1^A’ surface, at *D*=−1 and 1, respectively, via the high‐symmetry *C*_3v_ stationary point at *D*=0.

The distortions take place along one component of the lowest (NFeF_3_, NRuF_3_) or imaginary (NOsF_3_) degenerate *e* normal mode in the high‐symmetry *C*_3v_ configuration. Therefore, mainly bond angle distortions are involved, in particular the dihedral angle F’−M−N−F (α, Figure S9), and the valence angles N−M−F’ (β, Figure 5), and N−M−F (γ). The sign of the distortion *D* in Figure 6 indicates a widening (positive) or closing (negative) of α. Differences in these angles and in the three nonequivalent bond distances between two localized stationary points in *C*_S_ symmetry were divided into equal incremental steps and used as intermediate internal coordinates in the APES calculation for each step (Tables S14–S17). In the case of Figure 6d the distortion in the positive direction was carried out using the NOsF_3_^3^A” minimum structure at *D*=1. The graphs shown in Figure 6a–d represent the energies of the terms arising from the electronic *e* ^2^ configuration, as outlined above. They demonstrate the propensity of trigonal group 8 nitrido complexes in the oxidation state VI to be subject to a PJT distortion. Other trigonal systems displaying a (A+E) ⊗ *e* Pseudo‐Jahn−Teller effect (PJTE) that is “hidden” in excited states (h‐PJTE) have already been described.[^27a^, ^31^] The condition for a distorted ground state minimum structure caused by the h‐PTJE is that the PJT stabilization energy of an excited state (*E*_PJT_) is larger than the energy gap Δ_0_ between the ground state in the high‐symmetry configuration and the PJT active excited state (*E*_PJT_>Δ_0_, see Figure 6, a–c).^[27a]^ The global minimum of the APES of NFeF_3_ shown in Figure 6a is located at the high‐symmetry point. The stationary points on the ^1^A’ (blue line) surface are a local minimum (*D*=1) and a first‐order saddle point (*D*=−1) without surface crossings in between. Consistent with the experimental vibrational data the global minimum is the high symmetry configuration. The h‐PJTE in the ^1^E state is not strong enough to distort the high‐symmetry configuration. The PJT stabilization energy, *E*_PJT_, is about 0.39 eV and smaller than the ^1^E‐^3^A_2_ energy gap Δ_0_=1.36 eV. The ^1^A’ minima, which features a (|*e*_θ_↑;*e*_θ_↓⟩) electronic configuration, and the ^3^A_2_ minima are separated by about 0.86 eV. The angular distortion from *D*=−1 to *D*=1 at the ^1^A′ surface extends from about 101°–132° (α), 100°–120° (β), and 112°–104° (γ).

The cross section of the APES of NRuF_3_ along the distortion coordinate from *D*=0 to *D*=1 illustrated in Figure 6 **b** shows that one of the components of the ^1^E term is stabilized by the strong PJT coupling with the excited ^1^A_1_ state. It crosses the ^3^A_2_ ground state of the undistorted high‐symmetry configuration to produce the global minimum with a distorted structure. The triplet‐singlet spin crossover is associated with an orbital disproportionation,^[27a]^ because in the distorted structure the electrons are paired in one *e*_θ_ orbital (|*e*_θ_↑;*e*_θ_↓⟩) instead of the symmetric distribution (|*e*_ϵ_↑;*e*_θ_↑⟩) in the undistorted configuration. Accordingly, we find that *E*_PJT_=0.76 eV is larger than Δ_0_=0.64 eV. The high‐spin ^3^A” state is higher in energy by only ∼0.12 eV and it has an energy barrier of ∼0.25 eV to the point of spin crossover with the low‐spin ^1^A’ state.

Figures 6c and d exhibit four relevant low‐lying stationary points on the ^1^A’ and ^3^A” APES of NOsF_3_. The h‐PJTE in this case produces a minimum with a distorted ^1^A’ structure at *D*=−1 and accordingly, the orbital disproportionation and spin crossover leads to a (|*e*_ϵ_↑; *e*_ϵ_↓⟩) configuration with *E*_PJT_=0.82 eV and Δ_0_=0.60 eV. Unlike the former two cases, the ^3^A_2_ high‐symmetry configuration of NOsF_3_ does not represent a minimum point, but a first order saddle point. Following the ϵ component of the imaginary *e* mode in Figure 6d we find – in accordance with the CCSD(T)/CBS results – an energetically quasi‐degenerate distorted ^3^A” minimum that shows orbital disproportionation, but no spin crossover about 0.1 eV (or 0.7 kJ mol^−1^) lower than the ^1^A’ state. The energy barrier of the spin crossover point is ∼0.27 eV (CCSD(T)/VTZ‐PP: 0.24 eV, Table S12), a significant barrier connecting both stationary points at the experimental cryogenic conditions. These findings support the observation of two different species in the experimental infrared spectra which correspond to species in different ^1^A’ and ^3^A” electronic states. We did not analyze the source of the distortion of the high‐spin minimum (^3^A”). But, under the premise that PJTE is the only source for symmetry breaking of non‐degenerate high‐symmetry states,[^27b^, ^31^] the source is most likely an interacting triplet ^3^E excited state.

## Discussion

All metal specific bands showing a ^14/15^N isotopic shift were successfully assigned. Bands due to binary fluorides are always present in experiments using IR laser ablation of metals in the presence of molecular fluorides as precursors. They are likely formed by recombination of metal atoms and atomic fluorine radicals formed by thermal or photolytic decomposition of the fluoride precursor in the hot plasma plume region or by the decomposition of metal fluoride product molecules. However, the very strong NF_3_ precursor bands and comparatively weak NF and NF_2_ bands in all spectra suggest that the formation of the NMF_3_ title product can be attributed to the reaction of M and NF_3_. Lower nitrido fluorides NMF or NMF_2_ could in principle also be formed through the cleavage of a metal‐fluorine bond or by the reaction of metal atoms with NF or NF_2_, but have so far not been identified.[^25^, ^26^, ^30c^, ^33^] The addition of fluorine to NMF and NMF_2_ to yield NMF_3_ and also the formation of NMF_4_ for M=Ru and Os are calculated to be exothermic (Table S2).

As shown here, all the trigonal NMF_3_ species possess two equilibrium configurations with different spin multiplicities, while those of NRuF_3_ and NOsF_3_ are close in energy. Such a magnetic and structural PJT induced bistability may also be possible for ligand‐stabilized trigonal nitrido d^2^ metal complexes. Such compounds are of interest for molecular switching, especially when symmetry breaking is involved (as for NFeF_3_ and NRuF_3_).^[34]^

**Figure 7:**
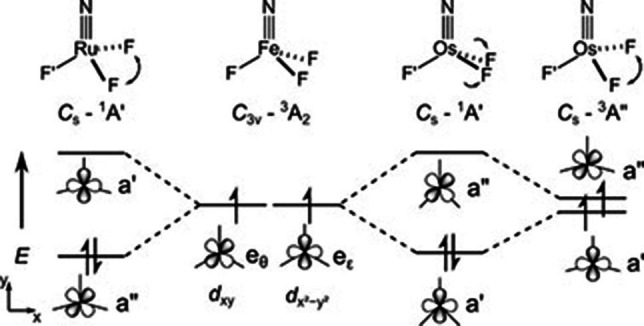
Comparison of the different d^2^ electron configurations of the NMF_3_ species (M=Fe, Ru, Os). The metal centered a’ and a”‐MOs are dominantly M(d_*x*2–*y*2_) and M(d_*xy*_) atomic orbitals, respectively.

The different stationary structures that were obtained for the group 8 NMF_3_ molecules shown in Figure 5 possess surprisingly different electronic configurations, as outlined above and summarized in Figure 7 (for molecular orbital plots, see Figures S6 and S8). The different ^1^A’ electronic ground states of NRuF_3_ and NOsF_3_ arise from the pairing of two unpaired electrons in different orbitals, which are associated with two different structural distortions. The HOMO of NRuF_3_ (^1^A’) is of a” symmetry, which is consistent with a widening of the F−M−F angle, whereas the HOMO of NOsF_3_ (^1^A’) is of a’ symmetry, which shows a reduction in the F−M−F angle bisected by the σ plane in *C*_s_ symmetry (Figure 5). The d^1^ metal configuration for the heptavalent tetrafluorides NRu^VII^F_4_ and NOs^VII^F_4_ (*C*_4v_) give rise to a ^2^B_2_ electronic ground state (see Figure S7 for the singly occupied MO).

The effective bond orders^[35]^ (EBOs) for NOsF_3_, NRuF_3_ and NFeF_3_ are 2.8, 2.7 and 2.2, respectively, which in fact corresponds to triple bonds for all these M−N bonds. The computed M−N bond lengths for the novel nitrido compounds (153 pm (FeN), 159 pm (RuN), 163–164 pm (OsN), Figure 7) are close to our published triple bond additive covalent radii: 156 pm (FeN), 157 pm (RuN) and 163 pm (OsN),^[36]^ and also the experimental N−M stretching frequencies (Table 1) support the presence of strong M≡N triple bonds in the novel hexavalent nitrido complexes NM^VI^F_3_. We note that the experimental *ν*(Fe≡N) frequency of NFe^VI^F_3_ of 946 cm^−1^ (Table 1) is not well reproduced by calculations at DFT or CCSD(T) levels (Table S3) and is also overestimated by the more sophisticated NEVPT2 multi‐reference approach (*ν*(Fe≡N)=1027 cm^−1^, Table S3). On the other hand, its comparison with experimental Fe≡N stretching frequencies for pseudo‐tetrahedral N^IV^FeL_3_ complexes, previously reported at 1008 ([Fe^IV^(N)(TIMEN^Mes^)]^+^),^[24c]^ 1028 ([Fe^IV^(N)(PhB(^t^BuIm)_3_]),^[24b]^ and 1034 cm^−1^ ([Fe^IV^(N)‐(PhB(CH_2_P^*i*^Pr_2_)_3_];^[9]^ Table S1) suggests that an increase in the iron oxidation state beyond V does not necessarily lead to a stronger Fe≡N bond.

Table 2 shows experimental M−N stretching frequencies of molecular NMF_3_ species formed by the reaction of NF_3_ with laser‐ablated transition metals. For the d^0^ configurations of all group 4 and group 6 nitrido trifluorides the ideal pseudo‐tetrahedral *C*_3v_ symmetric arrangement was experimentally verified, since there are no electrons in the nonbonding *e*(d_*xy*,*x*2−*y*2_) orbitals that could cause distortions.[^25^, ^33^] The *e* ^3^ configuration of NRhF_3_ and NIrF_3_ leads to Jahn−Teller distorted spin doublet ground states in *C*_s_ symmetry.^[26]^ So far, no experimental data are available for the group 10 derivatives, and for the group 11 analogues only the initial metal insertion products F_2_N−M^II^F were detected after matrix deposition (irradiation of F_2_NCuF led to rearrangement to metastable FN=CuF_2_).^[30c]^

**Table 2:** Experimental M−N stretching frequencies [cm^–1^] of NMF_3_ molecules formed by the reaction of NF_3_ with transition metals of group 4, 6, 8, 9, and 11.

Row	Group 4^[a]^	Group 6^[b]^	Group 8^[c]^	Group 9^[d]^	Group 11^[30c]^
3*d*	596.7 (Ti, *C*_3v_, ^3^A_1_)	1015 (Cr, *C*_3v_, ^1^A_1_)	946.4 (Fe, *C*_3v_, ^3^A_2_)	FN=CoF_2_ only	F_2_N‐CuF, FN=CuF_2_
4*d*	553.1 (Zr, *C*_3v_, ^3^A_1_)	1075 (Mo, *C*_3v_, ^1^A_1_)	1098.5 (Ru, *C*_s_, ^1^A’)	1116.1 (Rh, *C*_s_, ^2^A’) [Ne] 1112.5 [Rh, Ar] ^[e]^	F_2_N−AgF only
5*d*	548.1 (Hf, *C*_3v_, ^3^A_1_)	1091 (W, *C*_3v_, ^1^A_1_)	1140.1 (Os, *C*_s_, ^1^A’) *1086.0 (Os, C*_s_, ^*3*^ *A”)*	1150.4 (Ir, *C*_s_, ^2^A’) [Ne] 1144.6 [Ir, Ar]	F_2_N−AuF only

[a] Ar matrix.^[33]^ [b] Ar matrix.^[25]^ [c] Ne matrix (this work). [d] Ne and Ar matrices.^[26]^ [e] Formation of N≡RhF_3_ along with FN=RhF_2_.

The M−N stretching normal mode of the terminally bond nitrogen ligands of the nitrido trifluorides can regarded to be a good approximation as an almost pure and uncoupled metal‐nitrogen stretching mode that can be used as a measure of the M−N bond strength. The NM^IV^F_3_ derivatives of the group 4 metals possess a singly bonded triplet nitrene (^3^N^−^) ligand, since the ligand cannot oxidize the d^0^ metal center any further. The two unpaired electrons in the N(2p) orbitals are reported to be involved in weak degenerate π bonding interactions for M=Ti≫Zr, Hf.^[33]^ In contrast, the group 6, 8 and 9 NM^VI^F_3_ molecules show a N≡M triple bond with one σ and two π bonds to the terminal nitrido (N^3−^) ligand. The strength and overlap of these bonds increases going down the groups likely due to an improved M(πd)−N(πp) orbital overlap as a result of an increasing relativistic expansion^[37]^ of the 4d and 5d orbitals and the absence of metal core/ligand repulsion proposed in first‐row transition metal compounds.^[38]^ The general trend of increasing N−M bond strength moving along the rows culminates in the highest observed M−N stretching frequency for NIrF_3_. Unexpectedly, this trend does not apply to NFeF_3_ which shows a lower M−N stretching frequency than the group 6 homologue (M=Cr). The lower stability of high‐valent first row late transition metals is well known.[^1a^, ^39^] In the series of 3d NM^VI^F_3_ compounds, for M=Fe it seems we have reached the limit of stability. NCo^VI^F_3_ is not a stable compound and only FNCoF_2_ has been observed experimentally.^[26]^ For the 4*d* element Rh it was found that the rearrangement of the fluoro nitrene complex FNRhF_2_ into N≡Rh^VI^F_3_ is only slightly exothermic (Δ*H* ^0^=−12 kJ mol^−1^, CCSD(T)), which enables the observation of both rearrangement products.^[26]^

Within the atoms in molecules (AIM) scheme^[40]^ the partial negative charge at the nitrido ligand in NM^VI^F_3_ increases from M=Fe to Os (Tables S9 and S13), which indicates a decreasing electron withdrawing effect of the M^VI^F_3_ fragment within this group. For M=Fe and Ru the negative charge at the nitrogen atom also decreases from NMF_2_ (M=Fe: −0.35, Ru: −0.40) to NMF_3_ (M=Fe: −0.25, Ru: −0.35), while for M=Os it remains unchanged (NOsF_2_: −0.50, NOsF_3_: −0.49). As expected, fluorination of NMF_3_ further decreases the atomic charge of the nitrido ligand in NMF_4_ (M=Ru: −0.25, Os: −0.40, Table S9). The high oxidation potential of Fe^VI^ in NFeF_3_ leads to relatively high σ* and π* occupation numbers (0.2 and 0.3 electrons, respectively; Figure S6). These indicates a weakened covalent N−Fe bond, for which the formal N^3−^ nitride notations seems to be a very poor approximation. The occupation of formally antibonding MOs also indicates an oxidation, and thus the onset of a redox non‐innocent behavior of the nitrido ligand.

## Conclusion

The nitrido complexes NFeF_3_, NRuF_3_, NRuF_4_, NOsF_3_ (^1^A’), NOsF_3_ (^3^A”), and NOsF_4_ were shown to be formed by the reaction of free group 8 metal atoms with NF_3_ and established by their characteristic IR spectra recorded in solid neon matrices. Their assignment is supported by observed ^14/15^N isotope shifts and quantum‐chemical predictions. All stretching fundamentals of the NM^VI^F_3_ complexes were confidently assigned. For the *C*_4v_ symmetric NRuF_4_ two distinct bands were confidently assigned, whereas for NOsF_4_ only the strongest band was tentatively assigned. Based on the joint experimental IR and quantum‐chemical analysis the half‐filled *e* ^2^ configuration of NFeF_3_ can be assigned to an undistorted *C*_3v_ structure in a non‐degenerate ^3^A_2_ electronic ground state. NFeF_3_ features an unprecedented low Fe≡N triple‐bond frequency of 946.7 (^14^N≡Fe) and 922.7 cm^−1^ (^15^N≡Fe). The heavier group 8 NMF_3_ homologues are subject to symmetry lowering and spin‐crossover caused by a pseudo Jahn−Teller effect “hidden” in the excited states. While the electronic ground state of NRuF_3_ is a structurally distorted singlet ^1^A′ state (*C*_S_ symmetry), for molecular NOsF_3_ two coexisting distorted *C*_S_ structures with high‐spin and low‐spin d^2^ configurations (magnetic bistability) were detected at 5 K in solid neon. To the best of our knowledge, apart from O_2_Fe(η^2^‐O_2_),[^3^, ^4^, ^5^] no other neutral Fe^VI^ complexes or molecular neutral complexes of Ru^VII^ have yet been reported, and after OsOF_5_,^[1f]^ NOsF_4_ is the second known monomeric Os^VII^ compound.

## Conflict of interest

The authors declare no conflict of interest.

## Supporting information

As a service to our authors and readers, this journal provides supporting information supplied by the authors. Such materials are peer reviewed and may be re‐organized for online delivery, but are not copy‐edited or typeset. Technical support issues arising from supporting information (other than missing files) should be addressed to the authors.

Supporting Information
