# Supplementary material for: High‐Spin Iron(VI), Low‐Spin Ruthenium(VI), and Magnetically Bistable Osmium(VI) in Molecular Group 8 Nitrido Trifluorides NMF3
Source: Chemistry. 2021 Jun 26;27(45):11693–700. doi: 10.1002/chem.202101404 (PMC8457171; doi:10.1002/chem.202101404)
Supplement: Supplementary file 1 — Supporting Information [file CHEM-27-11693-s001.pdf]

Chemistry–A European Journal

Supporting Information

High-Spin Iron(VI), Low-Spin Ruthenium(VI), and Magnetically Bistable Osmium(VI) in Molecular Group 8 Nitrido Trifluorides NMF_3

Tony Stüker, Xiya Xia, Helmut Beckers, and Sebastian Riedel*

Table of Contents

Experimental and Computational Details 2

Experimental Details 2

Computational Details 2

Detailed Assignments 4

$NFeF_3$ 5

$NRuF_3$ and $NRuF_4$ 6

$NOsF_3$ and $NOsF_4$ 7

Supplemental Computational Results 8

$NFeF_3$ 8

Supporting Figures 10

Supplementary Tables 19

Calculated molecular structures and vibrational data 35

Supporting Information References 84

Experimental and Computational Details

Experimental Details

$^{14}NF_3$ and $^{15}NF_3$ (synthesis described elsewhere[1]) were premixed 1:1000 with neon (99.999 %, Linde) in a stainless-steel cylinder. The mixing vessel was connected to a stainless-steel vacuum line connected to a self-made matrix chamber by a stainless-steel capillary. The gas mixture was then co-deposited for 100 min with laser-ablated iron, ruthenium or osmium atoms onto a gold plated copper mirror and cooled to 5 K by using a closed-cycle helium cryostat (Sumitomo Heavy Industries, RDK–205D) inside the vacuum chamber. For the laser-ablation, the 1064 nm fundamental of a Nd:YAG laser (Continuum, Minilite II, 10 Hz repetition rate, 35–50 mJ pulse^{-1}) was focused onto a rotating metal target. Infrared spectra were recorded on a Bruker Vertex 80v with evacuated optical path at 0.1 or 0.5 cm^{-1} resolution in the region 4000–430 cm^{-1} by using a liquid-nitrogen-cooled mercury cadmium telluride (MCT) detector.

Computational Details

Density functional theory (DFT) calculations are performed using the TURBOMOLE 7.0.1 program package[2] employing the GGA, meta-GGA or hybrid exchange-correlation density functionals BP86,[3] M06-L[4] and B3LYP,[5] respectively, with the polarized quadruple-ξ basis set def2-QZVP[6] which applies the Stuttgart-Dresden effective core potential for ruthenium and osmium.[7] The Coupled Cluster Single Double and perturbative Triple excitations (CCSD(T)) calculations are carried out in the spin unrestricted ROHF-UCCSD(T) open-shell coupled cluster formalism[8] using default frozen core settings as implemented in the Molpro 2019 software package.[9] The same software was used for all complete active space self-consistent field (CASSCF)[10] and n-electron valence state perturbation theory (NEVPT2)[11]

calculations. Unless stated otherwise, all CCSD(T) calculations were combined with the augmented triple-ξ basis sets aug-cc-pVTZ (for short: aVTZ) for nitrogen, fluorine, iron[12] and aug-cc-pVTZ-PP (for short: aVTZ-PP) for ruthenium and osmium.[13] Only the valence electrons [N,F: 2p 2s, M: (n-1)d ns] are correlated in the CCSD(T) and NEVPT2 dynamic correlation procedure when the (aug)-cc-pVNZ(-PP/-DK)[12a,12c,13] [for short: (a)VNZ(-PP/-DK)] basis sets are used. Whereas the metal based (n-1)s (n-1)p orbitals were included when the aug-cc-pwCVNZ(-PP/-DK)[12a,12c,13] [for short: awCVNZ(-PP/-DK), N = cardinal number D, T, Q or 5] basis sets are used. The active space for the state-specific complete active-space (SS-CASSCF) reference wavefunctions is chosen to consist of 8 electrons in 8 orbitals covering the Fe≡N three bonding, three antibonding and the two metal centered molecular orbitals. Larger active spaces including the Fe(4s) or Fe–F bonding molecular orbitals of (8,9) (14,11) and (14,12) were also applied. However, the larger active space did either not improve the wavefunction [(8,9)], or the optimization process led to wavefunctions which did not contain the desired molecular orbitals in the active space [(14,11), (14,12)]. NEVPT2 calculations for $NFeF_3$ were carried out with relativistic corrections using the second order Douglas-Kroll-Hess Hamiltonian.[14] Three diagnostic criteria, T_1, D_1 and the %TAE_e[(T)] from CCSD(T) calculations were examined for all NMF_3 compounds under consideration. Except $NFeF_3$, all values shown in Table S7 are well within the limits of $T_1 \leq 0.05$, $D_1 \leq 0.15$ and %TAE $\leq$ 10 for 3d transition metal (TM) containing species[15] and $T_1 < 0.045$, $D_1 < 0.120$ and %TAE < 10 for 4d TM containing species.[16] The $NOsF_3$ $^1A'$/ $^3A''$ minimum energy crossing point (MECP) was calculated at the UB3LYP/def2-TZVP[6b] and UHF-UCCSD(T)/cc-pVTZ(Os: cc-pVTZ-PP) level of theory using SurfCrossOpt as implemented in ORCA 4.2.0.[17] Harmonic vibrational frequency calculations were carried out for optimized structures analytically (BP86,

B3LYP, M06-L) or numerically (CCSD(T) and NEVPT2, step size of numerical differentiation: 0.01 a.u.) The $NFeF_3$, $NRuF_3$ and $NOsF_3$ APES scans were carried out with the same active space as described above in C_s point group symmetry. For $NFeF_3$ 9 electronic states ($3 \times {}^1A'$, $2 \times {}^1A''$, $2 \times {}^3A'$ and $2 \times {}^3A''$), for $NRuF_3$ and $NOsF_3$ 8 electronic states ($2 \times {}^1A'$, $1 \times {}^2A''$, $2 \times {}^3A'$ and $3 \times {}^3A''$) were averaged with equal weights with subsequent NEVPT2 treatment of all states individually. The Atoms in Molecules (AIM) charges[18] (Tables S8 and S12) were calculated using Multiwfn 3.6.[19] The NLMO/NPA Bond Orders,[20] Natural Charges (NPA)[20] and Wiberg Bond Indices[21] (Tables S8 and S12) were calculated using the NBO 7.0 software.[22] All rendered figures showing molecular structures and orbitals were obtained using the vmd 1.9.2 program.[23]

Detailed Assignments

By comparing spectra of the reaction products of different metal targets, metal dependent bands were identified. Further simplification was achieved by taking into account band positions of molecular binary fluorides MF_n, which were obtained by recording complementary spectra of the reaction products of metal atoms with elementary fluorine under the same conditions described above for the reaction with NF_3. For each experiment those bands were selected which are absent in the spectra obtained with other metal targets and which could not be assigned to the binary metal fluorides NF_n. Only this set of selected bands can be considered for the assignment of any novel species formed in this particular experiment. Their further assignment is based on the observed $^{14/15}N$ isotope shift and on their similar behavior in different experiments, such as e.g. in annealing or photolysis experiments. Only a few bands

from this selected set of bands could not be assigned to a NMF_n (n = 3, 4) species. They remained unassigned and were labelled e.g. with a diamond in each spectrum.

Photolysis experiments were conducted using LED light of λ = 730, 656, 617, 590, 528, 470, 455 and 273 nm and subsequently ArF excimer laser light of λ = 193 nm.

$NFeF_3$

The formation of molecular $NFeF_3$ (C_{3v}) is proved by the assignment of all stretching vibrations marked **A**, **B** and **C** at 946.4, 766.8 and 658.8 cm^{-1} in the IR spectrum obtained after co-depositing laser-ablated iron and NF_3 in excess neon shown in Figure S1 (see also Table S3). The FeF_3 stretching modes split into a degenerate e and an a_1 component at 766.8 and 658.8 cm^{-1}, respectively. The latter shows a small $^{14/15}N$ isotopic shift of −1.1 cm^{-1}. The corresponding bands for the group 6 $NCrF_3$ congener of C_{3v} point group symmetry (798 cm^{-1} (e), 709 cm^{-1} (a_1))[24] are located about 30 cm^{-1} and 50 cm^{-1}, respectively, higher than those assigned to $NFeF_3$. This redshift is the result of a weakening of the M-F bonds due to the partial e^2 occupation of the Fe centered orbitals that are slightly antibonding with respect to these bonds. This is further supported by the computed, slightly elongated Fe-F bond distances (Cr–F: 170 pm, Fe–F: 172 pm). The vibrational *e*-type mode of $NFeF_3$ exhibits a small splitting in two components (766.8 cm^{-1} and 766.7 cm^{-1}) with different intensities, which indicates that the degeneracy is moderately lifted, most likely due to different orientations of the $NFeF_3$ molecule within the matrix cavity. The Fe–N stretch at 946.4 cm^{-1}, marked **A** in Figure S1, shows a $^{14/15}N$ isotopic shift of −23.7 cm^{-1} (isotopic ratio of 1.0257). The band position of the previously reported cationic complex $[(TIMEN^R)Fe^{IV}(N)]BPh_4$ (R = xylyl, mesityl)[25] is with 1008 cm^{-1} higher, but (within given precision) exhibits the same isotopic ratio. Quantum-chemical calculations at the NEVPT2 level overestimate the N-Fe band position, but the predicted isotopic ratio of

1.0256 is very close to the experimentally observed value of 1.0257. Known bands at 744.7/744.7, 752.6 and 785.1 cm^{-1} were assigned to the molecular binary iron fluorides $^{56}FeF_3$, $^{56}FeF_2$ and $^{54}FeF_2$, respectively.[26]

$NRuF_3$ and $NRuF_4$

The spectra recorded after co-depositing laser-ablated ruthenium and NF_3 in excess of neon (Figure S2), clearly revealed the presence of two different nitrido ruthenium complexes, finally assigned to $NRuF_3$ (C_s) and $NRuF_4$ (C_{4v}). Their characteristic Ru≡N stretching bands are labeled **A** (1105.4 cm^{-1}, $NRuF_3$) and **A'** (1098.5 cm^{-1}, $NRuF_4$) in Figure S2. These bands show the same $^{14/15}N$ isotopic ratio of 1.0305 (Table S3). Their isotopic pattern due to seven naturally occurring, stable ruthenium isotopes obtained from spectra recorded at 0.1 cm^{-1} are listed in Table S4 and compared in Figure S4 with the predicted pattern. For the **A'** band the $^{96/104}Ru$ isotopic ratio were not experimentally determined, because of a low natural abundance (5.52 %) of the $N^{96}RuF_4$ isotopologue. $NRuF_4$ is likely formed by the exothermic addition of a fluorine atom to $NRuF_3$ (see Table S1).

The RuF_3 stretching modes of C_s symmetric $NRuF_3$ split into three modes. The strong antisymmetric F–Ru–F appears at 668.5 cm^{-1} (labeled **B** in Figure S2) and likely overlaps with the nearby weaker F'–Ru band. A control experiment in which laser-ablated Ru was deposited with F_2 instead of NF_3 revealed that also a band associated with a ruthenium fluoride might contribute to the strong **B** absorption. The symmetric F–Ru–F mode is attributed to the band labelled **C** at 635.8 cm^{-1}. From the RuF_4 stretching modes of $NRuF_4$ only the strongest band, the degenerate e-type mode could safely be assigned to the band labeled **B'** in Figure S2 centered at 700.0 cm^{-1}. The much weaker predicted RuF_4 a_1 band was not detected, probably because it is either too weak or overlapped by another band. The high-resolution spectrum shown

in Figure S5 reveals a distinct ruthenium isotopic pattern for all these three assigned Ru–F stretching modes (Tables S5 and S6). The band positions and isotopic pattern observed for $NRuF_4$ can be compared well with those reported for $ORuF_4$ isolated in solid nitrogen.[27] The reported ^{102}Ru≡O stretching mode (1059.5 cm^{-1}) is red-shifted by about 39 cm^{-1} with respect to ν(^{102}Ru≡N) (1098.5 cm^{-1}, Table S3), while the RuF_4 e-type mode is blue-shifted by about 10 cm^{-1}. Considering the greater mass of oxygen and the more interacting nitrogen matrix, the small red-shift suggests very similar bonding in both species. In fact, the bond order of Ru≡O in $ORuF_4$ is predicted to be three as well.[28]

A few Ru–F stretching bands remained unassigned. The $^{96/104}$Ru isotopic ratio of the band labeled by a plus sign at 651.9 cm^{-1} in Figure S2 exhibits a characteristic ruthenium isotopic pattern expected for a mononuclear Ru complex, and the two bands labeled with a pound sign at 606.5 and 616.5 cm^{-1} show a different isotopic pattern, probably caused by a Ru dimer complex.

$NOsF_3$ and $NOsF_4$

The metal-nitrogen stretching region of the spectra obtained by co-depositing of laser-ablated osmium and $^{14}NF_3$ contains one band labeled **A** in Figure S3 at 1140.1 cm^{-1}, while the spectra obtained using isotopic label $^{15}NF_3$ contains two N–Os stretching bands. The first band at 1104.6 cm^{-1} is caused by the ^{15}N isotopologue of **A** and shows a $^{14/15}$N isotopic shift of −35.5 cm^{-1} and a $^{14/15}$N isotopic ratio of 1.03214. By applying the isotopic ratio obtained for **A** to the second band at 1086.0 cm^{-1}, we obtain 1120.9 cm^{-1} as an estimated value for the ^{14}N isotopologue of the second band labeled **A’** in Figure S3. This band is overlapped by the stronger band associated with the ^{14}NF radical. Both bands are assigned to $NOsF_3$ in two electronic states. Band **A**

is assigned to the Os≡N stretching mode of $NOsF_3$ in the $^1A'$ ground state, and band **A'** to $NOsF_3$ in a near-by excited $^3A''$ electronic state. The Os–F stretching bands assigned to $NOsF_3$ ($^1A'$) are labeled **B**, **C** and **D** in Figure S3 and are at 686.0, with a matrix site at 686.6 cm^{-1}, at 641.3 with a weaker matrix site at 640.1 cm^{-1} and at 632.3 cm^{-1}, respectively. Bands **B** and **D** are assigned to the symmetric and antisymmetric F–Os–F stretching mode, respectively, and **C** to the F'–Os stretching mode. Bands labeled **B'**, **C'** and **D'** at 675.8 cm^{-1} (with a matrix site at 677.0 cm^{-1}), 660.5 cm^{-1} (matrix site at 658.9 cm^{-1}), and 607.4 cm^{-1}, respectively, are assigned to $NOsF_3$ ($^3A''$). Finally, the band labeled **A"** at 689.6 cm^{-1} is tentatively assigned to the OsF_4 e-type mode of $NOsF_4$ in C_{4v} symmetry. The N–Os stretching band and the remaining Os–F stretching bands of $NOsF_4$ are predicted with significantly lower intensity and were not detected. The intensity of the bands assigned to $NOsF_3$ and $NOsF_4$ did not change in annealing and photolysis experiments. All assignments and isotopic shifts are in excellent agreement with values obtained at the CCSD(T) level of theory summarized in Table S3.

Supplemental Computational Results

$NFeF_3$

The B3LYP hybrid functional was found to perform worst when comparing the Fe–N bond length and stretching frequency with results from other single-reference methods (DFT and CCSD(T)) or multi-reference NEVPT2 calculations (Table S2). To obtain reliable structural and vibrational data for $NFeF_3$ by taking dynamical, non-dynamical and core-valence correlation effects, as well as scalar relativistic effects into account the NEVPT2/CASSCF(8,8)/aVTZ-DK(Fe: awCVTZ-DK) method was employed. The

rearrangement of the iron fluoroimino intermediate, FN=FeF_2, to the high-valent iron nitrido trifluoride N≡FeF_3 is predicted to be exothermic by only −7 kJ mol^{-1} at the B3LYP level (Table S1), while the GGA and meta-GGA functionals BP86 and M06-L predict more exothermic reaction enthalpies of −78 and −74 kJ mol^{-1}, respectively. The latter predictions agree better with the NEVPT2 results (Table S2). CASSCF(8,8) calculations reveal that the leading configuration of $NFeF_3$ is $\sigma^2\pi^2\pi^2\delta^1\delta^1\pi^{*0}\pi^{*0}\sigma^{*0}$, which contributes only 60 % to the $^3A_2$ ground state. The small ligand field splitting, i. e. the low energy gap between the non-bonding 3d(Fe,δ)-MOs and the antibonding MOs indicates a poor overlap between the 3d(Fe) orbitals with the N^{3-} ligand orbitals,[29] and leads to a significant population of Fe≡N antibonding π^* and σ^* orbitals (Figure S6). The effective bond order[30] (EBO) of the individual bonds is 0.8 (σ) and 0.7 (π), a total of 2.2, or an integer BO of 3. The NLMO bond order of 2.30 and the Wiberg bond index of 2.52 obtained from analyzing the DFT wavefunction (Table S8) further support the assignment of a Fe≡N triple bond. The short calculated Fe≡N bond length of 153 pm is within the range of reported crystallographic data for iron complexes with triply bonded nitride ligands of 153 pm ([$(TIMEN^R)Fe^{IV}(N)$]BPh_4, R = xylyl, mesityl),[25] 151 pm ([$PhB(^tBuIm)_3Fe^{IV}(N)$])[31] and 150–151 pm ([$PhB(^tBuIm)_3Fe^{V}(N)$]$BarF_{24}$)[31], or 157 pm ([$(Me_3cyac)Fe^{VI}(N)$]$(PF_6)_2$, derived from EXAFS data)[32].

Supporting Figures

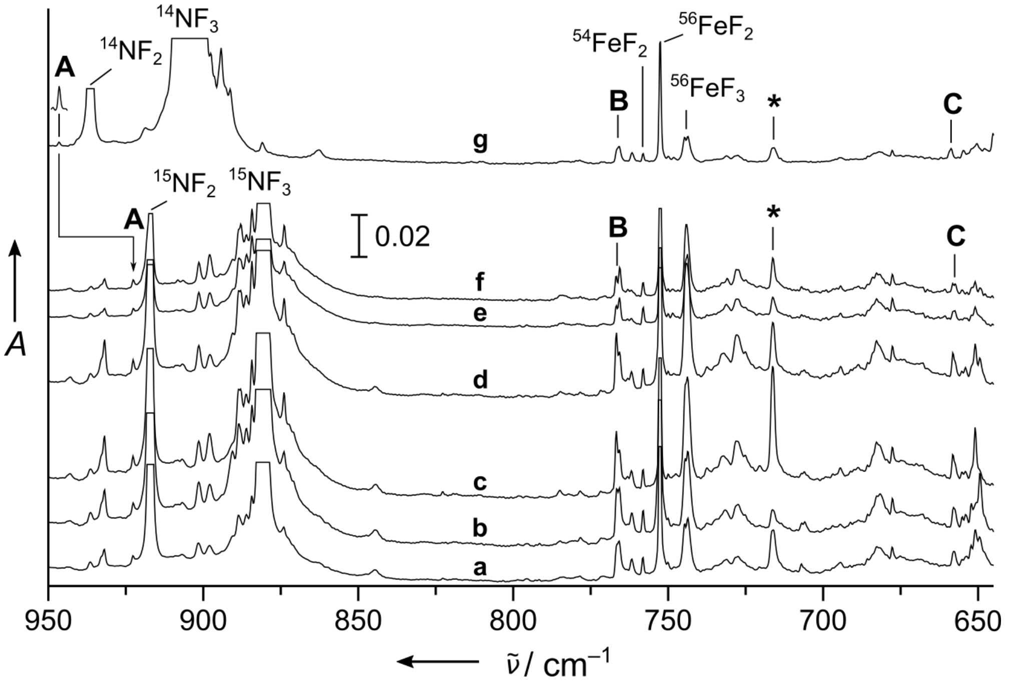

Figure S1. IR spectra of laser ablated iron co-deposited with 0.1 % $^{15}NF_3$ in Ne (**a**), after 10 min irradiation using LED light of λ = 656±10 nm (**b**), 470±10 nm (**c**), 273±10 nm (**d**), as well as a 193 nm excimer laser radiation (**e**), and successive annealing to *T* = 10 K (**f**). Experiment using 0.1 % $^{14}NF_3$ in Ne (g). Bands labeled **A**, **B** and **C** are assigned to $NFeF_3$ (see Table S3). Band **A** is by a factor of five in spectrum **g**. Known bands of binary iron fluorides[26] are indicated, and an unassigned band showing no$^{14/15}$N isotopic shift is marked with an asterisk. Note that $NFeF_3$ is unaffected by annealing to 10 K or the selective LED radiations, but this band slightly gains intensity upon irradiation with LED light with λ = 656 nm and lose intensity when irradiated with laser light of λ = 193 nm.

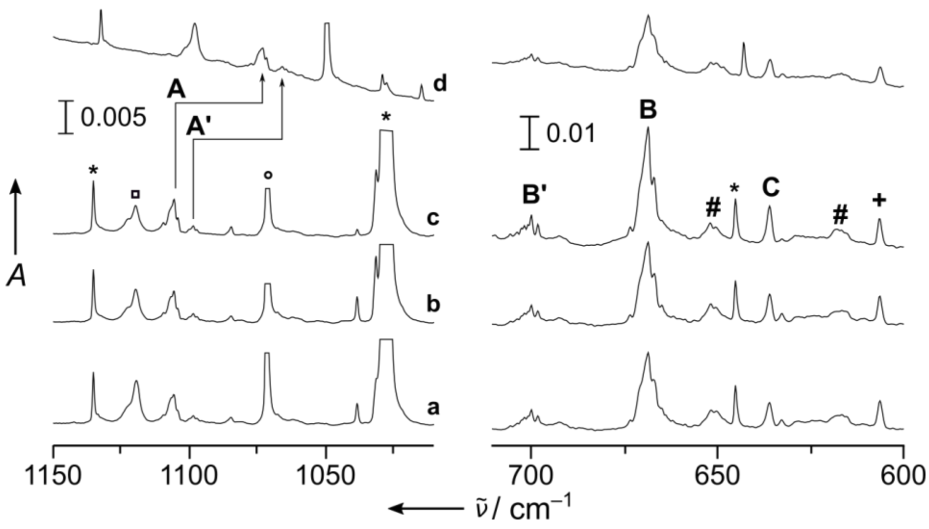

Figure S2. IR spectra of laser ablated ruthenium co-deposited with 0.1 % $^{14}NF_3$ in Ne (**a**), after annealing to 10 K (**b**), and broadband photolysis (**c**). Co-deposition of Ru with 0.1 % $^{15}NF_3$ in Ne (**d**). Bands labeled **A** – **C** are attributed to $NRuF_3$ and **A'** and **B'** are due to $NRuF_4$. Unknown product bands are labeled by a pound and plus sign, respectively. The bands associated with ^{14}NF, $^{14}NF_2$ and $^{14}NF_3$ are marked with squares, circles and asterisks, respectively.[33]

Note that the $^{96/104}Ru$ isotopic ratio of the band labeled by a plus sign at 651.9 cm^{-1} exhibits a characteristic ruthenium isotopic pattern expected for a mononuclear Ru complex, while the two bands labeled with a pound sign at 606.5 and 616.5 cm^{-1} show a different isotopic pattern, probably caused by a Ru dimer complex.

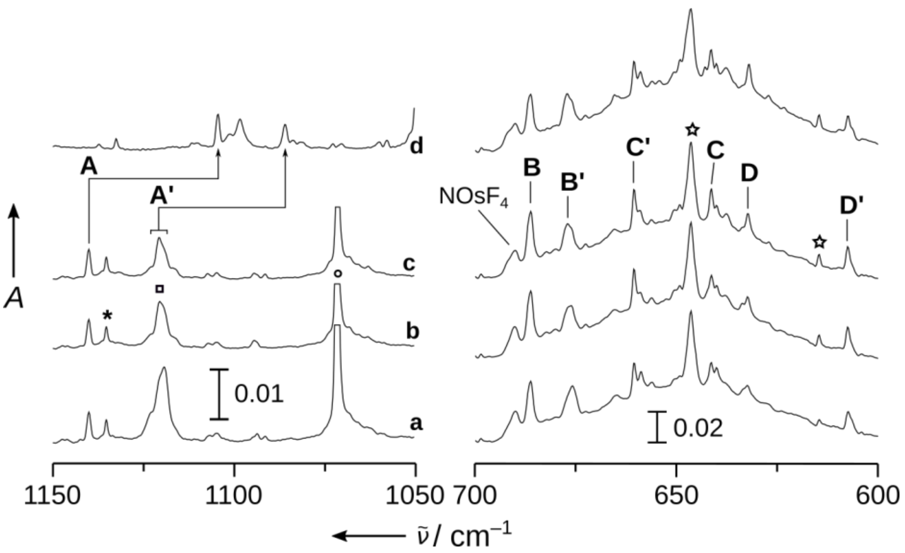

Figure S3. IR spectra of laser ablated osmium co-deposited with 0.1 % $^{14}NF_3$ in Ne (**a**), after annealing to 10 K (**b**), and broadband photolysis (**c**). Co-deposition of Os with 0.1 % $^{15}NF_3$ in Ne (**d**). Bands labeled **A**–**D** are attributed to $NOsF_3$ and **A'**–**D'** to $NOsF_3$ (^{3}A"). Bands of binary osmium fluorides are marked with star symbols. The bands associated with ^{14}NF, $^{14}NF_2$ and $^{14}NF_3$ are marked with squares, circles and asterisks, respectively.[33]

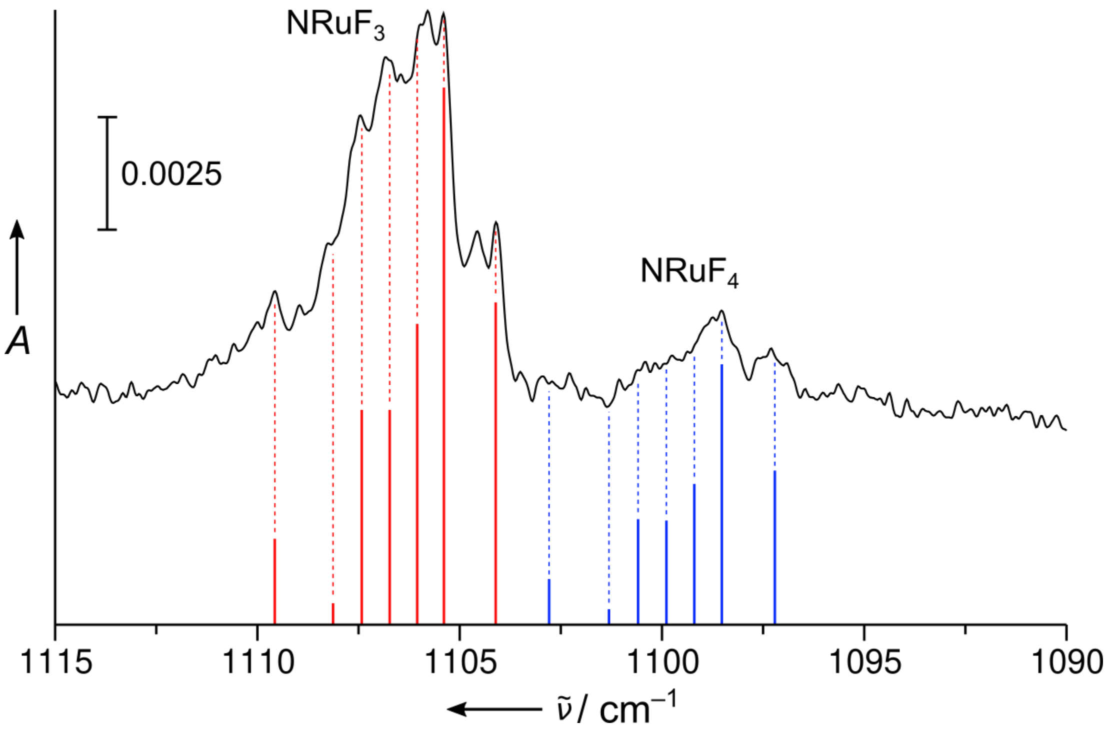

Figure S4. Infrared absorption spectrum of laser ablated ruthenium with 0.1 % NF_3 displaying the N-Ru stretching bands of $NRuF_3$ and $NRuF_4$ in a spectral resolution of 0.1 cm^{-1}. The pattern is caused by the seven naturally occurring, stable ruthenium isotopes (see Table S4).

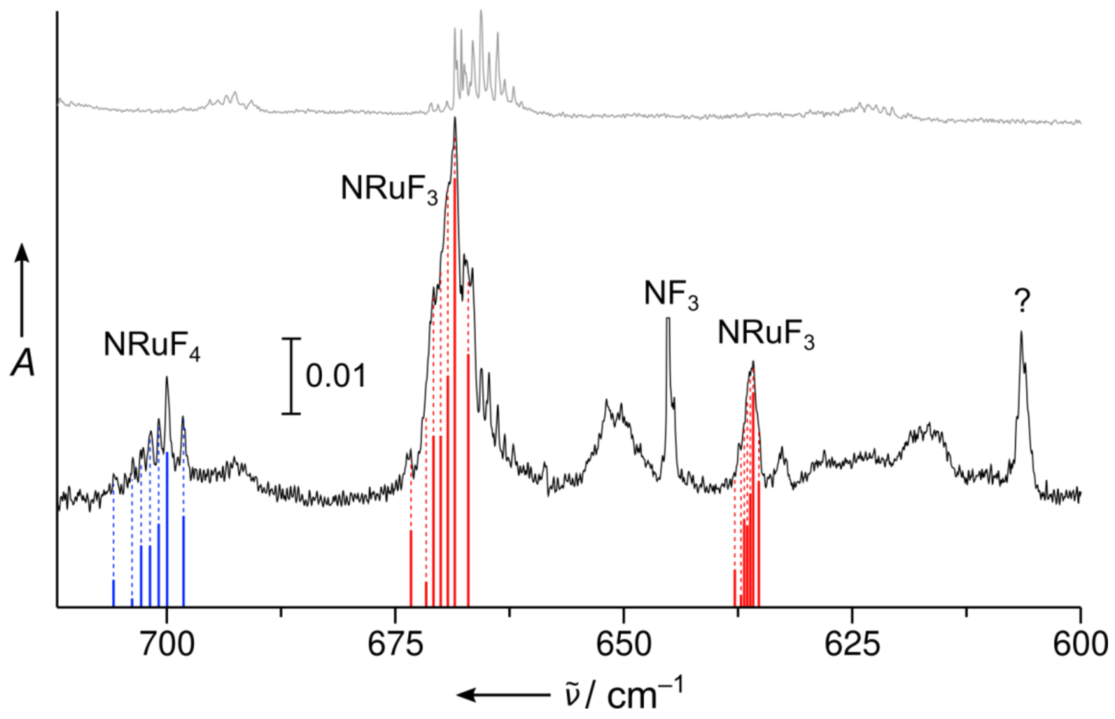

Figure S5. Infrared absorption spectrum of laser ablated ruthenium with 0.1 % NF_3 displaying the RuF stretching bands of $NRuF_3$ and $NRuF_4$ in a spectral resolution of 0.1 cm^{-1}. The pattern is caused by the seven naturally occurring, stable ruthenium isotopes (see Tables S5 and S6).

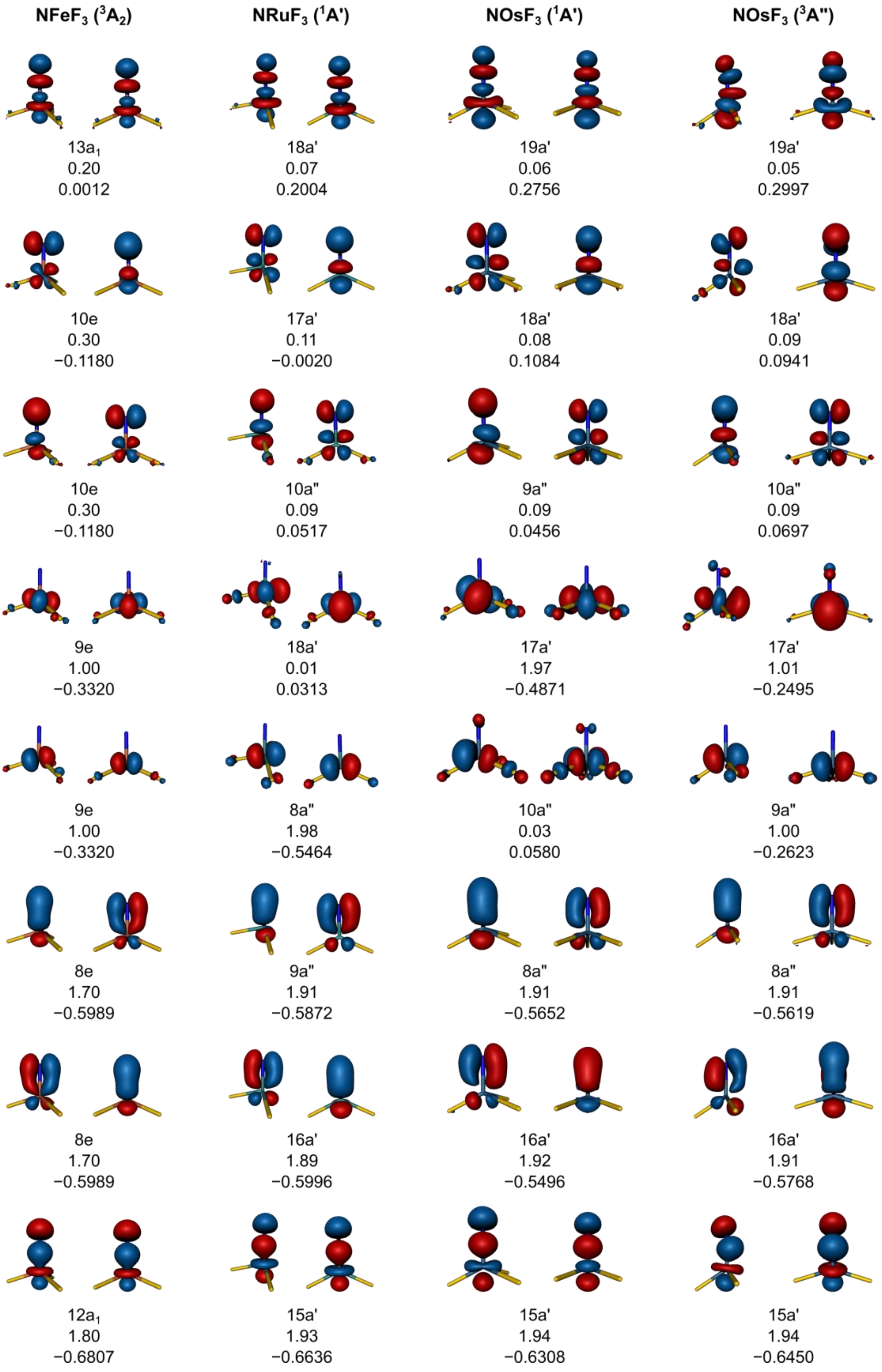

Figure S6. Active space natural molecular orbital plot at σ = 0.1 e Bohr^{-3}, irreproducible representations, occupation numbers and orbital energies (hartree) of $NFeF_3$ ($^3A_2$), $NRuF_3$ ($^1A'$), $NOsF_3$ ($^1A'$) and $NOsF_3$ ($^3A''$) calculated at the CASSCF(8,8)/aug-cc-pVTZ(-PP) level of theory at the

CCSD(T)/aug-cc-pVTZ-(-PP) (M = Ru, Os) or NEVPT2/aug-cc-pwCVTZ-DK (M = Fe) optimized ground state structures. For the singlet states of $NRhF_3$ and $NOsF_3$ a state-averaging procedure (Molpro: “states,2;weight,0.9,0.1”) was applied to include the desired orbitals into the active space. Two columns are shown for each species: In the right column the molecules are shown in the xz plane, whereas they are shown in the yz plane in the left column. The molecular orbitals for each species are ordered according to those of $NFeF_3$ as reference.

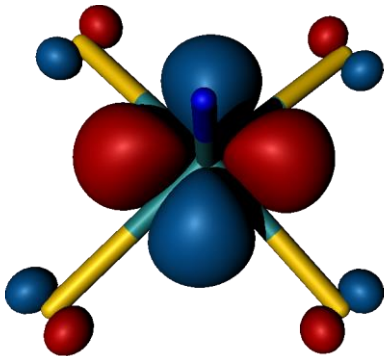

Figure S7. Singly occupied molecular orbital (SOMO) plot of $NRuF_4$ at σ = 0.1 e $Bohr^{-3}$ obtained at the ROHF/aug-cc-pVTZ(Ru: aug-cc-pVTZ-PP) level of theory.

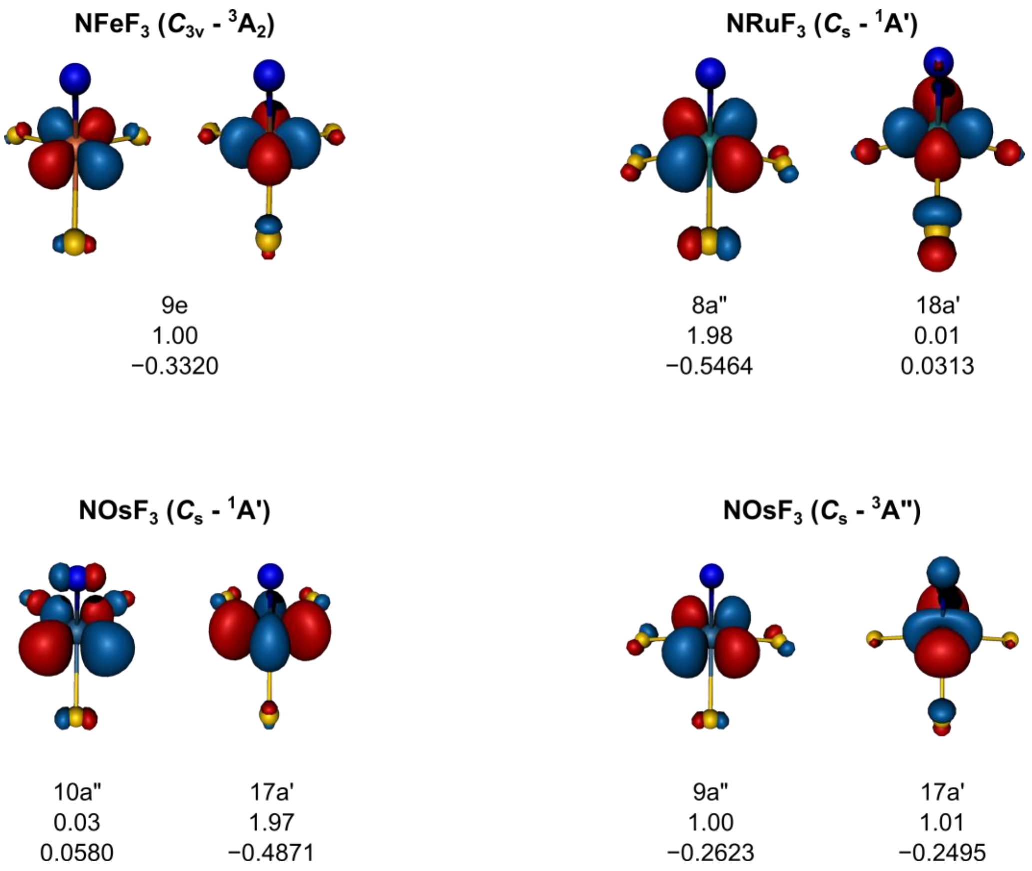

Figure S8. Plot of the metal centered e-type natural molecular orbitals of NMF_3 at $\sigma = 0.1$ e Bohr^{-3}, irreproducible representations, occupation numbers and orbital energies (hartree) of $NFeF_3$ ($^3A_2$), $NRuF_3$ ($^1A'$), $NOsF_3$ ($^1A'$) and $NOsF_3$ ($^3A''$) calculated at the CASSCF(8,8)/aug-cc-pVTZ(-PP) level of theory at the CCSD(T)/aug-cc-pVTZ-(-PP) (M = Ru, Os) or NEVPT2/aug-cc-pwCVTZ-DK (M = Fe) optimized ground state structures. For the singlet states of $NRhF_3$ and $NOsF_3$ a state-averaging procedure (Molpro: "states,2;weight,0.9,0.1") was applied to include the desired orbitals into the active space.

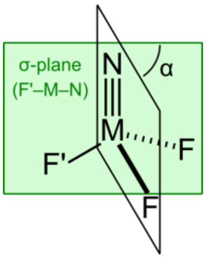

Figure S9. Dihedral angle α between the F’–M–N σ-plane and a F–M–N plane.

Supplementary Tables

Table S1. Experimental Fe−N distances (Å) and stretching fundamentals (cm^{-1}) of representative high-valent iron nitride complexes.

**Complex**	**Fe−N distances (Å)**	**Fe−^{14}N stretch (cm^{-1})**
$[Fe^{V}N(OEP)]$ [a]		876 (RR)
$[Fe^{V}N(TPP)]$ [a]		876 (RR)
$[Fe^{V}N(TTPPP)]$ [b]		876 (RR)
$[Fe^{V}(N)(Me_2Py_2TACN)]^{2+}$ [c]		866 (IRPD)
$[Fe^{V}(N)(MePy_2TACN)](PF_6)_2$ [34]	1.64(1) (EXAFS)	855 (IRPD)
$[Fe^{V}(N)(cyclam\text{-}ac)]^{+}$ [d]	1.61(1) (EXAFS)	864 (NRVS)
$[Fe^{VI}(N)(Me_3cyclam\text{-}ac)]^{2+}$ [32]	1.57(2) (EXAFS)	
$[Fe^{IV}(N)(PhB(CH_2P^{i}Pr_2)_3]$ [35]	1.51–1.55 (EXAFS)	1034
$[Fe^{IV}(N)(P_3^{B})]^{+}$ [e]	1.54(2) (EXAFS)	
$[Fe^{IV}(N)(PhB(^{t}BuIm)_3)]$ [36] [f]	1.532(5) (X-Ray)	
$[Fe^{IV}(N)(PhB(^{t}BuIm)_3)]$ [37]	1.512(1) (X-Ray)	1028 (RR)
$[Fe^{V}(N)(PhB(^{t}BuIm)_3)]^{+}$ [31]	1.506(2)/1.502(2) (X-Ray)	
$[Fe^{IV}(N)(TIMEN^{Mes})]^{+}$ [g] [25]	1.526(2) (X-Ray)	1008
$[Fe^{IV}(N)(TIMMN^{Mes})]^{+}$ [38]	1.513(3) (X-Ray)	
$Fe^{V}(N)(TIMMN^{Mes})]^{2+}$ [38]	1.529(1) (X-Ray)	

[a]: RR: Resonance Raman (OEP^{2-}: octaethylporphinato dianion, TPP^{2-}: tetraphenylporphinato dianion).[39] ν(M=N) of stable nitridomanganese(V) porphyrins and nitridochromium(V) porphyrins were observed at 1049–1052 cm^{-1} for the former[40] and at 1017 cm^{-1} for the latter.[41]

[b]: RR: Resonance Raman ($TTPPP^{2-}$ = tetrakis-(2,4,6-triphenylphenyl)porphyrinato dianion).[42]

[c]: (Me_2Py_2tacn): 1-(di(pyridin-2-yl) methyl)-4,7-dimethyl-1,4,7-triazacyclononane, IRPD: infrared photodissociation;[34b]

[d]: NRVS: nuclear resonant vibrational spectroscopy.[43]

[e]: P_3^{B}:= tris(o-diisopropylphosphinophenyl)borane.[44]

[f]: $PhB(^{t}BuIm)_3)^{-}$ = phenyltris(3-tert-butylimidazol-2-ylidene.

[g]: $TIMEN^{Mes}$ = tris[2-(3-mesityl-imidazol-2-ylidene)ethyl]-amine.

[h]: $TIMMN^{Mes}$ = tris-[2-(3-mesityl-imidazol-2-ylidene)methyl]-amine.

Table S2. Selected reaction enthalpies at T = 0 for MNF_n compounds (n = 1–3 for M = Fe and n = 1–4 for M = Ru, Os) obtained at the B3LYP, BP86 and M06-L (Fe only) levels of theory.[a]

Reaction		ΔH^0 [kJ mol^{-1}]		
		**B3LYP**	**BP86**	**M06-L**
Fe + NF_3	→ F_2NFeF ($^3A'$)	−331	−391	−351
F_2NFeF ($^3A'$)	→ $FNFeF_2$ ($^3A''$)	−239	−246	−267
$FNFeF_2$ ($^2A''$)	→ $NFeF_3$ (C_{3v}, $^3A_2$) [a]	−4	−78	−74
$NFeF_3$ ($^3A_2$ – C_{3v})	→ $NFeF_2$ ($^2A_2$) + F	277	327	324
$NFeF_3$ ($^3A_2$ – C_{3v})	→ $NFeF_2$ ($^2A_2$) + ½ F_2	204	224	250
$NFeF_3$ ($^3A_2$ – C_{3v})	→ NFeF ($^1A'$) + F_2	597	592	604
$NFeF_3$ ($^3A_2$ – C_{3v}) + F	→ $NFeF_2$ ($^2A_2$) + F_2	131	121	177
Ru + NF_3	→ F_2NRuF ($^3A''$)	−386	−439	-
F_2NRuF ($^3A''$)	→ $FNRuF_2$ ($^3A''$)	−238	−258	-
$FNRuF_2$ ($^3A''$)	→ $NRuF_3$ (C_{3v}, $^3A_2$)	−153	−162	-
$FNRuF_2$ ($^3A''$)	→ $NRuF_3$ ($^1A'$)	−143	−146	-
$NRuF_3$ ($^1A'$) + F	→ $NRuF_4$ (C_{4v}, $^2B_2$)	−305	−371	-
$NRuF_3$ ($^3A''$)	→ $NRuF_2$ (C_{2v}, $^2A_2$) + F	286	332	-
$NRuF_3$ ($^3A''$)	→ $NRuF_2$ (C_{2v}, $^2A_2$) + ½ F	213	232	-
$NRuF_3$ ($^3A''$)	→ NRuF ($^1A'$) + F_2	535	577	-
$NRuF_3$ ($^3A''$) + F	→ $NRuF_2$ (C_{2v}, $^2A_2$) + F_2	140	129	-
Os + NF_3	→ F_2NOsF (C_{2v}, $^3A_1$)	−413	−474	-
F_2NOsF ($^3A_1$ - C_{2v})	→ $FNOsF_2$ ($^3A''$)	−295	−299	-
$FNOsF_2$ ($^3A''$)	→ $NOsF_3$ ($^1A'$)	−196	−193	-
$FNOsF_2$ ($^3A''$)	→ $NOsF_3$ ($^3A''$)	−206	−208	-
$NOsF_3$ ($^3A''$)	→ $NOsF_2$ ($^3A''$) + F	288	330	-
$NOsF_3$ ($^3A''$)	→ $NOsF_2$ ($^3A''$) + ½ F	215	227	-
$NOsF_3$ ($^3A''$)	→ NOsF ($^1A'$) + F_2	674	686	-
$NOsF_3$ ($^3A''$) + F	→ $NOsF_2$ (C_{2v}, $^2A_1$) + F_2	142	124	-

[a] Unless stated otherwise, complexes were optimized in *C*s point group symmetry.
[b] ROB3LYP/def2-QZVP: −31 kJ mol^{-1}, CCSD(T)/aug-cc-pVTZ-DK: −66 kJ mol

Table S3. Comparison of the computed N–Fe stretching frequency (ν(Fe–N) in cm^{-1}) and bond length (r(Fe–N) in pm) in $NFeF_3$ obtained at spin unrestricted and restricted open-shell DFT, CCSD(T) and NEVPT2 levels of theory.

**Method**	**ν(Fe–N)**	**r(N–Fe)**	**SC / T_1 / C_0^2 [a]**
UB3LYP	421	163.8	1.343
UM06-L	785	154.5	0.556
UBP86	1096	151.1	0.070
ROB3LYP [b]	1192	148.0	-
CCSD(T)	757	161.2	0.101
NEVPT2	1028	153.3	0.599

[a] Diagnostics of multi-reference character: SC (spin contamination) for unrestricted DFT methods UB3LYP, UM06-L and UBP86, T_1 diagnostic for CCSD(T) and the weight of the leading configuration C_0^2 of the CASSCF wave function. [b] Calculated using Molpro 2019.

Table S4. Calculated and experimental vibrational wavenumbers (ν(^{14}N) in cm^{-1}) and $^{14/15}$N isotopic shifts ($\Delta\nu$ in parentheses) for $NFeF_3$, $NRuF_3$, $NRuF_4$, $NOsF_3$ and $NOsF_4$.

**Exp.** [a]	**BP86** [b]	**B3LYP** [b]	**CCSD(T)** [b]	**Assignment**
		**$NFeF_3$ (C_{3v}, $^3A_2$)** [c]		
946.4 (−23.7)	1096 (−28) [34]	421 (−12) [21]	1028 (−26) [-] [d]	NFe str., a_1
766.8 (0) / 766.7 (0)	691 (0) [2 × 100]	701 [2 × 146]	737(0) [-] [d]	FeF_3 str., e
658.8 (−1.1)	650 (−1) [40]	683 (1) [33]	689 (−2) [-] [d]	FeF_3 str., a_1
		**$N^{102}RuF_3$ (C_s, ^{1}A')**		
1105.4 (−32.7)	1150 (−34) [40]	1202 (−35) [43]	1085 (−32) [-]	NRu str., a'
[e]	651 (0) [46]	669 (0) [54]	682 (0) [-]	F'-Ru str., a'
668.5 (0)	641 (0) [150]	661 (0) [171]	678 (0) [-]	antisym. F-Ru-F str., a"
635.8 (0)	618 (0) [48]	634 (0) [58]	649 (0) [-]	sym. F-Ru-F str., a'
		**$N^{102}RuF_4$ (C_{4v}, $^2B_1$)**		
1098.5 (−32.5)	1060 (−31) [16]	1175 (−35) [15]	1080 (−32) [-]	NRu str., a_1
700.1 (0)	675 (0) [2 × 162]	694 (0) [2 × 196]	711 (0) [-]	RuF_4 stretch, e
- [e]	645 (0) [22]	670 (0) [27]	681 (0) [-]	RuF_4 stretch, a_1
- [f]	576 (0) [0]	588 (0) [0]	598 (0) [-]]	RuF_4 stretch, b_2
		**$NOsF_3$ (C_s, ^{1}A')**		
1140.1 (−35.5)	1148 (−36) [23]	1196 (−38) [28]	1152 (−36) [-]	NOs str., a'
686.0 / 686.6 (0)	657 (0) [98]	671 (0) [94]	689 (0) [-]	OsF_2 sym. str., a'
641.3 / 640.0 (0)	637 (0) [45]	647 (0) [75]	664 (0) [-]	OsF' sym. str., a'
632.3 (0)	609 (0) [51]	629 (0) [59]	652 (0) [-]	OsF_2 antisym. str., a"
		**$NOsF_3$ (C_s, ^{3}A")**		

1086.0 (-) [f]	1089 (-35) [27]	1130 (-37) [31]	1095 (-36) [-]	^{15}NOs str, a’
675.8 / 677.0	646 (0) [134]	657 (0) [153]	675 (0) [-]	OsF_2 antisym. str., a”
660.5 / 658.9 (0)	637 (0) [36]	652 (0) [50]	668 (0) [-]	OsF_2 sym. str., a’
607.4 (0.0)	581 (0) [66]	594 (0) [75]	614 (0) [-]	OsF’ sym. str., a’
		**$NOsF_4$ (C_{4v}, $^2B_1$)**		
- [g]	1140 (−36) [13]	1192 (−37) [13]	1145 (−36) [-]	NOs str., a_1
- [g]	664 (0) [25]	687 (0) [31]	706 (0) [-]	OsF_4 stretch, a_1
689.9 (0)	658 (0) [2 × 152]	674 (0) [2 × 176]	693 (0) [-]	OsF_4 stretch, e
- [h]	605 (0) [0]	617 (0) [0]	635 (0) [-]	OsF_4 stretch, b_2

[a] Neon matrix; matrix sites are separated by a slash. [b] Intensities in km mol^{-1} in square brackets. [c] M06-L/def2-QZVP: 785 a_1 (−11) [12], 703 e (0) [200], 617 a_1 (−1) [40]. [d] NEVPT2/aug-cc-pwCVTZ-DK; [f] ν(^{15}N–Os) in cm^{-1}, see text. [e] Band is likely hidden by the stronger antisymmetric F-Ru-F stretching mode (a’’). [g] Too weak or overlapped. [h] Not IR active.

Table S5. The experimental isotopic pattern of the N-Ru stretching band of $NRuF_3$ caused by the seven naturally occurring, stable ruthenium isotopes.

Isotopologue	ν(NRu), a'	ν(FRuF) antisym., a''	ν(RuF'), a'
$N^{96}RuF_3$	1109.57	673.32	637.93
$N^{98}RuF_3$	1108.25	*too weak*	*too weak*
$N^{99}RuF_3$	1107.46	670.80	636.83
$N^{100}RuF_3$	1106.83	669.96	636.43
$N^{101}RuF_3$	1105.97	669.12	636.05
$N^{102}RuF_3$	1105.40	668.48	635.84
$N^{104}RuF_3$	1104.10	666.94	635.16

Table 6. The experimental isotopic pattern of the N-Ru stretching band of $NRuF_4$ caused by the seven naturally occurring, stable ruthenium isotopes.

Isotopologue	ν(NRu), a'
$N^{96}RuF_4$	705.85
$N^{98}RuF_4$	703.73
$N^{99}RuF_4$	702.87
$N^{100}RuF_4$	701.89
$N^{101}RuF_4$	700.89
$N^{102}RuF_4$	700.01
$N^{104}RuF_4$	698.25

Table S7. Comparison of the experimental and calculated $^{96/104}Ru$ isotopic ratios of the bands assigned to $NRuF_3$ and $NRuF_4$.

	Frequency [cm^{-1}] [a]	$^{96/104}$Ru Isotopic Ratio		
	Exp.	Exp.	B3LYP	CCSD(T)
$NRuF_3$				
ν(NRu), a’	1105.40	1.00495	1.00495	1.00497
ν(FRuF),antisym.a’’	668.48	1.00956	1.00940	1.00963
ν(RuF’),a’	635.84	1.00361	1.00414	1.00378
$NRuF_4$				
ν(NRu), a’	1098.53	- [b]	1.00509	1.00507
ν(NRu), a’	700.01	1.01088	1.01097	1.01093
Unknown Product	606.52	1.00289	-	-

[a] Band position of isotopologue containing the most abundant ruthenium isotope ^{102}Ru; [b] Intensity too low to safely assign the $NRu^{96}F_4$ band (see Figure S5)

Table S8. CCSD(T) diagnostics to estimate the presence of multi-reference character of NMF_3 (M = Fe, Ru, Os).

Compound	T_1	D_1	%TAE
$NFeF_3$ ($^3A_2$)	0.1013	0.4353	14.2
$NRuF_3$ ($^1A'$)	0.0356	0.1166	7.7
$NRuF_3$ ($^3A''$)	0.0296	0.0922	7.8
$NOsF_3$ ($^1A'$)	0.0280	0.0915	6.8
$NOsF_3$ ($^3A''$)	0.0290	0.0839	6.6

Table S9. AIM charges, NPA charges, NLMO bond orders (totals by atom) and Wiberg bond indices (totals by atom) for all experimentally detected NMF_3 and NMF_4 species calculated at the DFT (M06-L for M = Fe and B3LYP for M = Ru, Os) levels of theory.

	$NFeF_3$ ($^3A_2$)		$NRuF_3$ ($^1A_1$)		$NOsF_3$ ($^1A'$)		$NOsF_3$ ($^3A''$)		$NRuF_4$ ($^2B_2$)		$NOsF_4$ ($^2B_2$)	
	**N**	**Fe**	**N**	**Ru**	**N**	**Os**	**N**	**Os**	**N**	**Ru**	**N**	**Os**
	**M06-L**		**B3LYP**									
AIM Charge	−0.25	1.90	−0.35	2.04	−0.49	2.21	−0.51	2.25	−0.25	2.38	−0.40	2.66
NPA Charge	0.05	1.41	−0.03	1.55	−0.21	1.72	−0.26	1.80	0.08	1.69	−0.11	1.99
NLMO B.O.	2.30	3.21	2.62	3.64	2.77	3.84	2.80	3.80	2.28	4.30	2.54	4.48
Wiberg B.I.	2.52	3.29	3.04	4.28	3.03	4.47	2.86	3.98	3.05	4.61	3.04	4.76

Table S10. Electronic energies (hartree) and energy differences (kJ mol^{-1}) of singlet and triplet $NRuF_3$ calculated at the ROHF/UCCSD(T) level of theory with basis sets up to quintuple zeta quality, as well as CBS extrapolations. The single point energies were calculated at the minima obtained at the CCSD(T)/aug-cc-pVTZ(-PP) levels.

Basis set	NRuF$_3$ (C_s, ^{1}A')			NRuF$_3$ (C_{3v}, $^3A_2$)			ΔE_{T-S}
	Reference E.	Correlation E.	Total Energy	Reference E.	Correlation E.	Total Energy	
aVDZ(-PP)	−446.504523	−1.181185	−447.685709	−446.507003	−1.181062	−447.688064	−6.2
aVTZ(-PP)	−446.600941	−1.422827	−448.023769	−446.602085	−1.420950	−448.023035	1.9
aQZ(-PP)	−446.627712	−1.506839	−448.134551	−446.628722	−1.504153	−448.132875	4.4
aV5Z(-PP)	−446.634844	−1.538137	−448.172981	−446.635786	−1.534972	−448.170758	5.8
CBS [a]	−446.634844	−1.570975	−448.205819	−446.635786	−1.567306	−448.203092	7.2
CBS [b]	−445.634844	−1.556720	−447.191564	−445.635786	−1.553102	−447.188889	7.0
apwCVDZ(−PP)	−446.511415	−1.500142	−448.011557	−446.513189	−1.501410	−448.014598	−8.0
apwCVTZ(−PP)	−446.603800	−1.818958	−448.422758	−446.604814	−1.818014	−448.422827	−0.2
apwCVQZ(−PP)	−446.628602	−1.928322	−448.556924	−446.629599	−1.926530	−448.556129	2.1
apwCV5Z(−PP)	−446.635065	−1.970985	−448.606050	−446.635995	−1.968682	−448.604677	3.6
CBS [a]	−446.635065	−2.015746	−448.650811	−446.635995	−2.012907	−448.648902	5.0
CBS [b]	−445.635065	−1.998272	−447.633337	−445.635995	−1.995455	−447.631450	5.0

[a] Two-point extrapolation of the QZ and 5Z CCSD(T) correlation energies via $E_n = E_{\mathrm{CBS}} + \frac{A}{(n+1)^3}$, where *n* is the cardinal number of the basis set (4 = QZ and 5 = 5Z) and *A* is a fitting parameter.[13b,45] [b] Exponential three point extrapolation of the TZ, QZ and 5Z CCSD(T) correlation energies via $E_n = E_{CBS} + Be^{-\alpha n}$, where *n* is the cardinal number of the basis set (3 = TZ, 4 = QZ and 5 = 5Z), and α and *B* are fitting parameters.[46]

Table S11. Electronic energies (hartree) and energy differences (kJ mol^{-1}) of singlet and triplet $NOsF_3$ calculated at the ROHF/ UCCSD(T) level of theory with basis sets up to quintuple zeta quality, as well as CBS extrapolations. The single point energies were calculated at the minima obtained at the CCSD(T)/aug−cc−pVTZ(−PP) level of theory.

Basis set	**$NOsF_3$ (C_s, ^{1}A')**			**$NOsF_3$ (C_s, ^{3}A'')**			ΔE_{T-S}
	**Reference E.**	**Correlation E.**	**Total Energy**	**Reference E.**	**Correlation E.**	**Total Energy**	
aVDZ(−PP)	−442.857904	−1.147078	−444.004982	−442.871032	−1.137832	−444.008864	−10.2
aVTZ(−PP)	−442.957181	−1.384106	−444.341287	−442.969238	−1.373362	−444.342600	−3.4
aVQZ(−PP)	−442.983445	−1.466771	−444.450217	−442.995504	−1.455350	−444.450854	−1.7
aV5Z(−PP)	−442.990207	−1.497605	−444.487811	−443.002211	−1.485787	−444.487998	−0.5
CBS [a]	−442.990207	−1.529955	−444.520161	−443.002211	−1.517720	−444.519931	0.6
CBS [b]	−442.990207	−1.515947	−444.506153	−443.002211	−1.503757	−444.505968	0.5
awCVDZ(−PP)	−442.864299	−1.406781	−444.271080	−442.877117	−1.398780	−444.275898	−12.6
apwCVTZ(−PP)	−442.959193	−1.726200	−444.685394	−442.971236	−1.716295	−444.687530	−5.6
apwCVQZ(−PP)	−442.983945	−1.834221	−444.818166	−442.995976	−1.823596	−444.819572	−3.7
apwCV5Z(−PP)	−442.990330	−1.876415	−444.866746	−443.002320	−1.865356	−444.867676	−2.4
CBS [a]	−442.990330	−1.920685	−444.911016	−443.002320	−1.909169	−444.911490	−1.2
CBS [b]	−441.990330	−1.903463	−443.893793	−442.002320	−1.891963	−443.894284	−1.3

[a] Two-point extrapolation of the QZ and 5Z CCSD(T) correlation energies via $E_n = E_{\mathrm{CBS}} + \frac{A}{(n+1)^3}$, where n is the cardinal number of the basis set (4 = QZ and 5 = 5Z) and A is a fitting parameter.[13a,45]; [b] Exponential three point extrapolation of the TZ, QZ and 5Z CCSD(T) correlation energies via $E_n = E_{CBS} + Be^{-\alpha n}$, where n is the cardinal number of the basis set (3 = TZ, 4 = QZ and 5 = 5Z), and α and B are fitting parameters.[46]

Table S12. Total electronic energies (E_{el}) in hartree and electronic energy differences (ΔE_{el}) in kJ mol^{-1} of different points at the ^{1}A' and ^{3}A" energy potential surfaces (PES) of $NOsF_3$ obtained at the B3LYP/def2-QZVP and UCCSD(T)/cc-pVTZ(-PP) level of theory.

	B3LYP/def2-QZVP		CCSD(T)/cc-pVTZ(-PP)	
	E_{el} [hartree]	ΔE_{el} [kJ mol^{-1}]	E_{el} [hartree]	ΔE_{el} [kJ mol^{-1}]
^{1}A' Minimum	−445.014761	0	-444.559950	0
^{1}A' MECP	−445.009614	13.5	-444.569336	24.6
^{3}A" Minimum	−445.018544	0	-444.559951	0
^{3}A" MECP	−445.009649	23.4	-444.568806	23.2

Table S13. AIM charges, NPA charges, NLMO bond orders (totals by atom) and Wiberg bond indices (totals by atom) for NMF_2 species calculated the B3LYP (M = Ru, Os) and M06-L (M = Fe) levels of theory.

	$NFeF_2$ ($^2A_2$)		$NRuF_2$ ($^2A_2$)		$NOsF_2$ ($^2A_2$)	
	N	Fe	N	Ru	N	Os
AIM Charge	−0.35	1.53	−0.40	1.62	−0.50	1.72
NPA Charge	−0.03	1.10	−0.12	1.25	−0.22	1.28
NLMO B.O.	2.62	3.30	2.82	3.40	2.63	3.49
Wiberg B.I.	2.79	3.32	3.02	3.61	3.00	3.80

Table S14. Adiabatic Potential Energy Surfaces of $NFeF_3$ (Figure 6**a**) obtained at the SA-CASSCF(8,8)/NEVPT2/cc-pVTZ-DK,Fe=cc-pwCVTZ-DK level. RMN, RMF1, A1, RMF2, A2 and D1 are the MN bond length, MF’ bond length, the NMF’ bond angle, the MF bond length, the NMF angle and the F’MNF dihedral angle (see Figure 5). Energies in hartree, bond lengths in Å and angles in degree.

RMN	RMF1	A1	RMF2	A2	D1	a^1A'	b^1A'	c^1A'	a^1A''	b^1A''	a^3A'	b^3A'	a^3A''	b^3A''
1.51107	1.68807	96.60307	1.69638	113.74074	93.66276	-1626.076795	-1625.973598	-1625.975589	-1626.031556	-1626.001221	-1626.004367	-1625.976403	-1626.073821	-1626.024558
1.51269	1.69049	97.44400	1.69821	113.35754	95.54399	-1626.078618	-1625.978474	-1625.983043	-1626.036292	-1626.004008	-1626.008436	-1625.978687	-1626.079081	-1626.027246
1.51430	1.69291	98.28493	1.70003	112.97435	97.42522	-1626.079862	-1625.984452	-1625.988535	-1626.040546	-1626.006392	-1626.012018	-1625.980464	-1626.083914	-1626.029461
1.51592	1.69533	99.12586	1.70186	112.59116	99.30645	-1626.080560	-1625.991012	-1625.992605	-1626.044352	-1626.008383	-1626.015114	-1625.981835	-1626.088341	-1626.031225
1.51753	1.69775	99.96678	1.70368	112.20797	101.18768	-1626.080702	-1625.997867	-1625.995653	-1626.047761	-1626.009997	-1626.017722	-1625.982965	-1626.092401	-1626.032555
1.51915	1.70017	100.80771	1.70551	111.82478	103.06891	-1626.080297	-1626.004749	-1625.997961	-1626.050816	-1626.011264	-1626.019926	-1625.984398	-1626.096105	-1626.033523
1.52076	1.70259	101.64864	1.70733	111.44159	104.95015	-1626.079392	-1626.011629	-1625.999712	-1626.053544	-1626.012290	-1626.021798	-1625.987494	-1626.099465	-1626.034236
1.52237	1.70501	102.48956	1.70916	111.05840	106.83138	-1626.077985	-1626.018207	-1626.000773	-1626.055843	-1626.012819	-1626.023110	-1625.992245	-1626.102384	-1626.034436
1.52399	1.70742	103.33049	1.71099	110.67520	108.71261	-1626.076178	-1626.024287	-1626.001137	-1626.057719	-1626.012711	-1626.023783	-1625.996882	-1626.104874	-1626.034000
1.52560	1.70984	104.17142	1.71281	110.29201	110.59384	-1626.074017	-1626.029955	-1626.000939	-1626.059241	-1626.012092	-1626.023940	-1626.001113	-1626.106989	-1626.033062
1.52722	1.71226	105.01235	1.71464	109.90882	112.47507	-1626.071530	-1626.035239	-1626.000206	-1626.060441	-1626.011023	-1626.023614	-1626.004951	-1626.108748	-1626.031693
1.52883	1.71468	105.85327	1.71646	109.52563	114.35630	-1626.068803	-1626.040051	-1625.998972	-1626.061324	-1626.009548	-1626.022866	-1626.008361	-1626.110148	-1626.029937
1.53045	1.71710	106.69420	1.71829	109.14244	116.23754	-1626.066001	-1626.044234	-1625.997225	-1626.061897	-1626.007743	-1626.021783	-1626.011267	-1626.111187	-1626.027851
1.53206	1.71952	107.53513	1.72011	108.75925	118.11877	-1626.063495	-1626.047453	-1625.994885	-1626.062172	-1626.005836	-1626.020568	-1626.013504	-1626.111872	-1626.025615
1.53367	1.72194	108.37606	1.72194	108.37606	120.00000	-1626.063184	-1626.048127	-1625.993172	-1626.062202	-1626.005314	-1626.019965	-1626.014059	-1626.112008	-1626.025016
1.53180	1.72030	109.58665	1.71943	107.88987	121.21794	-1626.063184	-1626.048127	-1625.993172	-1626.062202	-1626.005314	-1626.019965	-1626.014059	-1626.112008	-1626.025016
1.52992	1.71866	110.79725	1.71692	107.40368	122.43588	-1626.064964	-1626.046198	-1625.994284	-1626.062023	-1626.006522	-1626.020447	-1626.012793	-1626.111608	-1626.026343
1.52804	1.71701	112.00784	1.71441	106.91749	123.65382	-1626.067020	-1626.043548	-1625.995043	-1626.061621	-1626.007799	-1626.020925	-1626.011076	-1626.110977	-1626.027648
1.52617	1.71537	113.21843	1.71190	106.43130	124.87176	-1626.069094	-1626.040372	-1625.995496	-1626.060971	-1626.008905	-1626.021288	-1626.008945	-1626.110096	-1626.028748
1.52429	1.71373	114.42903	1.70939	105.94511	126.08970	-1626.071045	-1626.036766	-1625.995654	-1626.060051	-1626.009757	-1626.021450	-1626.006437	-1626.108939	-1626.029581
1.52241	1.71209	115.63962	1.70688	105.45892	127.30764	-1626.072790	-1626.032777	-1625.995507	-1626.058841	-1626.010306	-1626.021357	-1626.003568	-1626.107486	-1626.030108
1.52054	1.71045	116.85022	1.70437	104.97273	128.52558	-1626.074269	-1626.028424	-1625.995036	-1626.057315	-1626.010518	-1626.020968	-1626.000342	-1626.105713	-1626.030297
1.51866	1.70881	118.06081	1.70186	104.48654	129.74352	-1626.075427	-1626.023713	-1625.994221	-1626.055449	-1626.010353	-1626.020251	-1625.996756	-1626.103592	-1626.030111
1.51678	1.70717	119.27141	1.69936	104.00035	130.96146	-1626.076212	-1626.018620	-1625.993024	-1626.053208	-1626.009782	-1626.019170	-1625.992793	-1626.101090	-1626.029515
1.51491	1.70552	120.48200	1.69685	103.51416	132.17940	-1626.076600	-1626.013096	-1625.991389	-1626.050558	-1626.008762	-1626.017676	-1625.988424	-1626.098169	-1626.028465
1.51303	1.70388	121.69259	1.69434	103.02797	133.39734	-1626.076552	-1626.007069	-1625.989272	-1626.047435	-1626.007221	-1626.015744	-1625.983661	-1626.094758	-1626.026914
1.51115	1.70224	122.90319	1.69183	102.54178	134.61528	-1626.076028	-1626.000483	-1625.986583	-1626.043795	-1626.005066	-1626.013258	-1625.978619	-1626.090828	-1626.024733
1.50928	1.70060	124.11378	1.68932	102.05560	135.83322	-1626.074946	-1625.993227	-1625.983227	-1626.039529	-1626.002198	-1626.010153	-1625.973712	-1626.086257	-1626.021843

1.50740	1.69896	125.32438	1.68681	101.56941	137.05116	-1626.073190	-1625.985329	-1625.979147	-1626.034567	-1625.998621	-1626.006411	-1625.969433	-1626.080964	-1626.018246

Table S15. Adiabatic Potential Energy Surfaces of $NRuF_3$ (Figure 6**b**) obtained at the SA-CASSCF(8,8)/NEVPT2/cc-pVTZ-DK level. RMN, RMF1, A1, RMF2, A2 and D1 are the RuN bond length, the RuF’ bond length, the NRuF’ bond angle, the RuF bond length, the NRuF angle and the F’RuNF dihedral angle (see Figure 5). Energies in hartree, bond lengths in Å and angles in degree.

RMN	RMF1	A1	RMF2	A2	D1	*a*^{1}A’	*b*^{1}A’	*a*^{1}A“	*a*^{3}A’	*b*^{3}A’	*a*^{3}A“	*b*^{3}A“	*c*^{3}A“
1.59151	1.85257	95.41620	1.86341	115.28640	88.32920	-4882.060830	-4881.919104	-4882.001768	-4881.946690	-4881.904183	-4882.025083	-4881.978223	-4881.898661
1.59184	1.85406	96.74090	1.86412	115.19180	90.59140	-4882.063281	-4881.927828	-4882.007362	-4881.950836	-4881.907225	-4882.030515	-4881.980059	-4881.902092
1.59217	1.85554	98.06560	1.86483	115.09720	92.85360	-4882.064930	-4881.936563	-4882.012431	-4881.954416	-4881.910018	-4882.035459	-4881.981343	-4881.904791
1.59250	1.85703	99.39030	1.86554	115.00260	95.11580	-4882.065785	-4881.945321	-4882.017006	-4881.957554	-4881.913016	-4882.039932	-4881.982242	-4881.906766
1.59283	1.85851	100.71500	1.86625	114.90800	97.37800	-4882.065948	-4881.954229	-4882.021143	-4881.960331	-4881.917505	-4882.043993	-4881.982894	-4881.908147
1.59316	1.86000	102.03970	1.86696	114.81340	99.64020	-4882.065243	-4881.962909	-4882.024801	-4881.962711	-4881.923836	-4882.047585	-4881.983073	-4881.909153
1.59349	1.86148	103.36440	1.86767	114.71880	101.90240	-4882.063576	-4881.971538	-4882.027830	-4881.965039	-4881.930240	-4882.050568	-4881.982740	-4881.912829
1.59382	1.86297	104.68910	1.86838	114.62420	104.16460	-4882.061164	-4881.979752	-4882.030308	-4881.965973	-4881.935116	-4882.053056	-4881.981220	-4881.919763
1.59415	1.86445	106.01380	1.86909	114.52960	106.42680	-4882.058350	-4881.987697	-4882.032477	-4881.966045	-4881.939294	-4882.055282	-4881.979006	-4881.926101
1.59448	1.86594	107.33850	1.86981	114.43500	108.68900	-4882.055075	-4881.995371	-4882.034292	-4881.965532	-4881.943044	-4882.057184	-4881.976239	-4881.932024
1.59481	1.86742	108.66320	1.87052	114.34040	110.95120	-4882.051400	-4882.002705	-4882.035754	-4881.964493	-4881.946352	-4882.058757	-4881.972942	-4881.937513
1.59514	1.86891	109.98790	1.87123	114.24580	113.21340	-4882.047434	-4882.009588	-4882.036883	-4881.962960	-4881.949154	-4882.060016	-4881.969112	-4881.942530
1.59547	1.87039	111.31260	1.87194	114.15120	115.47560	-4882.043341	-4882.015751	-4882.037643	-4881.961051	-4881.951376	-4882.060916	-4881.964789	-4881.947065
1.59580	1.87188	112.63730	1.87265	114.05660	117.73780	-4882.039674	-4882.020575	-4882.038027	-4881.958945	-4881.952788	-4882.061446	-4881.960020	-4881.950950
1.59613	*1.87336*	*113.96200*	*1.87336*	*113.96200*	*120.00000*	*-4882.038031*	*-4882.022576*	*-4882.038031*	*-4881.957291*	*-4881.952738*	*-4882.061597*	*-4881.956156*	*-4881.952738*
1.59616	1.87272	115.15450	1.87203	113.00220	121.52860	-4882.039341	-4882.021286	-4882.037853	-4881.958248	-4881.953192	-4882.061513	-4881.959073	-4881.951226
1.59619	1.87209	116.34700	1.87071	112.04240	123.05720	-4882.042154	-4882.017919	-4882.037346	-4881.959443	-4881.952792	-4882.061118	-4881.962961	-4881.948019
1.59622	1.87145	117.53950	1.86938	111.08260	124.58580	-4882.045400	-4882.013381	-4882.036498	-4881.960665	-4881.951713	-4882.060398	-4881.966539	-4881.944360
1.59625	1.87082	118.73200	1.86805	110.12280	126.11440	-4882.048579	-4882.008109	-4882.035288	-4881.961729	-4881.950112	-4882.059325	-4881.969743	-4881.940369
1.59629	1.87018	119.92450	1.86673	109.16300	127.64300	-4882.051534	-4882.002324	-4882.033737	-4881.962509	-4881.948018	-4882.057921	-4881.972504	-4881.936048
1.59632	1.86954	121.11700	1.86540	108.20320	129.17160	-4882.054152	-4881.996096	-4882.031811	-4881.962954	-4881.945474	-4882.056146	-4881.974817	-4881.931403
1.59635	1.86891	122.30950	1.86407	107.24340	130.70020	-4882.056359	-4881.989462	-4882.029484	-4881.963027	-4881.942479	-4882.053972	-4881.976643	-4881.926432
1.59638	1.86827	123.50200	1.86274	106.28360	132.22880	-4882.058091	-4881.982418	-4882.026722	-4881.962686	-4881.939021	-4882.051360	-4881.977949	-4881.921111
1.59641	1.86764	124.69450	1.86142	105.32380	133.75740	-4882.059291	-4881.974942	-4882.023485	-4881.961883	-4881.935066	-4882.048268	-4881.978682	-4881.915395
1.59644	1.86700	125.88700	1.86009	104.36400	135.28600	-4882.059890	-4881.967004	-4882.019724	-4881.960558	-4881.930556	-4882.044647	-4881.978771	-4881.909206
1.59647	1.86636	127.07950	1.85876	103.40420	136.81460	-4882.060206	-4881.958638	-4882.015695	-4881.956846	-4881.924397	-4882.040850	-4881.977388	-4881.901885

1.59650	1.86573	128.27200	1.85744	102.44440	138.34320	-4882.059239	-4881.949526	-4882.010592	-4881.954053	-4881.918519	-4882.035860	-4881.975800	-4881.899549
1.59653	1.86509	129.46450	1.85611	101.48460	139.87180	-4882.057316	-4881.939663	-4882.004643	-4881.950406	-4881.911835	-4882.030006	-4881.973208	-4881.896354
1.59656	1.86446	130.65700	1.85478	100.52480	141.40040	-4882.054242	-4881.928833	-4881.997654	-4881.945699	-4881.904182	-4882.023089	-4881.969400	-4881.892112

Table S16. Adiabatic Potential Energy Surfaces of $NOsF_3$ (Figure 6**c**) obtained at the SA-CASSCF(8,8)/NEVPT2/cc-pVTZ-DK level. RMN, RMF1, A1, RMF2, A2 and D1 are the OsN bond length, the OsF' bond length, the NOsF' bond angle, the OsF bond length, the NOsF angle and the F'OsNF dihedral angle (see Figure 5). Energies in hartree, bond lengths in Å and angles in degree.

**RMN**	**RMF1**	**A1**	**RMF2**	**A2**	**D1**	***a*^{1}A'**	***b*^{1}A'**	***a*^{1}A"**	***a*^{3}A'**	***b*^{3}A'**	***a*^{3}A"**	***b*^{3}A"**	***c*^{3}A"**
1.62159	1.86334	96.09113	1.88474	116.01434	87.62533	-17592.580260	-17592.449170	-17592.529650	-17592.449780	-17592.417240	-17592.554670	-17592.486310	-17592.413990
1.62198	1.86548	97.45957	1.88536	115.95969	89.93780	-17592.582700	-17592.457310	-17592.534790	-17592.453820	-17592.420870	-17592.559420	-17592.488040	-17592.417530
1.62237	1.86763	98.82800	1.88597	115.90504	92.25028	-17592.584250	-17592.465260	-17592.539280	-17592.457280	-17592.424210	-17592.563530	-17592.489150	-17592.420110
1.62276	1.86977	100.19644	1.88659	115.85039	94.56276	-17592.585000	-17592.473110	-17592.543190	-17592.460220	-17592.427650	-17592.567070	-17592.489740	-17592.421870
1.62315	1.87192	101.56487	1.88721	115.79574	96.87523	-17592.584910	-17592.480770	-17592.546470	-17592.462620	-17592.431470	-17592.570030	-17592.489750	-17592.422830
1.62354	1.87406	102.93331	1.88782	115.74108	99.18771	-17592.584080	-17592.488190	-17592.549220	-17592.464530	-17592.435580	-17592.572480	-17592.489130	-17592.423240
1.62393	1.87621	104.30174	1.88844	115.68643	101.50019	-17592.582430	-17592.495450	-17592.551460	-17592.465920	-17592.439490	-17592.574480	-17592.487830	-17592.423430
1.62432	1.87835	105.67018	1.88905	115.63178	103.81266	-17592.579870	-17592.502660	-17592.553130	-17592.467470	-17592.443470	-17592.575970	-17592.486110	-17592.426950
1.62471	1.88050	107.03862	1.88967	115.57713	106.12514	-17592.576830	-17592.509450	-17592.554340	-17592.467540	-17592.446370	-17592.577080	-17592.483350	-17592.431900
1.62510	1.88264	108.40705	1.89029	115.52248	108.43762	-17592.573390	-17592.516010	-17592.555250	-17592.466890	-17592.448770	-17592.577950	-17592.479940	-17592.436490
1.62548	1.88479	109.77549	1.89090	115.46783	110.75009	-17592.569640	-17592.522400	-17592.555930	-17592.465800	-17592.450890	-17592.578670	-17592.476070	-17592.440890
1.62587	1.88693	111.14392	1.89152	115.41318	113.06257	-17592.565580	-17592.528480	-17592.556340	-17592.464240	-17592.452620	-17592.579180	-17592.471640	-17592.445000
1.62626	1.88908	112.51236	1.89213	115.35853	115.37505	-17592.561460	-17592.534090	-17592.556540	-17592.462330	-17592.453950	-17592.579550	-17592.466750	-17592.448830
1.62665	1.89122	113.88079	1.89275	115.30388	117.68752	-17592.557830	-17592.538600	-17592.556500	-17592.460230	-17592.454630	-17592.579730	-17592.461340	-17592.452290
1.62704	1.89337	115.24923	1.89337	115.24923	120.00000	-17592.556225	-17592.540465	-17592.556225	-17592.458417	-17592.454196	-17592.579724	-17592.456442	-17592.454196
1.62701	1.89019	115.49010	1.89204	114.22360	121.90130	-17592.557870	-17592.539180	-17592.556030	-17592.460830	-17592.456170	-17592.579760	-17592.461280	-17592.453320
1.62699	1.88702	115.73098	1.89072	113.19798	123.80260	-17592.561240	-17592.535720	-17592.555580	-17592.463620	-17592.457110	-17592.579600	-17592.466790	-17592.450920
1.62696	1.88384	115.97186	1.88939	112.17236	125.70390	-17592.565200	-17592.530960	-17592.554810	-17592.466440	-17592.457210	-17592.579160	-17592.471930	-17592.448000
1.62694	1.88067	116.21274	1.88807	111.14674	127.60520	-17592.569270	-17592.525430	-17592.553770	-17592.469040	-17592.456690	-17592.578470	-17592.476700	-17592.444620
1.62691	1.87749	116.45361	1.88674	110.12111	129.50650	-17592.573230	-17592.519290	-17592.552400	-17592.471300	-17592.455610	-17592.577490	-17592.481060	-17592.440770
1.62688	1.87432	116.69449	1.88542	109.09549	131.40780	-17592.576930	-17592.512540	-17592.550640	-17592.473060	-17592.453910	-17592.576130	-17592.484920	-17592.436360
1.62686	1.87114	116.93537	1.88409	108.06987	133.30910	-17592.580230	-17592.505170	-17592.548400	-17592.474250	-17592.451530	-17592.574280	-17592.488190	-17592.431350
1.62683	1.86797	117.17625	1.88277	107.04425	135.21040	-17592.582950	-17592.497110	-17592.545540	-17592.474700	-17592.448370	-17592.571810	-17592.490690	-17592.425630
1.62681	1.86479	117.41712	1.88144	106.01862	137.11170	-17592.584940	-17592.488290	-17592.541950	-17592.474270	-17592.444290	-17592.568570	-17592.492270	-17592.419080

1.62678	1.86162	117.65800	1.88012	104.99300	139.01300	-17592.586340	-17592.478320	-17592.537570	-17592.471120	-17592.438100	-17592.564610	-17592.492080	-17592.414940
1.62675	1.85845	117.89888	1.87880	103.96738	140.91430	-17592.585880	-17592.466980	-17592.531570	-17592.467980	-17592.431460	-17592.558900	-17592.490900	-17592.412790
1.62673	1.85527	118.13975	1.87747	102.94175	142.81560	-17592.583560	-17592.453830	-17592.523750	-17592.462960	-17592.423070	-17592.551350	-17592.487710	-17592.408990
1.62670	1.85210	118.38063	1.87615	101.91613	144.71690	-17592.578580	-17592.438120	-17592.513360	-17592.455300	-17592.412390	-17592.541150	-17592.481690	-17592.402700
1.62668	1.84892	118.62151	1.87482	100.89051	146.61820	-17592.570020	-17592.418830	-17592.499410	-17592.444100	-17592.399110	-17592.527350	-17592.471860	-17592.392960

Table S17. Adiabatic Potential Energy Surfaces of $NOsF_3$ (Figure 6**d**) obtained at the SA-CASSCF(8,8)/NEVPT2/cc-pVTZ-DK level. RMN, RMF1, A1, RMF2, A2 and D1 are the OsN bond length, the OsF’ bond length, the NOsF’ bond angle, the OsF bond length, the NOsF angle and the F’OsNF dihedral angle (see Figure 5). Energies in hartree, bond lengths in Å and angles in degree.

RMN	RMF1	A1	RMF2	A2	D1	*a*^{1}A’	*b*^{1}A’	*a*^{1}A“	*a*^{3}A’	*b*^{3}A’	*a*^{3}A“	*b*^{3}A“	*c*^{3}A“
1.64110	1.89256	126.92771	1.86221	101.49391	93.35660	-17592.559350	-17592.525640	-17592.559190	-17592.471240	-17592.438640	-17592.583180	-17592.468440	-17592.450060
1.64009	1.89262	126.09353	1.86443	102.47643	95.25970	-17592.561810	-17592.527380	-17592.561130	-17592.473220	-17592.439580	-17592.584840	-17592.470890	-17592.451570
1.63909	1.89267	125.25935	1.86666	103.45895	97.16280	-17592.563550	-17592.528950	-17592.562500	-17592.474700	-17592.440770	-17592.585940	-17592.472820	-17592.453000
1.63808	1.89273	124.42518	1.86888	104.44148	99.06590	-17592.564670	-17592.530360	-17592.563340	-17592.475760	-17592.443380	-17592.586520	-17592.474360	-17592.454430
1.63708	1.89279	123.59100	1.87111	105.42400	100.96900	-17592.565210	-17592.531570	-17592.563670	-17592.476250	-17592.446500	-17592.586620	-17592.475240	-17592.455550
1.63608	1.89285	122.75682	1.87334	106.40652	102.87210	-17592.565150	-17592.532530	-17592.563490	-17592.476020	-17592.448600	-17592.586220	-17592.475280	-17592.456190
1.63507	1.89291	121.92265	1.87556	107.38905	104.77520	-17592.564620	-17592.533390	-17592.562930	-17592.475230	-17592.450090	-17592.585470	-17592.474640	-17592.456530
1.63407	1.89296	121.08847	1.87779	108.37157	106.67830	-17592.563790	-17592.534170	-17592.562140	-17592.474020	-17592.451170	-17592.584540	-17592.473470	-17592.456610
1.63306	1.89302	120.25429	1.88001	109.35409	108.58140	-17592.562710	-17592.534890	-17592.561130	-17592.472380	-17592.451980	-17592.583460	-17592.471800	-17592.456480
1.63206	1.89308	119.42011	1.88224	110.33661	110.48450	-17592.561490	-17592.535780	-17592.560100	-17592.470460	-17592.452730	-17592.582440	-17592.469800	-17592.456310
1.63106	1.89314	118.58594	1.88446	111.31914	112.38760	-17592.560100	-17592.536780	-17592.559060	-17592.468220	-17592.453370	-17592.581480	-17592.467410	-17592.456080
1.63005	1.89319	117.75176	1.88669	112.30166	114.29070	-17592.558720	-17592.537980	-17592.558160	-17592.465780	-17592.453990	-17592.580750	-17592.464760	-17592.455880
1.62905	1.89325	116.91758	1.88891	113.28418	116.19380	-17592.557410	-17592.539170	-17592.557360	-17592.463140	-17592.454480	-17592.580200	-17592.461850	-17592.455650
1.62804	1.89331	116.08340	1.89114	114.26670	118.09690	-17592.556500	-17592.540170	-17592.556760	-17592.460550	-17592.454770	-17592.579910	-17592.458850	-17592.455360
1.62704	1.89337	115.24923	1.89337	115.24923	120.00000	-17592.556225	-17592.540465	-17592.556225	-17592.458417	-17592.454196	-17592.579724	-17592.456442	-17592.454196
1.62701	1.89019	115.49010	1.89204	114.22360	121.90130	-17592.557870	-17592.539180	-17592.556030	-17592.460830	-17592.456170	-17592.579760	-17592.461280	-17592.453320
1.62699	1.88702	115.73098	1.89072	113.19798	123.80260	-17592.561240	-17592.535720	-17592.555580	-17592.463620	-17592.457110	-17592.579600	-17592.466790	-17592.450920
1.62696	1.88384	115.97186	1.88939	112.17236	125.70390	-17592.565200	-17592.530960	-17592.554810	-17592.466440	-17592.457210	-17592.579160	-17592.471930	-17592.448000
1.62694	1.88067	116.21274	1.88807	111.14674	127.60520	-17592.569270	-17592.525430	-17592.553770	-17592.469040	-17592.456690	-17592.578470	-17592.476700	-17592.444620
1.62691	1.87749	116.45361	1.88674	110.12111	129.50650	-17592.573230	-17592.519290	-17592.552400	-17592.471300	-17592.455610	-17592.577490	-17592.481060	-17592.440770
1.62688	1.87432	116.69449	1.88542	109.09549	131.40780	-17592.576930	-17592.512540	-17592.550640	-17592.473060	-17592.453910	-17592.576130	-17592.484920	-17592.436360
1.62686	1.87114	116.93537	1.88409	108.06987	133.30910	-17592.580230	-17592.505170	-17592.548400	-17592.474250	-17592.451530	-17592.574280	-17592.488190	-17592.431350

1.62683	1.86797	117.17625	1.88277	107.04425	135.21040	-17592.582950	-17592.497110	-17592.545540	-17592.474700	-17592.448370	-17592.571810	-17592.490690	-17592.425630
1.62681	1.86479	117.41712	1.88144	106.01862	137.11170	-17592.584940	-17592.488290	-17592.541950	-17592.474270	-17592.444290	-17592.568570	-17592.492270	-17592.419080
1.62678	1.86162	117.65800	1.88012	104.99300	139.01300	-17592.586340	-17592.478320	-17592.537570	-17592.471120	-17592.438100	-17592.564610	-17592.492080	-17592.414940
1.62675	1.85845	117.89888	1.87880	103.96738	140.91430	-17592.585880	-17592.466980	-17592.531570	-17592.467980	-17592.431460	-17592.558900	-17592.490900	-17592.412790
1.62673	1.85527	118.13975	1.87747	102.94175	142.81560	-17592.583560	-17592.453830	-17592.523750	-17592.462960	-17592.423070	-17592.551350	-17592.487710	-17592.408990
1.62670	1.85210	118.38063	1.87615	101.91613	144.71690	-17592.578580	-17592.438120	-17592.513360	-17592.455300	-17592.412390	-17592.541150	-17592.481690	-17592.402700
1.62668	1.84892	118.62151	1.87482	100.89051	146.61820	-17592.570020	-17592.418830	-17592.499410	-17592.444100	-17592.399110	-17592.527350	-17592.471860	-17592.392960

Calculated molecular structures and vibrational data

The following table list computed z-matrices (structures), total energies, and vibrational data of optimized structures in C_s point group symmetry (unless stated otherwise) and with positive HOMO-LUMO (closed shell) or SOMO-LUMO (open shell) gap.

**F_2NFeF ($^3A'$)**	
B3LYP/def2-QZVP	
Cartesian coordinates	5<br>Energy = -1617.885680869<br>N 0.0200174 -0.7334605 0.0000000<br>F -0.9471905 -1.7059422 0.0000000<br>F 1.1888999 -1.4511922 0.0000000<br>Fe -0.0918882 1.0591772 0.0000000<br>F -0.1698386 2.8314177 0.0000000
Vibrational data (^{14}N)	# mode symmetry wave number IR intensity selection rules<br># cm**(-1) km/mol IR RAMAN<br>7 a" 29.43 15.41477 YES YES<br>8 a' 96.55 15.17501 YES YES<br>9 a' 210.30 18.55117 YES YES<br>10 a" 255.91 5.75702 YES YES<br>11 a' 329.10 0.32272 YES YES<br>12 a' 565.22 1.26244 YES YES<br>13 a' 673.12 164.84817 YES YES<br>14 a' 874.71 155.17964 YES YES<br>15 a' 1034.82 301.48996 YES YES

<table>
<tr><td colspan="2">BP86/def2-QZVP</td></tr>
<tr><td>Cartesian coordinates</td><td><pre>5
Energy = -1618.346313239
N   0.0373682   -0.6538728   0.0000000
F   -0.9620967   -1.6760250   0.0000000
F   1.1954484   -1.4730200   0.0000000
Fe   -0.0814740   1.0256217   0.0000000
F   -0.1892459   2.7772961   0.0000000</pre></td></tr>
<tr><td>Vibrational data (14N)</td><td><pre># mode   symmetry   wave number   IR intensity   selection rules
cm**(-1)   km/mol   IR   RAMAN
     7   a"   37.00   12.39328   YES   YES
     8   a'   95.16   4.31188   YES   YES
     9   a'   275.14   3.66558   YES   YES
    10   a'   366.42   6.90198   YES   YES
    11   a"   376.58   6.09699   YES   YES
    12   a'   424.33   218.11955   YES   YES
    13   a'   547.90   6.99519   YES   YES
    14   a'   682.11   290.25040   YES   YES
    15   a'   841.43   184.03013   YES   YES</pre></td></tr>
<tr><td colspan="2">M06-L/def2-QZVP</td></tr>
<tr><td>Cartesian coordinates</td><td><pre>5
Energy = -1617.979179046
N   0.0337354   -0.6779314   0.0000000
F   -0.9400297   -1.6687692   0.0000000
F   1.1742336   -1.4749600   0.0000000
Fe   -0.0719128   1.0349806   0.0000000
F   -0.1960266   2.7866800   0.0000000</pre></td></tr>
<tr><td>Vibrational data (14N)</td><td><pre># mode   symmetry   wave number   IR intensity   selection rules
cm**(-1)   km/mol   IR   RAMAN
     7   a'   108.20   7.66625   YES   YES
     8   a"   195.66   15.13882   YES   YES</pre></td></tr>
</table>

	9 a' 296.83 8.38855 YES YES<br>10 a' 360.44 0.92347 YES YES<br>11 a" 380.05 6.25535 YES YES<br>12 a' 531.21 209.24993 YES YES<br>13 a' 583.00 10.87019 YES YES<br>14 a' 700.12 232.26580 YES YES<br>15 a' 906.00 387.39380 YES YES
**$FNFeF_2$ ($^3A''$)**	
B3LYP/def2-QZVP	
Cartesian coordinates	5<br>Energy = -1617.975573816<br>Fe 0.6712147 0.2301833 0.0000000<br>F 0.9771787 0.5558377 1.6952085<br>F 0.9771787 0.5558377 -1.6952085<br>N -0.7181742 -0.9790599 0.0000000<br>F -1.9073979 -0.3627989 0.0000000
Vibrational data (^{14}N)	# mode symmetry wave number IR intensity selection rules<br># cm**(-1) km/mol IR RAMAN<br>7 a" 73.65 0.03279 YES YES<br>8 a' 139.35 26.98295 YES YES<br>9 a' 147.12 24.98256 YES YES<br>10 a' 217.52 3.56907 YES YES<br>11 a" 223.66 0.34654 YES YES<br>12 a' 432.33 13.07446 YES YES<br>13 a' 614.25 67.29753 YES YES<br>14 a" 743.72 216.77241 YES YES<br>15 a' 1024.90 271.91027 YES YES
BP86/def2-QZVP	
Cartesian coordinates	5<br>Energy = -1618.440612378

	Fe 0.5963808 0.0784963 0.0000000<br>F 1.0303946 0.6221026 1.5915390<br>F 1.0303946 0.6221026 -1.5915390<br>N -0.6884254 -0.9300658 0.0000000<br>F -1.9687445 -0.3926356 0.0000000
Vibrational data (^{14}N)	# mode symmetry wave number IR intensity selection rules<br># cm**(-1) km/mol IR RAMAN<br>7 a" 107.16 0.30397 YES YES<br>8 a' 126.35 17.17554 YES YES<br>9 a' 140.31 14.06378 YES YES<br>10 a' 265.84 0.42271 YES YES<br>11 a" 329.58 0.01683 YES YES<br>12 a' 611.04 20.89920 YES YES<br>13 a' 696.69 158.89399 YES YES<br>14 a" 742.40 152.63765 YES YES<br>15 a' 841.16 191.76773 YES YES
M06-L/def2-QZVP	
Cartesian coordinates	5<br>Energy = -1618.080219914<br>N -0.6009009 -1.0148392 0.0000000<br>F 0.3518498 -1.9608345 0.0000000<br>Fe -0.1143908 0.6270698 0.0000000<br>F 0.1817210 1.1743019 -1.6278403<br>F 0.1817210 1.1743019 1.6278403
Vibrational data (^{14}N)	# mode symmetry wave number IR intensity selection rules<br># cm**(-1) km/mol IR RAMAN<br>7 a" 90.71 0.17636 YES YES<br>8 a' 128.27 13.60551 YES YES<br>9 a' 147.87 21.23656 YES YES<br>10 a' 274.73 4.08887 YES YES<br>11 a" 289.45 0.34049 YES YES

	12 a' 512.70 9.27683 YES YES<br>13 a' 651.82 73.46579 YES YES<br>14 a" 747.45 187.87762 YES YES<br>15 a' 959.46 328.98704 YES YES
Vibrational data (^{15}N)	# mode symmetry wave number IR intensity selection rules<br># cm**(-1) km/mol IR RAMAN<br>7 a" 90.64 0.17226 YES YES<br>8 a' 128.13 13.57629 YES YES<br>9 a' 147.84 21.22452 YES YES<br>10 a' 272.85 4.03667 YES YES<br>11 a" 282.25 0.35791 YES YES<br>12 a' 498.93 8.32778 YES YES<br>13 a' 651.02 74.65331 YES YES<br>14 a" 747.44 187.86737 YES YES<br>15 a' 941.70 313.72487 YES YES
**$NFeF_3$ (C_{3v} – $^3A_2$)**	
B3LYP/def2-QZVP	
Cartesian coordinates	5<br>Energy = -1617.976610363<br>Fe -0.0000000 0.0000000 0.0079618<br>N 0.0000000 0.0000000 -1.6302797<br>F 0.8342270 -1.4449235 0.5388982<br>F 0.8342270 1.4449235 0.5388982<br>F -1.6684539 0.0000000 0.5388982
Vibrational data (^{14}N)	# mode symmetry wave number IR intensity selection rules<br># cm**(-1) km/mol IR RAMAN<br>7 e 155.22 5.27661 YES YES<br>8 e 155.22 5.27661 YES YES<br>9 a1 187.18 8.83064 YES YES<br>10 e 232.67 3.91819 YES YES<br>11 e 232.67 3.91819 YES YES

	12 a1 421.35 21.22831 YES YES<br>13 a1 682.58 33.50537 YES YES<br>14 e 701.25 146.02441 YES YES<br>15 e 701.25 146.02441 YES YES
Vibrational data (^{15}N)	# mode symmetry wave number IR intensity selection rules<br># cm**(-1) km/mol IR RAMAN<br>7 e 154.46 4.98055 YES YES<br>8 e 154.46 4.98055 YES YES<br>9 a1 187.15 8.85413 YES YES<br>10 e 229.23 4.26499 YES YES<br>11 e 229.23 4.26499 YES YES<br>12 a1 409.09 19.60704 YES YES<br>13 a1 682.11 34.23770 YES YES<br>14 e 701.25 146.02183 YES YES<br>15 e 701.25 146.02183 YES YES
ROB3LYP/def2-QZVP (calculation in C_s using Molpro)	
Cartesian coordinates	5<br>RKS-SCF000/DEF2-QZVP ENERGY=-1617.96164679<br>N 0.0038072789 -1.5899360016 0.0000000000<br>Fe 0.0008517711 -0.1103621721 0.0000000000<br>F 1.6106905567 0.5217013431 0.0000000000<br>F -0.8080562228 0.5168697998 -1.3940348323<br>F -0.8080562228 0.5168697998 1.3940348323
Vibrational data (^{14}N)	1 A 2 A 3 A 4 A 5 A<br>Wavenumbers [cm-1] 174.99 175.46 246.80 326.86 327.00<br>Intensities [km/mol] 3.79 3.73 7.88 1.40 1.37<br>Intensities [relative] 3.03 2.98 6.30 1.12 1.09<br><br>6 A 7 A 8 A 9 A<br>Wavenumbers [cm-1] 679.42 715.22 715.76 1191.56<br>Intensities [km/mol] 51.67 124.61 124.98 51.54

	Intensities [relative]			41.34	99.70	100.00	41.24	
BP86/def2-QZVP								
Cartesian coordinates	5							
	Energy = -1618.471358935							
	Fe	-0.0000000	0.0000000	-0.0870506				
	N	0.0000000	0.0000000	-1.5977653				
	F	0.8079015	-1.3993265	0.5597309				
	F	0.8079015	1.3993265	0.5597309				
	F	-1.6158030	0.0000000	0.5597309				
Vibrational data (^{14}N)	#	mode	symmetry	wave number	IR intensity	selection rules		
	#			cm**(-1)	km/mol	IR	RAMAN	
		7	e	167.39	2.87179	YES	YES	
		8	e	167.39	2.87179	YES	YES	
		9	a1	239.69	5.49977	YES	YES	
		10	e	309.72	1.28113	YES	YES	
		11	e	309.72	1.28113	YES	YES	
		12	a1	650.45	39.90483	YES	YES	
		13	e	691.28	100.10245	YES	YES	
		14	e	691.28	100.10245	YES	YES	
		15	a1	1096.45	33.93383	YES	YES	
Vibrational data (^{15}N)	#	mode	symmetry	wave number	IR intensity	selection rules		
	#			cm**(-1)	km/mol	IR	RAMAN	
		7	e	167.13	2.80366	YES	YES	
		8	e	167.13	2.80366	YES	YES	
		9	a1	239.10	5.46806	YES	YES	
		10	e	304.02	1.39198	YES	YES	
		11	e	304.02	1.39198	YES	YES	
		12	a1	649.45	39.11997	YES	YES	
		13	e	691.27	100.10346	YES	YES	
		14	e	691.27	100.10346	YES	YES	
		15	a1	1067.95	34.07242	YES	YES	

M06-L/def2-QZVP	
Cartesian coordinates	5<br>Energy = -1618.108613779<br>N -0.0000000 0.0000000 -1.6162454<br>Fe 0.0000000 0.0000000 -0.0709558<br>F -0.8100550 1.4030564 0.5625015<br>F -0.8100550 -1.4030564 0.5625015<br>F 1.6201100 0.0000000 0.5625015
Vibrational data (^{14}N)	# mode symmetry wave number IR intensity selection rules<br># cm**(-1) km/mol IR RAMAN<br>7 e 167.17 3.24683 YES YES<br>8 e 167.17 3.24683 YES YES<br>9 a1 226.92 5.48559 YES YES<br>10 e 281.36 3.86380 YES YES<br>11 e 281.36 3.86380 YES YES<br>12 a1 617.34 42.84699 YES YES<br>13 e 703.25 126.64097 YES YES<br>14 e 703.25 126.64097 YES YES<br>15 a1 784.68 11.78418 YES YES
Vibrational data (^{15}N)	# mode symmetry wave number IR intensity selection rules<br># cm**(-1) km/mol IR RAMAN<br>7 e 166.73 3.09705 YES YES<br>8 e 166.73 3.09705 YES YES<br>9 a1 226.58 5.49940 YES YES<br>10 e 276.50 4.10189 YES YES<br>11 e 276.50 4.10189 YES YES<br>12 a1 608.24 39.09826 YES YES<br>13 e 703.25 126.64091 YES YES<br>14 e 703.25 126.64091 YES YES<br>15 a1 773.68 15.13882 YES YES
CCSD(T)/aug-cc-pVTZ (calculation in C_s)	

Cartesian coordinates	5<br>UCCSD(T)/AUG-CC-PVTZ ENERGY=-1616.58884135<br>N -0.0000000000 -1.6558196996 0.0000000000<br>Fe -0.0000000000 -0.0441039939 0.0000000000<br>F 1.6861214269 0.4501368796 0.0000000000<br>F -0.8430607134 0.4501368796 -1.4602239896<br>F -0.8430607134 0.4501368796 1.4602239896
Vibrational data (^{14}N)	1 A` 2 A`` 3 A` 4 A`` 5 A`<br>Wavenumbers [cm-1] 87.62 132.56 184.09 225.79 231.05<br>Intensities [km/mol] 0.00 0.00 0.00 0.00 0.00<br>Intensities [relative] 0.00 0.00 0.00 0.00 0.00<br><br>6 A` 7 A`` 8 A` 9 A`<br>Wavenumbers [cm-1] 537.56 718.94 720.08 757.29<br>Intensities [km/mol] 0.00 0.00 0.00 0.00<br>Intensities [relative] 0.00 0.00 0.00 0.00
Vibrational data (^{15}N)	1 A` 2 A`` 3 A` 4 A`` 5 A`<br>Wavenumbers [cm-1] 87.66 132.40 183.78 221.65 226.62<br>Intensities [km/mol] 0.00 0.00 0.00 0.00 0.00<br>Intensities [relative] 0.00 0.00 0.00 0.00 0.00<br><br>6 A` 7 A`` 8 A` 9 A`<br>Wavenumbers [cm-1] 528.75 718.93 720.03 747.74<br>Intensities [km/mol] 0.00 0.00 0.00 0.00<br>Intensities [relative] 0.00 0.00 0.00 0.00
CCSD(T)/aug-cc-pVTZ(Fe: aug-cc-pVTZ-PP) (calculation in C_s)	
Cartesian coordinates	5<br>UCCSD(T)/USERDEF ENERGY=-1625.76907210<br>Fe 0.0000327714 0.0000000000 0.0052274647<br>N 0.0000103441 0.0000000000 -1.5776279214<br>F 0.8343780343 -1.4450891256 0.5222495883

	F 0.8343780343 1.4450891256 0.5222495883<br>F -1.6687991842 0.0000000000 0.5222780802
Vibrational data (^{14}N)	1 A 2 A 3 A 4 A 5 A<br>Wavenumbers [cm-1] 60.63 67.35 185.20 242.61 253.84<br>Intensities [km/mol] 0.00 0.00 0.00 0.00 0.00<br>Intensities [relative] 0.00 0.00 0.00 0.00 0.00<br><br>6 A 7 A 8 A 9 A<br>Wavenumbers [cm-1] 563.67 751.03 752.58 791.12<br>Intensities [km/mol] 0.00 0.00 0.00 0.00<br>Intensities [relative] 0.00 0.00 0.00 0.00
Vibrational data (^{15}N)	1 A 2 A 3 A 4 A 5 A<br>Wavenumbers [cm-1] 61.02 67.00 185.01 238.71 249.60<br>Intensities [km/mol] 0.00 0.00 0.00 0.00 0.00<br>Intensities [relative] 0.00 0.00 0.00 0.00 0.00<br><br>6 A 7 A 8 A 9 A<br>Wavenumbers [cm-1] 555.79 751.01 752.58 779.51<br>Intensities [km/mol] 0.00 0.00 0.00 0.00<br>Intensities [relative] 0.00 0.00 0.00 0.00
NEVPT2/aug-cc-pVTZ-DK(Fe: aug-cc-pwCVTZ-DK)	
Cartesian coordinates	5<br>NEVPT2/AUG-CC-PVTZ-DK,FE=AUG-CC-PWCVTZ-DK ENERGY=-1626.14489052<br>N -0.0000000000 -1.5990266515 0.0000000000<br>Fe -0.0000000000 -0.0653517671 0.0000000000<br>F 1.6341336229 0.4774937965 -0.0000000000<br>F -0.8170668115 0.4774937965 -1.4152012306<br>F -0.8170668115 0.4774937965 1.4152012306
Vibrational data (^{14}N)	1 A`` 2 A` 3 A` 4 A` 5 A``<br>Wavenumbers [cm-1] 185.48 198.28 235.04 287.16 288.62<br>Intensities [km/mol] 0.00 0.00 0.00 0.00 0.00

	Intensities [relative] 0.00 0.00 0.00 0.00 0.00<br><br>6 A` 7 A` 8 A`` 9 A`<br>Wavenumbers [cm-1] 689.00 735.27 737.02 1028.10<br>Intensities [km/mol] 0.00 0.00 0.00 0.00<br>Intensities [relative] 0.00 0.00 0.00 0.00
Vibrational data (^{15}N)	1 A`` 2 A` 3 A` 4 A` 5 A``<br>Wavenumbers [cm-1] 184.89 197.82 234.44 282.06 283.63<br>Intensities [km/mol] 0.00 0.00 0.00 0.00 0.00<br>Intensities [relative] 0.00 0.00 0.00 0.00 0.00<br><br>6 A` 7 A` 8 A`` 9 A`<br>Wavenumbers [cm-1] 686.96 735.27 737.02 1002.48<br>Intensities [km/mol] 0.00 0.00 0.00 0.00<br>Intensities [relative] 0.00 0.00 0.00 0.00
**$NFeF_3$ (^{1}A') (Minimum)**	
BP86/def2-QZVP	
Cartesian coordinates	Energy = -1618.444955695<br>Fe -0.1689715 -0.1012646 0.0000000<br>N 0.0640009 -1.5823645 0.0000000<br>F -0.6719125 0.5632119 -1.5170175<br>F -0.6719125 0.5632119 1.5170175<br>F 1.4487958 0.5572052 0.0000000
Vibrational data (^{14}N)	# mode symmetry wave number IR intensity selection rules<br># cm**(-1) km/mol IR RAMAN<br>7 a" 139.05 10.76696 YES YES<br>8 a' 176.29 2.38009 YES YES<br>9 a" 243.03 0.92703 YES YES<br>10 a' 252.96 5.65554 YES YES<br>11 a' 341.25 3.41039 YES YES<br>12 a' 658.86 36.50823 YES YES

	13 a' 681.41 49.59544 YES YES<br>14 a" 716.89 138.83798 YES YES<br>15 a' 1140.00 36.98015 YES YES
NEVPT2/cc-pVTZ-DK(Fe: cc-pwCVTZ-DK)	
Cartesian coordinates	5<br>NEVPT2/CC-PVTZ-DK,FE=CC-PWCVTZ-DK ENERGY=-1625.64312761<br>N 0.0000000000 1.5197704505 -0.4497517400<br>Fe 0.0000000000 0.0305775786 -0.1575755234<br>F 0.0000000000 0.0409493994 1.5408424945<br>F -1.5405046256 -0.6556641550 -0.3679087035<br>F 1.5405046256 -0.6556641550 -0.3679087035
**$NFeF_3$ ($^1A'$) (Saddle Point)**	
BP86/def2-QZVP	
Cartesian coordinates	5<br>Energy = -1618.385851291<br>Fe 0.1370322 -0.0946389 0.0000000<br>N -0.0030579 -1.5891218 0.0000000<br>F 1.6890360 0.6580319 0.0000000<br>F -0.9115051 0.5128644 -1.2485916<br>F -0.9115051 0.5128644 1.2485916
Vibrational data (^{14}N)	# mode symmetry wave number IR intensity selection rules<br># cm**(-1) km/mol IR RAMAN<br>1 a" -137.76 0.00000 YES YES<br>8 a' 186.96 2.88662 YES YES<br>9 a" 231.33 2.45155 YES YES<br>10 a' 264.67 3.86482 YES YES<br>11 a' 345.09 0.44466 YES YES<br>12 a' 654.67 37.76467 YES YES<br>13 a" 659.70 70.04001 YES YES<br>14 a' 723.86 96.82317 YES YES<br>15 a' 1137.00 38.14481 YES YES

NEVPT2/aug-cc-pVTZ-DK(Fe: aug-cc-pwCVTZ-DK)	
Cartesian coordinates	5<br>NEVPT2/AUG-CC-PVTZ-DK,FE=AUG-CC-PWCVTZ-DK ENERGY=-1625.67617024<br>N 0.0000000000 1.5680654701 -0.2682192113<br>Fe 0.0000000000 0.0965028169 0.0915728716<br>F 0.0000000000 -0.3948210436 1.7247955065<br>F -1.2226253765 -0.5517856743 -0.8903622078<br>F 1.2226253765 -0.5517856743 -0.8903622078
Vibrational data (^{14}N)	1 A 2 A 3 A 4 A 5 A<br>Wavenumbers [cm-1] 203.81 257.26 283.08 357.88 709.22<br>Intensities [km/mol] 0.00 0.00 0.00 0.00 0.00<br>Intensities [relative] 0.00 0.00 0.00 0.00 0.00<br><br>6 A 7 A 8 A<br>Wavenumbers [cm-1] 750.53 802.85 1075.06<br>Intensities [km/mol] 0.00 0.00 0.00<br>Intensities [relative] 0.00 0.00 0.00<br><br>Normal Modes of imaginary frequencies<br><br>1<br>Wavenumbers [cm-1] 184.60<br>Intensities [km/mol] 0.00<br>Intensities [relative] 0.00
Vibrational data (^{15}N)	1 A 2 A 3 A 4 A 5 A<br>Wavenumbers [cm-1] 202.78 253.36 282.35 352.12 707.97<br>Intensities [km/mol] 0.00 0.00 0.00 0.00 0.00<br>Intensities [relative] 0.00 0.00 0.00 0.00 0.00<br><br>6 A 7 A 8 A<br>Wavenumbers [cm-1] 750.48 802.84 1047.17

	```<br>Intensities [km/mol] 0.00 0.00 0.00<br>Intensities [relative] 0.00 0.00 0.00<br><br>1<br>Wavenumbers [cm-1] 183.99<br>Intensities [km/mol] 0.00<br>Intensities [relative] 0.00<br>```
**$NFeF_2$ ($^2A_2$ – C_{2v})**	
BP86/def2-QZVP	
Cartesian coordinates	```<br>4<br>Energy = -1518.570495693<br>Fe 0.0000000 0.0000000 -0.0226156<br>N 0.0000000 0.0000000 -1.5257077<br>F -1.5299908 0.0000000 0.7741617<br>F 1.5299908 0.0000000 0.7741617<br>```
Vibrational data (^{14}N)	```<br># mode symmetry wave number IR intensity selection rules<br># cm**(-1) km/mol IR RAMAN<br>7 b2 182.27 15.07020 YES YES<br>8 a1 184.10 6.20329 YES YES<br>9 b1 288.27 0.77438 YES YES<br>10 a1 654.78 46.58282 YES YES<br>11 b1 751.39 126.02097 YES YES<br>12 a1 1104.72 48.10863 YES YES<br>```
**$NFeF_2$ ($^4A''$)**	
B3LYP/def2-QZVP	
Cartesian coordinates	```<br>4<br>Energy = -1518.119228568<br>Fe 0.0278021 0.0773987 0.0000000<br>N -0.5077336 -1.4284634 0.0000000<br>F 0.2399658 0.6755324 1.6257462<br>```

	F 0.2399658 0.6755324 -1.6257462
Vibrational data (^{14}N)	# mode symmetry wave number IR intensity selection rules<br># cm**(-1) km/mol IR RAMAN<br>7 a' 145.26 46.55205 YES YES<br>8 a' 176.76 14.75617 YES YES<br>9 a" 268.15 0.39399 YES YES<br>10 a' 628.29 36.09440 YES YES<br>11 a' 756.21 21.54315 YES YES<br>12 a" 763.68 179.24321 YES YES
BP86/def2-QZVP	
Cartesian coordinates	4<br>Energy = -1518.550298159<br>Fe 0.0166550 0.0535941 0.0000000<br>N -0.5042037 -1.4207936 0.0000000<br>F 0.2437743 0.6835998 1.6045511<br>F 0.2437743 0.6835998 -1.6045511
Vibrational data (^{14}N)	# mode symmetry wave number IR intensity selection rules<br># cm**(-1) km/mol IR RAMAN<br>7 a' 124.87 33.26064 YES YES<br>8 a' 193.76 6.56351 YES YES<br>9 a" 295.52 0.14397 YES YES<br>10 a' 637.14 36.05705 YES YES<br>11 a" 757.36 139.60363 YES YES<br>12 a' 976.46 24.15930 YES YES
M06-L/def2-QZVP	
Cartesian coordinates	4<br>Energy = -1518.218305422<br>Fe 0.0210806 0.0597000 0.0000000<br>N -0.5082336 -1.4302536 0.0000000<br>F 0.2435765 0.6852768 1.6070787<br>F 0.2435765 0.6852768 -1.6070787

Vibrational data (^{14}N)	`#  mode     symmetry     wave number   IR intensity    selection rules`<br>`#                          cm**(-1)        km/mol          IR     RAMAN`<br>`      7        a'              154.58        39.31281         YES      YES`<br>`      8        a'              182.44        10.64684         YES      YES`<br>`      9        a"              283.39         0.59237         YES      YES`<br>`     10        a'              644.96        38.30042         YES      YES`<br>`     11        a"              773.87       161.72457         YES      YES`<br>`     12        a'              859.04        23.34863         YES      YES`
**NFeF ($^1A'$)**	
B3LYP/def2-QZVP	
Cartesian coordinates	`3`<br>`Energy = -1418.196942249`<br>`N    -0.8352737    1.2834060    0.0000000`<br>`Fe   -0.2283230   -0.0402701    0.0000000`<br>`F     1.0635968   -1.2431359    0.0000000`
Vibrational data (^{14}N)	`#  mode     symmetry     wave number   IR intensity    selection rules`<br>`#                          cm**(-1)        km/mol          IR     RAMAN`<br>`      7        a'              110.26        36.90356         YES      YES`<br>`      8        a'              659.63       125.43469         YES      YES`<br>`      9        a'             1255.90       147.67327         YES      YES`
BP86/def2-QZVP	
Cartesian coordinates	`3`<br>`Energy = -1418.614447922`<br>`N    -0.7194815    1.3410679    0.0000000`<br>`Fe   -0.3383297   -0.1457525    0.0000000`<br>`F     1.0578112   -1.1953155    0.0000000`
Vibrational data (^{14}N)	`#  mode     symmetry     wave number   IR intensity    selection rules`<br>`#                          cm**(-1)        km/mol          IR     RAMAN`<br>`      7        a'              166.18        10.02727         YES      YES`<br>`      8        a'              672.08        81.59539         YES      YES`

	9 a' 1023.03 91.37623 YES YES
M06-L/def2-QZVP	
Cartesian coordinates	3<br>Energy = -1418.264492520<br>N -0.6880505 1.2722872 0.0000000<br>Fe -0.3869861 -0.1672201 0.0000000<br>F 1.0750366 -1.1050671 0.0000000
Vibrational data (^{14}N)	# mode symmetry wave number IR intensity selection rules<br># cm**(-1) km/mol IR RAMAN<br>7 a' 155.21 14.64147 YES YES<br>8 a' 679.16 108.90657 YES YES<br>9 a' 1200.36 92.81648 YES YES
**$NRuF_3$ (^{1}A')**	
B3LYP/def2-QZVP	
Cartesian coordinates	5<br>Energy = -449.2125490913<br>Ru -0.2295315 -0.1671942 0.0000000<br>N 0.2078405 -1.6762571 0.0000000<br>F 1.4156439 0.7112763 0.0000000<br>F -0.6969764 0.5660875 -1.6557313<br>F -0.6969764 0.5660875 1.6557313
Vibrational data (^{14}N)	# mode symmetry wave number IR intensity selection rules<br># cm**(-1) km/mol IR RAMAN<br>7 a" 140.48 11.58363 YES YES<br>8 a' 145.40 4.18558 YES YES<br>9 a" 221.42 1.28794 YES YES<br>10 a' 224.40 7.85554 YES YES<br>11 a' 326.12 6.61481 YES YES<br>12 a' 633.58 57.94256 YES YES<br>13 a" 660.55 170.88815 YES YES

	14 a' 669.35 53.63524 YES YES<br>15 a' 1201.63 42.63381 YES YES
Vibrational data (^{15}N)	# mode symmetry wave number IR intensity selection rules<br># cm**(-1) km/mol IR RAMAN<br>7 a" 140.06 11.68921 YES YES<br>8 a' 145.22 4.09724 YES YES<br>9 a" 217.00 1.12343 YES YES<br>10 a' 224.23 7.81067 YES YES<br>11 a' 319.83 6.73314 YES YES<br>12 a' 633.48 57.78115 YES YES<br>13 a" 660.55 170.86323 YES YES<br>14 a' 669.27 53.47856 YES YES<br>15 a' 1166.20 41.31606 YES YES
BP86/def2-QZVP	
Cartesian coordinates	5<br>Energy = -449.5409092607<br>Ru -0.2254464 -0.1681867 0.0000000<br>N 0.2076329 -1.6993337 0.0000000<br>F 1.4202437 0.7209890 0.0000000<br>F -0.7012151 0.5732657 -1.6574811<br>F -0.7012151 0.5732657 1.6574811
Vibrational data (^{14}N)	# mode symmetry wave number IR intensity selection rules<br># cm**(-1) km/mol IR RAMAN<br>7 a" 115.45 12.37316 YES YES<br>8 a' 140.86 2.87881 YES YES<br>9 a" 210.51 1.49515 YES YES<br>10 a' 218.62 6.01410 YES YES<br>11 a' 315.87 5.48997 YES YES<br>12 a' 617.82 48.43595 YES YES<br>13 a" 640.58 149.89386 YES YES<br>14 a' 651.14 45.50603 YES YES

	15 a' 1150.27 40.07358 YES YES
Vibrational data (^{15}N)	# mode symmetry wave number IR intensity selection rules<br># cm**(-1) km/mol IR RAMAN<br>7 a" 115.12 12.43546 YES YES<br>8 a' 140.69 2.81783 YES YES<br>9 a" 206.32 1.32536 YES YES<br>10 a' 218.45 5.98200 YES YES<br>11 a' 309.78 5.54414 YES YES<br>12 a' 617.70 48.26582 YES YES<br>13 a" 640.58 149.87678 YES YES<br>14 a' 651.07 45.37272 YES YES<br>15 a' 1116.38 38.76958 YES YES
CCSD(T)/aug-cc-pVTZ(-PP)	
Cartesian coordinates	5<br>UCCSD(T)/USERDEF ENERGY=-448.02376908<br>Ru -0.2482196139 -0.1415726171 0.0000000000<br>N 0.2058519177 -1.6683109472 0.0000000000<br>F 1.4036142992 0.7101998411 0.0000000000<br>F -0.6806233015 0.5498418616 -1.6786455388<br>F -0.6806233015 0.5498418616 1.6786455388
Vibrational data (^{14}N)	1 A 2 A 3 A 4 A 5 A<br>Wavenumbers [cm-1] 153.51 158.33 225.86 226.88 321.89<br>Intensities [km/mol] 0.00 0.00 0.00 0.00 0.00<br>Intensities [relative] 0.00 0.00 0.00 0.00 0.00<br><br>6 A 7 A 8 A 9 A<br>Wavenumbers [cm-1] 648.82 677.96 681.64 1084.72<br>Intensities [km/mol] 0.00 0.00 0.00 0.00<br>Intensities [relative] 0.00 0.00 0.00 0.00
Vibrational data (^{15}N)	1 A 2 A 3 A 4 A 5 A

	Wavenumbers [cm-1] 153.34 157.57 222.51 225.67 315.86<br>Intensities [km/mol] 0.00 0.00 0.00 0.00 0.00<br>Intensities [relative] 0.00 0.00 0.00 0.00 0.00<br><br>6 A 7 A 8 A 9 A<br>Wavenumbers [cm-1] 648.71 677.96 681.56 1052.62<br>Intensities [km/mol] 0.00 0.00 0.00 0.00<br>Intensities [relative] 0.00 0.00 0.00 0.00
**$NRuF_3$ ($^1A'$) (Saddle point)**	
B3LYP/def2-QZVP	
Cartesian coordinates	5<br>Energy = -449.2092804041<br>Ru 0.1926203 -0.1308375 0.0000000<br>N 0.0261138 -1.6957358 0.0000000<br>F 1.8046906 0.8160869 0.0000000<br>F -1.0117123 0.5052432 -1.2757559<br>F -1.0117123 0.5052432 1.2757559
Vibrational data (^{14}N)	# mode symmetry wave number IR intensity selection rules<br># cm**(-1) km/mol IR RAMAN<br>1 a" -136.27 0.00000 YES YES<br>8 a' 158.95 6.85409 YES YES<br>9 a" 193.35 1.11396 YES YES<br>10 a' 243.31 3.40732 YES YES<br>11 a' 334.22 1.79481 YES YES<br>12 a' 638.29 97.80838 YES YES<br>13 a" 640.84 77.73013 YES YES<br>14 a' 683.23 91.14722 YES YES<br>15 a' 1193.73 47.60703 YES YES
BP86/def2-QZVP	
Cartesian coordinates	5

	Energy = -449.5385900192<br>Ru 0.1889815 -0.1344266 0.0000000<br>N -0.0290101 -1.7134782 0.0000000<br>F 1.8479076 0.7343642 0.0000000<br>F -1.0039395 0.5567703 -1.2679319<br>F -1.0039395 0.5567703 1.2679319
Vibrational data (^{14}N)	# mode symmetry wave number IR intensity selection rules<br># cm**(-1) km/mol IR RAMAN<br>1 a" -113.31 0.00000 YES YES<br>8 a' 151.50 5.18453 YES YES<br>9 a" 186.13 1.62067 YES YES<br>10 a' 240.19 2.30781 YES YES<br>11 a' 324.04 1.57113 YES YES<br>12 a' 622.14 74.33507 YES YES<br>13 a" 623.64 66.46453 YES YES<br>14 a' 665.90 82.85495 YES YES<br>15 a' 1141.27 42.70175 YES YES
CCSD(T)/aug-cc-pVTZ(-PP)	
Cartesian coordinates	5<br>UCCSD(T)/AUG-CC-PVTZ,RU=AUG-CC-PVTZ-PP ENERGY=-448.01978832<br>Ru 0.1989302016 -0.1071433142 0.0000000000<br>N 0.0200330139 -1.6935334021 0.0000000000<br>F 1.8246345469 0.8108776158 0.0000000000<br>F -1.0217988312 0.4948995503 -1.2677897058<br>F -1.0217988312 0.4948995503 1.2677897058
Vibrational data (^{14}N)	1 A 2 A 3 A 4 A 5 A<br>Wavenumbers [cm-1] 157.02 194.48 245.22 324.61 648.05<br>Intensities [km/mol] 0.00 0.00 0.00 0.00 0.00<br>Intensities [relative] 0.00 0.00 0.00 0.00 0.00<br><br>6 A 7 A 8 A

| | ```
 Wavenumbers [cm-1]        657.45      695.12     1070.31
 Intensities [km/mol]        0.00        0.00        0.00
 Intensities [relative]      0.00        0.00        0.00

                                1
 Wavenumbers [cm-1]        137.00
 Intensities [km/mol]        0.00
 Intensities [relative]      0.00
``` |
| Vibrational data ($^{15}N$) | ```
                             1 A         2 A         3 A         4 A         5 A
 Wavenumbers [cm-1]        156.65      191.66      244.55      318.53      647.86
 Intensities [km/mol]        0.00        0.00        0.00        0.00        0.00
 Intensities [relative]      0.00        0.00        0.00        0.00        0.00

                             6 A         7 A         8 A
 Wavenumbers [cm-1]        657.43      695.09     1038.47
 Intensities [km/mol]        0.00        0.00        0.00
 Intensities [relative]      0.00        0.00        0.00

                                1
 Wavenumbers [cm-1]        136.16
 Intensities [km/mol]        0.00
 Intensities [relative]      0.00
``` |
| **$NRuF_3$ ($^3A_2$ – C_{3v})** | |
| B3LYP/def2-QZVP | |
| Cartesian coordinates | ```
5
Energy = -449.2162041256
Ru    0.0000000    0.0000000   -0.1583439
N    -0.0000000    0.0000000   -1.7354498
F    -1.7027133    0.0000000    0.6320504
F     0.8513567   -1.4745930    0.6320504
F     0.8513567    1.4745930    0.6320504
``` |
```

Vibrational data (^{14}N)	# mode symmetry wave number IR intensity selection rules<br># cm**(-1) km/mol IR RAMAN<br>7 e 115.55 3.84713 YES YES<br>8 e 115.55 3.84713 YES YES<br>9 a1 195.70 8.77212 YES YES<br>10 e 282.64 3.45947 YES YES<br>11 e 282.64 3.45947 YES YES<br>12 e 627.89 113.45195 YES YES<br>13 e 627.89 113.45195 YES YES<br>14 a1 641.71 72.39758 YES YES<br>15 a1 1186.13 57.91788 YES YES
Vibrational data (^{15}N)	# mode symmetry wave number IR intensity selection rules<br># cm**(-1) km/mol IR RAMAN<br>7 e 115.45 3.78791 YES YES<br>8 e 115.45 3.78791 YES YES<br>9 a1 195.49 8.74429 YES YES<br>10 e 276.80 3.57756 YES YES<br>11 e 276.80 3.57756 YES YES<br>12 e 627.88 113.42613 YES YES<br>13 e 627.88 113.42613 YES YES<br>14 a1 641.54 71.98906 YES YES<br>15 a1 1151.02 56.04822 YES YES
BP86/def2-QZVP	
Cartesian coordinates	5<br>Energy = -449.5464924977<br>Ru 0.0000000 0.0000000 -0.1597160<br>N 0.0000000 0.0000000 -1.7574356<br>F -1.7057368 0.0000000 0.6398363<br>F 0.8528684 -1.4772114 0.6398363<br>F 0.8528684 1.4772114 0.6398363
Vibrational data (^{14}N)	# mode symmetry wave number IR intensity selection rules

	# cm**(-1) km/mol IR RAMAN<br>7 e 102.92 3.14908 YES YES<br>8 e 102.92 3.14908 YES YES<br>9 a1 190.87 6.58499 YES YES<br>10 e 274.09 2.42345 YES YES<br>11 e 274.09 2.42345 YES YES<br>12 e 612.41 92.78843 YES YES<br>13 e 612.41 92.78843 YES YES<br>14 a1 623.13 57.24195 YES YES<br>15 a1 1131.95 51.81268 YES YES
Vibrational data (^{15}N)	# mode symmetry wave number IR intensity selection rules<br># cm**(-1) km/mol IR RAMAN<br>7 e 102.84 3.10901 YES YES<br>8 e 102.84 3.10901 YES YES<br>9 a1 190.66 6.56295 YES YES<br>10 e 268.43 2.50503 YES YES<br>11 e 268.43 2.50503 YES YES<br>12 e 612.40 92.76469 YES YES<br>13 e 612.40 92.76469 YES YES<br>14 a1 622.96 56.88692 YES YES<br>15 a1 1098.43 50.06846 YES YES
CCSD(T)/aug-cc-pVTZ(-PP) (calculation in C_s)	
Cartesian coordinates	5<br>UCCSD(T)/USERDEF ENERGY=-448.02302842<br>Ru 0.0000056843 0.0000000000 -0.1368111874<br>N 0.0000083347 0.0000000000 -1.7329376335<br>F -1.7119195925 0.0000000000 0.6240451861<br>F 0.8559527867 -1.4825466742 0.6240304674<br>F 0.8559527867 1.4825466742 0.6240304674
Vibrational data (^{14}N)	1 A 2 A 3 A 4 A 5 A<br>Wavenumbers [cm-1] 95.39 95.46 183.05 264.88 267.59

	Intensities [km/mol] 0.00 0.00 0.00 0.00 0.00<br>Intensities [relative] 0.00 0.00 0.00 0.00 0.00<br><br>6 A 7 A 8 A 9 A<br>Wavenumbers [cm-1] 638.89 639.04 650.91 1121.62<br>Intensities [km/mol] 0.00 0.00 0.00 0.00<br>Intensities [relative] 0.00 0.00 0.00 0.00
Vibrational data (^{15}N)	1 A 2 A 3 A 4 A 5 A<br>Wavenumbers [cm-1] 95.26 95.36 182.88 259.60 262.24<br>Intensities [km/mol] 0.00 0.00 0.00 0.00 0.00<br>Intensities [relative] 0.00 0.00 0.00 0.00 0.00<br><br>6 A 7 A 8 A 9 A<br>Wavenumbers [cm-1] 638.89 639.03 650.65 1088.43<br>Intensities [km/mol] 0.00 0.00 0.00 0.00<br>Intensities [relative] 0.00 0.00 0.00 0.00
**$FNRuF_2$ ($^3A''$)**	
B3LYP/def2-QZVP	
Cartesian coordinates	5<br>Energy = -449.1573313462<br>Ru -0.4310211 0.4631061 0.0000000<br>N 0.0458104 -1.1766224 0.0000000<br>F 1.4275960 -1.4284082 0.0000000<br>F -0.5211927 1.0709622 -1.7581990<br>F -0.5211927 1.0709622 1.7581990
Vibrational data (^{14}N)	# mode symmetry wave number IR intensity selection rules<br># cm**(-1) km/mol IR RAMAN<br>7 a" 115.51 0.96486 YES YES<br>8 a' 132.78 15.96700 YES YES<br>9 a' 149.10 7.02535 YES YES<br>10 a' 250.27 0.22400 YES YES

	11 a" 336.67 0.04722 YES YES<br>12 a' 614.84 70.22821 YES YES<br>13 a" 674.82 197.22465 YES YES<br>14 a' 722.98 197.44424 YES YES<br>15 a' 857.38 107.19311 YES YES
Vibrational data (^{15}N)	# mode symmetry wave number IR intensity selection rules<br># cm**(-1) km/mol IR RAMAN<br>7 a" 115.50 0.96403 YES YES<br>8 a' 132.72 15.93472 YES YES<br>9 a' 148.93 7.05612 YES YES<br>10 a' 247.82 0.22187 YES YES<br>11 a" 327.78 0.03526 YES YES<br>12 a' 614.58 70.42724 YES YES<br>13 a" 674.77 197.23201 YES YES<br>14 a' 711.24 184.78323 YES YES<br>15 a' 833.26 107.86564 YES YES
BP86/def2-QZVP	
Cartesian coordinates	5<br>Energy = -449.4844274026<br>Ru -0.4264655 0.4724047 0.0000000<br>N 0.0267719 -1.1687116 0.0000000<br>F 1.4542388 -1.4447396 0.0000000<br>F -0.5272726 1.0705233 -1.7612453<br>F -0.5272726 1.0705233 1.7612453
Vibrational data (^{14}N)	# mode symmetry wave number IR intensity selection rules<br># cm**(-1) km/mol IR RAMAN<br>7 a" 115.38 0.79904 YES YES<br>8 a' 129.02 11.53677 YES YES<br>9 a' 150.83 4.98091 YES YES<br>10 a' 228.62 0.53805 YES YES<br>11 a" 332.38 0.17070 YES YES

	12 a' 607.72 150.98231 YES YES<br>13 a' 620.36 189.35179 YES YES<br>14 a" 669.89 165.83574 YES YES<br>15 a' 833.66 18.85258 YES YES
**F_2NRuF ($^3A''$)**	
B3LYP/def2-QZVP	
Cartesian coordinates	5<br>Energy = -449.0673348158<br>N 0.6062182 0.4784034 0.0000000<br>F 1.4938959 0.3814864 1.0732974<br>F 1.4938959 0.3814864 -1.0732974<br>Ru -1.1285816 0.0390172 0.0000000<br>F -2.4654284 -1.2803935 0.0000000
Vibrational data (^{14}N)	# mode symmetry wave number IR intensity selection rules<br># cm**(-1) km/mol IR RAMAN<br>7 a' 71.79 9.50790 YES YES<br>8 a" 130.98 3.59864 YES YES<br>9 a" 273.92 2.10363 YES YES<br>10 a' 352.34 10.41625 YES YES<br>11 a' 468.62 1.17255 YES YES<br>12 a' 590.89 6.35660 YES YES<br>13 a' 627.83 165.07653 YES YES<br>14 a" 720.57 170.25053 YES YES<br>15 a' 937.89 348.98867 YES YES
BP86/def2-QZVP	
Cartesian coordinates	5<br>Energy = -449.3864616213<br>N 0.5871947 0.4382405 0.0000000<br>F 1.5243100 0.4232248 1.0886925<br>F 1.5243100 0.4232248 -1.0886925

	Ru -1.1010054 -0.0354701 0.0000000<br>F -2.5348091 -1.2492200 0.0000000
Vibrational data (^{14}N)	# mode symmetry wave number IR intensity selection rules<br># cm**(-1) km/mol IR RAMAN<br>7 a' 64.81 7.04519 YES YES<br>8 a" 120.26 2.53828 YES YES<br>9 a" 287.67 0.47676 YES YES<br>10 a' 362.44 10.22412 YES YES<br>11 a' 462.12 0.30631 YES YES<br>12 a" 471.55 198.75532 YES YES<br>13 a' 560.04 22.35746 YES YES<br>14 a' 626.23 187.74773 YES YES<br>15 a' 888.59 369.83161 YES YES
**$NRuF_2$ ($^2A_1$ – C_{2v})**	
B3LYP/def2-QZVP	
Cartesian coordinates	4<br>Energy = -349.3592709467<br>Ru 0.0000000 0.0000000 -0.1130509<br>F -1.5983451 0.0000000 0.8968725<br>F 1.5983451 0.0000000 0.8968725<br>N 0.0000000 0.0000000 -1.6806942
Vibrational data (^{14}N)	# mode symmetry wave number IR intensity selection rules<br># cm**(-1) km/mol IR RAMAN<br>7 a1 150.84 8.35183 YES YES<br>8 b2 217.85 21.92131 YES YES<br>9 b1 226.33 6.66585 YES YES<br>10 a1 620.25 80.24791 YES YES<br>11 b1 633.64 155.17322 YES YES<br>12 a1 1199.95 70.91294 YES YES
Vibrational data (^{15}N)	# mode symmetry wave number IR intensity selection rules<br># cm**(-1) km/mol IR RAMAN

	7 a1 150.73 8.33534 YES YES<br>8 b2 215.25 22.06547 YES YES<br>9 b1 221.21 6.55719 YES YES<br>10 a1 620.09 79.84818 YES YES<br>11 b1 633.64 155.13306 YES YES<br>12 a1 1164.42 68.31747 YES YES
BP86def2-QZVP	
Cartesian coordinates	4<br>Energy = -349.6429345337<br>Ru 0.0000000 0.0000000 -0.0421047<br>F -1.6500875 0.0000000 0.8370439<br>F 1.6500875 0.0000000 0.8370439<br>N 0.0000000 0.0000000 -1.6319831
Vibrational data (^{14}N)	# mode symmetry wave number IR intensity selection rules<br># cm**(-1) km/mol IR RAMAN<br>7 b2 140.34 10.07492 YES YES<br>8 a1 140.63 6.59108 YES YES<br>9 b1 257.58 2.09602 YES YES<br>10 a1 609.43 56.61426 YES YES<br>11 b1 657.86 118.56348 YES YES<br>12 a1 1150.82 44.61683 YES YES
Vibrational data (^{15}N)	# mode symmetry wave number IR intensity selection rules<br># cm**(-1) km/mol IR RAMAN<br>7 b2 138.93 10.15924 YES YES<br>8 a1 140.52 6.57738 YES YES<br>9 b1 251.74 2.06585 YES YES<br>10 a1 609.36 56.42520 YES YES<br>11 b1 657.86 118.53145 YES YES<br>12 a1 1116.64 42.83396 YES YES
**NRuF ($^1A'$)**	

<table>
<tr><td colspan="2">B3LYP/def2-QZVP</td></tr>
<tr><td>Cartesian coordinates</td><td><pre>3
Energy = -249.4681749998
N   -0.7636745   1.4649454   0.0000000
Ru   -0.2472823   -0.0038843   0.0000000
F   1.0109568   -1.4610611   0.0000000</pre></td></tr>
<tr><td>Vibrational data (^{14}N)</td><td><pre># mode   symmetry   wave number   IR intensity   selection rules
cm**(-1)   km/mol   IR   RAMAN
     7   a'   111.36   24.92755   YES   YES
     8   a'   592.14   139.29947   YES   YES
     9   a'   1225.59   106.87720   YES   YES</pre></td></tr>
<tr><td>Vibrational data (^{15}N)</td><td><pre># mode   symmetry   wave number   IR intensity   selection rules
cm**(-1)   km/mol   IR   RAMAN
     7   a'   109.42   24.87606   YES   YES
     8   a'   591.96   138.74045   YES   YES
     9   a'   1189.16   102.52694   YES   YES</pre></td></tr>
<tr><td colspan="2">BP86/def2-QZVP</td></tr>
<tr><td>Cartesian coordinates</td><td><pre>3
Energy = -249.6962748053
N   -0.7416205   1.4724700   0.0000000
Ru   -0.2775501   -0.0310340   0.0000000
F   1.0191706   -1.4414360   0.0000000</pre></td></tr>
<tr><td>Vibrational data (^{14}N)</td><td><pre># mode   symmetry   wave number   IR intensity   selection rules
cm**(-1)   km/mol   IR   RAMAN
     7   a'   119.54   21.68419   YES   YES
     8   a'   591.60   121.27984   YES   YES
     9   a'   1180.20   93.30420   YES   YES</pre></td></tr>
<tr><td>Vibrational data (^{15}N)</td><td><pre># mode   symmetry   wave number   IR intensity   selection rules
cm**(-1)   km/mol   IR   RAMAN
     7   a'   117.46   21.59550   YES   YES</pre></td></tr>
</table>

	8 a' 591.43 120.75909 YES YES<br>9 a' 1145.15 89.53202 YES YES
**$NRuF_4$ ($^2B_2$ – C_{4v})**	
B3LYP/def2-QZVP	
Cartesian coordinates	6<br>Energy = -549.0738622897<br>N 0.0000000 0.0000000 1.6292002<br>Ru -0.0000000 0.0000000 0.0483234<br>F 1.2672066 1.2672066 -0.4193785<br>F 1.2672066 -1.2672066 -0.4193785<br>F -1.2672066 1.2672066 -0.4193785<br>F -1.2672066 -1.2672066 -0.4193785
Vibrational data (^{14}N)	# mode symmetry wave number IR intensity selection rules<br># cm**(-1) km/mol IR RAMAN<br>7 b2 122.87 0.00000 NO YES<br>8 e 253.21 7.53124 YES YES<br>9 e 253.21 7.53124 YES YES<br>10 a1 263.84 7.62219 YES YES<br>11 b1 304.78 0.00000 NO YES<br>12 e 340.05 2.61182 YES YES<br>13 e 340.05 2.61182 YES YES<br>14 b2 587.60 0.00000 NO YES<br>15 a1 669.52 26.83520 YES YES<br>16 e 694.19 196.38844 YES YES<br>17 e 694.19 196.38844 YES YES<br>18 a1 1175.13 14.54667 YES YES
Vibrational data (^{15}N)	# mode symmetry wave number IR intensity selection rules<br># cm**(-1) km/mol IR RAMAN<br>7 b2 122.87 0.00000 NO YES<br>8 e 252.22 7.19231 YES YES<br>9 e 252.22 7.19231 YES YES

	10 a1 263.40 7.58592 YES YES<br>11 b1 304.78 0.00000 NO YES<br>12 e 333.31 2.97490 YES YES<br>13 e 333.31 2.97490 YES YES<br>14 b2 587.60 0.00000 NO YES<br>15 a1 669.41 26.70163 YES YES<br>16 e 694.19 196.38607 YES YES<br>17 e 694.19 196.38607 YES YES<br>18 a1 1140.62 14.26550 YES YES
BP86/def2-QZVP	
Cartesian coordinates	6<br>Energy = -549.4590996103<br>N 0.0000000 0.0000000 1.6539447<br>Ru 0.0000000 0.0000000 0.0516735<br>F 1.2720273 1.2720273 -0.4264021<br>F 1.2720273 -1.2720273 -0.4264021<br>F -1.2720273 1.2720273 -0.4264021<br>F -1.2720273 -1.2720273 -0.4264021
Vibrational data (^{14}N)	# mode symmetry wave number IR intensity selection rules<br># cm**(-1) km/mol IR RAMAN<br>7 b2 122.62 0.00000 NO YES<br>8 e 248.84 5.37346 YES YES<br>9 e 248.84 5.37346 YES YES<br>10 a1 259.75 5.50449 YES YES<br>11 b1 296.81 0.00000 NO YES<br>12 e 332.36 2.55471 YES YES<br>13 e 332.36 2.55471 YES YES<br>14 b2 576.15 0.00000 NO YES<br>15 a1 644.78 21.65410 YES YES<br>16 e 675.41 161.85084 YES YES<br>17 e 675.41 161.85084 YES YES

	18 a1 1125.73 16.00755 YES YES
Vibrational data (^{15}N)	# mode symmetry wave number IR intensity selection rules<br># cm**(-1) km/mol IR RAMAN<br>7 b2 122.62 0.00000 NO YES<br>8 e 247.73 5.08819 YES YES<br>9 e 247.73 5.08819 YES YES<br>10 a1 259.31 5.47530 YES YES<br>11 b1 296.81 0.00000 NO YES<br>12 e 325.99 2.85933 YES YES<br>13 e 325.99 2.85933 YES YES<br>14 b2 576.15 0.00000 NO YES<br>15 a1 644.68 21.53607 YES YES<br>16 e 675.40 161.84468 YES YES<br>17 e 675.40 161.84468 YES YES<br>18 a1 1092.65 15.59090 YES YES
CCSD(T)/aug-cc-pVTZ(-PP) (Calculation in C_{2v})	
Cartesian coordinates	6<br>UCCSD(T)/AUG-CC-PVTZ,RU=AUG-CC-PVTZ-PP ENERGY=-547.76517712<br>N 0.0000000000 -0.0000000000 -1.6602280654<br>Ru 0.0000000000 -0.0000000000 -0.0536568805<br>F 1.7941816843 0.0000000000 0.3773661575<br>F 0.0000000000 1.7941816843 0.3773661575<br>F 0.0000000000 -1.7941816843 0.3773661575<br>F -1.7941816843 0.0000000000 0.3773661575
Vibrational data (^{14}N)	1 A1 2 B2 3 B1 4 A1 5 A2<br>Wavenumbers [cm-1] 112.61 248.28 248.28 254.69 296.69<br>Intensities [km/mol] 0.00 0.00 0.00 0.00 0.00<br>Intensities [relative] 0.00 0.00 0.00 0.00 0.00<br><br>6 B1 7 B2 8 A1 9 A1 10 B1<br>Wavenumbers [cm-1] 325.44 325.44 597.59 680.82 710.95

	```<br>Intensities [km/mol] 0.00 0.00 0.00 0.00 0.00<br>Intensities [relative] 0.00 0.00 0.00 0.00 0.00<br><br>11 B2 12 A1<br>Wavenumbers [cm-1] 710.95 1082.23<br>Intensities [km/mol] 0.00 0.00<br>Intensities [relative] 0.00 0.00<br>```
Vibrational data (^{15}N)	```<br>1 A1 2 B2 3 B1 4 A1 5 A2<br>Wavenumbers [cm-1] 112.61 247.21 247.21 254.28 296.69<br>Intensities [km/mol] 0.00 0.00 0.00 0.00 0.00<br>Intensities [relative] 0.00 0.00 0.00 0.00 0.00<br><br>6 B1 7 B2 8 A1 9 A1 10 B1<br>Wavenumbers [cm-1] 319.13 319.13 597.59 680.66 710.95<br>Intensities [km/mol] 0.00 0.00 0.00 0.00 0.00<br>Intensities [relative] 0.00 0.00 0.00 0.00 0.00<br><br>11 B2 12 A1<br>Wavenumbers [cm-1] 710.95 1050.30<br>Intensities [km/mol] 0.00 0.00<br>Intensities [relative] 0.00 0.00<br>```
**$NOsF_3$ ($^1A'$)**	
B3LYP/def2-QZVP	
Cartesian coordinates	5<br>Energy = -445.0147611427<br>Os 0.2118695 -0.0804130 0.0000000<br>N 0.0469604 -1.6903823 0.0000000<br>F 1.9374225 0.6457335 0.0000000<br>F -1.0981262 0.5625309 -1.2042000<br>F -1.0981262 0.5625309 1.2042000

Vibrational data (^{14}N)	# mode	symmetry	wave number cm**(-1)	IR intensity km/mol	selection rules IR	selection rules RAMAN
	7	a"	94.77	8.70859	YES	YES
	8	a'	157.51	6.34168	YES	YES
	9	a"	160.81	4.22783	YES	YES
	10	a'	288.31	0.56612	YES	YES
	11	a'	337.45	2.25736	YES	YES
	12	a"	629.21	59.32085	YES	YES
	13	a'	647.17	75.32060	YES	YES
	14	a'	671.00	93.68788	YES	YES
	15	a'	1195.66	28.31092	YES	YES

Vibrational data (^{15}N)	# mode	symmetry	wave number cm**(-1)	IR intensity km/mol	selection rules IR	selection rules RAMAN
	7	a"	94.48	8.83694	YES	YES
	8	a'	157.28	6.28614	YES	YES
	9	a"	158.01	3.95685	YES	YES
	10	a'	286.64	0.48555	YES	YES
	11	a'	331.46	2.35944	YES	YES
	12	a"	629.21	59.31508	YES	YES
	13	a'	647.12	75.41929	YES	YES
	14	a'	670.97	93.51862	YES	YES
	15	a'	1158.16	26.83453	YES	YES

BP86/def2-QZVP

Cartesian coordinates

```
5
Energy = -445.3601994103
Os    0.2150175   -0.0647964    0.0000000
N     0.0695934   -1.6932890    0.0000000
F     1.9547202    0.6231321    0.0000000
F    -1.1196656    0.5674766   -1.1923435
F    -1.1196656    0.5674766    1.1923435
```

Vibrational data (^{14}N)	# mode	symmetry	wave number	IR intensity	selection rules

	# cm**(-1) km/mol IR RAMAN<br>7 a" 112.01 2.37649 YES YES<br>8 a" 155.22 5.56160 YES YES<br>9 a' 159.91 4.14987 YES YES<br>10 a' 293.90 0.15989 YES YES<br>11 a' 333.82 1.69314 YES YES<br>12 a" 609.15 51.02886 YES YES<br>13 a' 637.49 44.75508 YES YES<br>14 a' 657.02 98.08169 YES YES<br>15 a' 1147.52 23.00545 YES YES
Vibrational data (^{15}N)	# mode symmetry wave number IR intensity selection rules<br># cm**(-1) km/mol IR RAMAN<br>7 a" 111.99 2.41526 YES YES<br>8 a" 152.14 5.40744 YES YES<br>9 a' 159.70 4.11446 YES YES<br>10 a' 291.39 0.12015 YES YES<br>11 a' 328.76 1.73281 YES YES<br>12 a" 609.15 51.01597 YES YES<br>13 a' 637.46 44.82327 YES YES<br>14 a' 656.97 97.95313 YES YES<br>15 a' 1111.53 21.78340 YES YES
M06-L/def2-QZVP	
Cartesian coordinates	5<br>Energy = -445.1765263734<br>Os 0.2145163 -0.0798466 0.0000000<br>N 0.0565914 -1.6873005 0.0000000<br>F 1.9441265 0.6368152 0.0000000<br>F -1.1076171 0.5651660 -1.1920648<br>F -1.1076171 0.5651660 1.1920648
Vibrational data (^{14}N)	# mode symmetry wave number IR intensity selection rules<br># cm**(-1) km/mol IR RAMAN

	7 a" 100.00 6.01765 YES YES<br>8 a' 155.50 5.99784 YES YES<br>9 a" 159.38 4.77145 YES YES<br>10 a' 293.35 0.39749 YES YES<br>11 a' 340.07 2.23387 YES YES<br>12 a" 619.62 59.45138 YES YES<br>13 a' 645.31 61.07871 YES YES<br>14 a' 664.75 105.02581 YES YES<br>15 a' 1181.99 31.64593 YES YES
CCSD(T)/aug-cc-pVTZ(-PP)	
Cartesian coordinates	5<br>UCCSD(T)/USERDEF ENERGY=-444.34128605<br>Os 0.2133133067 -0.0635978523 0.0000000000<br>N 0.0512604172 -1.6822834377 0.0000000000<br>F 1.9400930901 0.6320088256 0.0000000000<br>F -1.1023334070 0.5569362323 -1.1911669081<br>F -1.1023334070 0.5569362323 1.1911669081
Vibrational data (^{14}N)	1 A 2 A 3 A 4 A 5 A<br>Wavenumbers [cm-1] 112.70 166.65 168.27 301.10 344.93<br>Intensities [km/mol] 0.00 0.00 0.00 0.00 0.00<br>Intensities [relative] 0.00 0.00 0.00 0.00 0.00<br><br>6 A 7 A 8 A 9 A<br>Wavenumbers [cm-1] 651.65 664.02 688.84 1152.17<br>Intensities [km/mol] 0.00 0.00 0.00 0.00<br>Intensities [relative] 0.00 0.00 0.00 0.00
Vibrational data (^{15}N)	1 A 2 A 3 A 4 A 5 A<br>Wavenumbers [cm-1] 112.07 163.73 168.05 297.61 340.65<br>Intensities [km/mol] 0.00 0.00 0.00 0.00 0.00<br>Intensities [relative] 0.00 0.00 0.00 0.00 0.00

<table>
<tr><td></td><td>6 A 7 A 8 A 9 A
Wavenumbers [cm-1] 651.65 663.97 688.82 1115.88
Intensities [km/mol] 0.00 0.00 0.00 0.00
Intensities [relative] 0.00 0.00 0.00 0.00</td></tr>
<tr><td colspan="2">$NOsF_3$ ($^1A'$) (Saddle point)</td></tr>
<tr><td colspan="2">BP86/def2-QZVP</td></tr>
<tr><td>Cartesian coordinates</td><td>Energy = -445.3553333122
Os -0.2507953 -0.1540708 0.0000000
N 0.1022617 -1.7472001 0.0000000
F 1.4637138 0.6406218 0.0000000
F -0.6575901 0.6303245 -1.6883909
F -0.6575901 0.6303245 1.6883909</td></tr>
<tr><td>Vibrational data (^{14}N)</td><td># mode symmetry wave number IR intensity selection rules
cm**(-1) km/mol IR RAMAN
1 a" -129.53 0.00000 YES YES
8 a' 135.96 2.06117 YES YES
9 a" 201.33 3.07315 YES YES
10 a' 207.33 5.44236 YES YES
11 a' 300.99 5.27951 YES YES
12 a" 609.17 148.33817 YES YES
13 a' 621.49 42.30664 YES YES
14 a' 652.62 42.12874 YES YES
15 a' 1154.60 31.18101 YES YES</td></tr>
<tr><td colspan="2">B3LYP/def2-QZVP</td></tr>
<tr><td>Cartesian coordinates</td><td>Energy = -445.0120434426
Os -0.2421890 -0.1723479 0.0000000
N 0.2159030 -1.7216683 0.0000000
F 1.4148021 0.7269237 0.0000000
F -0.6942581 0.5835462 -1.6829258
F -0.6942581 0.5835462 1.6829258</td></tr>
</table>

Vibrational data (^{14}N)	1 a" -100.05 0.00000 YES YES<br>8 a' 139.78 3.13320 YES YES<br>9 a" 208.92 3.21573 YES YES<br>10 a' 212.48 7.24448 YES YES<br>11 a' 309.71 6.69390 YES YES<br>12 a" 626.19 163.79036 YES YES<br>13 a' 635.76 49.07285 YES YES<br>14 a' 668.62 51.00883 YES YES<br>15 a' 1201.15 34.46664 YES YES
CCSD(T)/aug-cc-pVTZ(-PP)	
Cartesian coordinates	UCCSD(T)/AUG-CC-PVTZ,OS=AUG-CC-PVTZ-PP ENERGY=-444.33811776<br>N 0.0000000000 -1.3511107882 -1.0090462066<br>Os 0.0000000000 -0.1134113509 0.0407080910<br>F 0.0000000000 1.3622588249 -1.1131922331<br>F -1.6888413563 0.3846297047 0.7247873218<br>F 1.6888413563 0.3846297047 0.7247873218
**$NOsF_3$ (^3A”)**	
B3LYP/def2-QZVP	
Cartesian coordinates	5<br>Energy = -445.0185439098<br>Os 0.1752333 -0.0877040 0.0000000<br>N 0.2353676 -1.7152545 0.0000000<br>F 0.5222079 0.4522222 1.7690544<br>F 0.5222079 0.4522222 -1.7690544<br>F -1.4550167 0.8985142 0.0000000
Vibrational data (^{14}N)	# mode symmetry wave number IR intensity selection rules<br># cm**(-1) km/mol IR RAMAN<br>7 a' 121.68 2.86186 YES YES<br>8 a" 211.12 3.47256 YES YES<br>9 a' 216.97 6.47895 YES YES<br>10 a' 238.88 5.17383 YES YES

	11 a" 310.07 2.06572 YES YES<br>12 a' 594.21 74.59639 YES YES<br>13 a' 651.66 49.64608 YES YES<br>14 a" 657.25 152.55974 YES YES<br>15 a' 1166.29 31.11246 YES YES
Vibrational data (^{15}N)	# mode symmetry wave number IR intensity selection rules<br># cm**(-1) km/mol IR RAMAN<br>7 a' 121.54 2.78571 YES YES<br>8 a" 211.11 3.45139 YES YES<br>9 a' 216.65 6.49066 YES YES<br>10 a' 234.53 5.28788 YES YES<br>11 a" 302.52 2.09521 YES YES<br>12 a' 594.21 74.58419 YES YES<br>13 a' 651.66 49.61697 YES YES<br>14 a" 657.24 152.52776 YES YES<br>15 a' 1129.68 29.51928 YES YES
BP86/def2-QZVP	
Cartesian coordinates	5<br>Energy = -445.3658941028<br>Os 0.1866027 -0.0820812 0.0000000<br>N 0.2450010 -1.7255596 0.0000000<br>F 0.5084003 0.4482530 1.7808904<br>F 0.5084003 0.4482530 -1.7808904<br>F -1.4484044 0.9111347 0.0000000
Vibrational data (^{14}N)	# mode symmetry wave number IR intensity selection rules<br># cm**(-1) km/mol IR RAMAN<br>7 a' 126.25 1.76509 YES YES<br>8 a" 209.42 2.65201 YES YES<br>9 a' 215.15 4.56824 YES YES<br>10 a' 231.58 3.37872 YES YES<br>11 a" 305.16 1.41612 YES YES

	12 a' 580.58 66.22472 YES YES<br>13 a' 636.82 36.43085 YES YES<br>14 a" 646.36 134.21937 YES YES<br>15 a' 1123.96 26.74544 YES YES
Vibrational data (^{15}N)	# mode symmetry wave number IR intensity selection rules<br># cm**(-1) km/mol IR RAMAN<br>7 a' 126.09 1.71050 YES YES<br>8 a" 209.41 2.63670 YES YES<br>9 a' 214.73 4.54806 YES YES<br>10 a' 227.52 3.49313 YES YES<br>11 a" 297.75 1.43473 YES YES<br>12 a' 580.58 66.21191 YES YES<br>13 a' 636.81 36.40334 YES YES<br>14 a" 646.35 134.19472 YES YES<br>15 a' 1088.69 25.37391 YES YES
M06-L/def2-QZVP	
Cartesian coordinates	5<br>Energy = -445.1809264953<br>Os 0.1778569 -0.0953509 0.0000000<br>N 0.2392572 -1.7201596 0.0000000<br>F 0.5167217 0.4557327 1.7693005<br>F 0.5167217 0.4557327 -1.7693005<br>F -1.4505574 0.9040451 0.0000000
Vibrational data (^{14}N)	# mode symmetry wave number IR intensity selection rules<br># cm**(-1) km/mol IR RAMAN<br>7 a' 121.82 2.74791 YES YES<br>8 a" 210.42 3.48920 YES YES<br>9 a' 217.48 5.82187 YES YES<br>10 a' 241.72 4.56685 YES YES<br>11 a" 308.95 2.18357 YES YES<br>12 a' 586.67 75.10479 YES YES

	13 a' 642.62 47.16880 YES YES<br>14 a" 649.20 149.83021 YES YES<br>15 a' 1156.15 35.21212 YES YES
CCSD(T)/aug-cc-pVTZ(-PP)	
Cartesian coordinates	5<br>UCCSD(T)/USERDEF ENERGY=-444.34259854<br>Os 0.1812594542 -0.0736561393 0.0000000000<br>N 0.2488686836 -1.7093349032 0.0000000000<br>F 0.5036103700 0.4377248947 1.7707750462<br>F 0.5036103700 0.4377248947 -1.7707750462<br>F -1.4373488777 0.9075413532 0.0000000000
Vibrational data (^{14}N)	1 A 2 A 3 A 4 A 5 A<br>Wavenumbers [cm-1] 125.96 216.50 219.56 234.60 306.28<br>Intensities [km/mol] 0.00 0.00 0.00 0.00 0.00<br>Intensities [relative] 0.00 0.00 0.00 0.00 0.00<br><br>6 A 7 A 8 A 9 A<br>Wavenumbers [cm-1] 614.29 667.96 674.85 1130.27<br>Intensities [km/mol] 0.00 0.00 0.00 0.00<br>Intensities [relative] 0.00 0.00 0.00 0.00
Vibrational data (^{15}N)	1 A 2 A 3 A 4 A 5 A<br>Wavenumbers [cm-1] 125.78 216.49 219.03 230.65 298.80<br>Intensities [km/mol] 0.00 0.00 0.00 0.00 0.00<br>Intensities [relative] 0.00 0.00 0.00 0.00 0.00<br><br>6 A 7 A 8 A 9 A<br>Wavenumbers [cm-1] 614.28 667.95 674.85 1094.66<br>Intensities [km/mol] 0.00 0.00 0.00 0.00<br>Intensities [relative] 0.00 0.00 0.00 0.00
**$NOsF_3$ (MECP)**	

B3LYP/def2-QZVP (ORCA)	
Cartesian coordinates	5<br>Coordinates from ORCA-job MECP<br>N 0.13198978036287 0.00000526875099 -0.00575459635524<br>Os 0.04917238912738 0.00001354020650 1.61364554078717<br>F 1.71184455120966 -0.00002338926068 2.50530705438433<br>F -1.20445929676235 1.28424962439923 2.24264876284551<br>F -1.20442742393756 -1.28424504409605 2.24266923833822
UHF-UCCSD(T)/cc-pVTZ(-PP) (ORCA)	
Cartesian coordinates	5<br>Coordinates from ORCA-job MECP<br>N 0.15480699413023 0.00007522168397 -0.00683259186786<br>Os 0.02655123263285 0.00016243535483 1.61155399004442<br>F 1.68159726368437 -0.00028342202728 2.49138947348428<br>F -1.18961672184577 1.29534592125077 2.25107131342794<br>F -1.18921876860169 -1.29530015626229 2.25133381491120
**$NOsF_2$ ($^2A_2$ – C_{2v})**	
B3LYP/def2-QZVP	
Cartesian coordinates	4<br>Energy = -345.1647128739<br>Os 0.0000000 0.0000000 0.0356313<br>N 0.0000000 0.0000000 -1.5815500<br>F -1.7176708 0.0000000 0.7729593<br>F 1.7176708 0.0000000 0.7729593
Vibrational data (^{14}N)	# mode symmetry wave number IR intensity selection rules<br># cm**(-1) km/mol IR RAMAN<br>7 a1 163.60 3.84836 YES YES<br>8 b2 187.24 6.72869 YES YES<br>9 b1 278.30 2.05675 YES YES<br>10 a1 654.30 47.50580 YES YES

	11 b1 674.71 138.66862 YES YES<br>12 a1 1200.12 28.52014 YES YES
Vibrational data (^{15}N)	# mode symmetry wave number IR intensity selection rules<br># cm**(-1) km/mol IR RAMAN<br>7 a1 163.55 3.84302 YES YES<br>8 b2 185.48 6.73376 YES YES<br>9 b1 271.59 2.00318 YES YES<br>10 a1 654.30 47.50381 YES YES<br>11 b1 674.68 138.59675 YES YES<br>12 a1 1162.49 26.90051 YES YES
BP86/def2-QZVP	
Cartesian coordinates	4<br>Energy = -345.4642229991<br>Os 0.0000000 0.0000000 0.0478155<br>N 0.0000000 0.0000000 -1.5830297<br>F -1.7256455 0.0000000 0.7676071<br>F 1.7256455 0.0000000 0.7676071
Vibrational data (^{14}N)	# mode symmetry wave number IR intensity selection rules<br># cm**(-1) km/mol IR RAMAN<br>7 a1 163.96 2.18989 YES YES<br>8 b2 182.37 3.90329 YES YES<br>9 b1 272.81 1.32351 YES YES<br>10 a1 649.62 37.51229 YES YES<br>11 b1 666.18 123.61120 YES YES<br>12 a1 1163.02 22.46874 YES YES
Vibrational data (^{15}N)	# mode symmetry wave number IR intensity selection rules<br># cm**(-1) km/mol IR RAMAN<br>7 a1 163.90 2.18644 YES YES<br>8 b2 180.72 3.89814 YES YES<br>9 b1 266.26 1.28252 YES YES<br>10 a1 649.62 37.50428 YES YES

	11 b1 666.17 123.56246 YES YES<br>12 a1 1126.56 21.19565 YES YES
**NOsF (^{1}A')**	
B3LYP/def2-QZVP	
Cartesian coordinates	3<br>Energy = -245.2177683460<br>Os 0.6196501 0.1325370 0.0000000<br>N -0.3723913 1.3957486 0.0000000<br>F -0.2472497 -1.5280737 0.0000000
Vibrational data (^{14}N)	# mode symmetry wave number IR intensity selection rules<br># cm**(-1) km/mol IR RAMAN<br>7 a' 216.43 0.10046 YES YES<br>8 a' 631.23 119.38082 YES YES<br>9 a' 1213.91 30.43792 YES YES
BP86/def2-QZVP	
Cartesian coordinates	3<br>Energy = -245.4741075501<br>Os 0.6327018 0.1269969 0.0000000<br>N -0.3809407 1.3900265 0.0000000<br>F -0.2517520 -1.5168115 0.0000000
Vibrational data (^{14}N)	# mode symmetry wave number IR intensity selection rules<br># cm**(-1) km/mol IR RAMAN<br>7 a' 235.06 0.65575 YES YES<br>8 a' 641.07 92.50010 YES YES<br>9 a' 1175.54 22.81104 YES YES
**$NOsF_4$ ($^2B_2$ – C_{4v})**	
B3LYP/def2-QZVP	
Cartesian coordinates	6<br>Energy = -544.9144073546

N	0.0000000	0.0000000	1.6700056
Os	0.0000000	0.0000000	0.0489409
F	1.2796581	1.2796581	-0.4297366
F	1.2796581	-1.2796581	-0.4297366
F	-1.2796581	1.2796581	-0.4297366
F	-1.2796581	-1.2796581	-0.4297366

Vibrational data (^{14}N)

# mode	symmetry	wave number	IR intensity	selection rules	
#		cm**(-1)	km/mol	IR	RAMAN
7	b2	134.10	0.00000	NO	YES
8	a1	243.58	6.63961	YES	YES
9	e	249.79	7.27783	YES	YES
10	e	249.79	7.27783	YES	YES
11	b1	286.20	0.00000	NO	YES
12	e	329.56	3.51230	YES	YES
13	e	329.56	3.51230	YES	YES
14	b2	617.24	0.00000	NO	YES
15	e	674.32	176.38993	YES	YES
16	e	674.32	176.38993	YES	YES
17	a1	686.84	30.78787	YES	YES
18	a1	1192.42	13.28492	YES	YES

Vibrational data (^{15}N)

# mode	symmetry	wave number	IR intensity	selection rules	
#		cm**(-1)	km/mol	IR	RAMAN
7	b2	134.10	0.00000	NO	YES
8	a1	243.41	6.62360	YES	YES
9	e	249.23	6.98264	YES	YES
10	e	249.23	6.98264	YES	YES
11	b1	286.20	0.00000	NO	YES
12	e	322.21	3.83251	YES	YES
13	e	322.21	3.83251	YES	YES
14	b2	617.24	0.00000	NO	YES
15	e	674.32	176.37856	YES	YES

	16 e 674.32 176.37856 YES YES<br>17 a1 686.83 30.74941 YES YES<br>18 a1 1155.11 12.72822 YES YES
BP86/def2-QZVP	
Cartesian coordinates	6<br>Energy = -545.3097556915<br>N 0.0000000 0.0000000 1.6877557<br>Os 0.0000000 0.0000000 0.0483698<br>F 1.2848923 1.2848923 -0.4340314<br>F 1.2848923 -1.2848923 -0.4340314<br>F -1.2848923 1.2848923 -0.4340314<br>F -1.2848923 -1.2848923 -0.4340314
Vibrational data (^{14}N)	# mode symmetry wave number IR intensity selection rules<br># cm**(-1) km/mol IR RAMAN<br>7 b2 132.82 0.00000 NO YES<br>8 a1 238.77 4.67881 YES YES<br>9 e 244.56 5.50861 YES YES<br>10 e 244.56 5.50861 YES YES<br>11 b1 276.83 0.00000 NO YES<br>12 e 320.81 3.08158 YES YES<br>13 e 320.81 3.08158 YES YES<br>14 b2 605.23 0.00000 NO YES<br>15 e 658.05 152.20696 YES YES<br>16 e 658.05 152.20696 YES YES<br>17 a1 664.36 24.75087 YES YES<br>18 a1 1140.46 12.94366 YES YES
Vibrational data (^{15}N)	# mode symmetry wave number IR intensity selection rules<br># cm**(-1) km/mol IR RAMAN<br>7 b2 132.82 0.00000 NO YES<br>8 a1 238.61 4.66642 YES YES<br>9 e 243.96 5.26204 YES YES

	10 e 243.96 5.26204 YES YES<br>11 b1 276.83 0.00000 NO YES<br>12 e 313.74 3.33965 YES YES<br>13 e 313.74 3.33965 YES YES<br>14 b2 605.23 0.00000 NO YES<br>15 e 658.05 152.19752 YES YES<br>16 e 658.05 152.19752 YES YES<br>17 a1 664.35 24.72035 YES YES<br>18 a1 1104.76 12.35962 YES YES
CCSD(T)/aug-cc-pVTZ(-PP)	
Cartesian coordinates	6<br>UCCSD(T)/AUG-CC-PVTZ,OS=AUG-CC-PVTZ-PP ENERGY=-544.12920372<br>N -0.0000000000 -0.0000000000 -1.6729194364<br>Os -0.0000000000 -0.0000000000 -0.0422087884<br>F 1.8021400214 0.0000000000 0.4139846943<br>F 0.0000000000 1.8021400214 0.4139846943<br>F 0.0000000000 -1.8021400214 0.4139846943<br>F -1.8021400214 0.0000000000 0.4139846943
Vibrational data (^{14}N)	1 A1 2 A1 3 B1 4 B2 5 A2<br>Wavenumbers [cm-1] 136.22 241.61 249.87 249.87 283.34<br>Intensities [km/mol] 0.00 0.00 0.00 0.00 0.00<br>Intensities [relative] 0.00 0.00 0.00 0.00 0.00<br><br>6 B2 7 B1 8 A1 9 B1 10 B2<br>Wavenumbers [cm-1] 323.23 323.23 635.02 693.20 693.20<br>Intensities [km/mol] 0.00 0.00 0.00 0.00 0.00<br>Intensities [relative] 0.00 0.00 0.00 0.00 0.00<br><br>11 A1 12 A1<br>Wavenumbers [cm-1] 705.63 1145.42<br>Intensities [km/mol] 0.00 0.00

	Intensities [relative]	0.00	0.00			
Vibrational data (^{15}N)		1 A1	2 A1	3 B1	4 B2	5 A2
	Wavenumbers [cm-1]	136.22	241.45	249.21	249.21	283.34
	Intensities [km/mol]	0.00	0.00	0.00	0.00	0.00
	Intensities [relative]	0.00	0.00	0.00	0.00	0.00
		6 B2	7 B1	8 A1	9 B1	10 B2
	Wavenumbers [cm-1]	316.15	316.15	635.02	693.20	693.20
	Intensities [km/mol]	0.00	0.00	0.00	0.00	0.00
	Intensities [relative]	0.00	0.00	0.00	0.00	0.00
		11 A1	12 A1			
	Wavenumbers [cm-1]	705.60	1109.43			
	Intensities [km/mol]	0.00	0.00			
	Intensities [relative]	0.00	0.00			

Supporting Information References

[1] T. Stüker, T. Hohmann, H. Beckers, S. Riedel, *Angew. Chem. Int. Ed.* **2020**, *59*, 23174–23179.

[2] TURBOMOLE GmbH, *TURBOMOLE V7.3*: a development of University of Karlsruhe and Forschungszentrum Karlsruhe GmbH, **2018**.

[3] a) A. D. Becke, *Phys. Rev. A* **1988**, *38*, 3098–3100; b) J. P. Perdew, *Phys. Rev. B* **1986**, *33*, 8822–8824;

[4] Y. Zhao, D. G. Truhlar, *Theor. Chem. Acc.* **2008**, *120*, 215–241.

[5] a) A. D. Becke, *J. Chem. Phys.* **1993**, *98*, 5648–5652; b) C. Lee, W. Yang, R. G. Parr, *Phys. Rev. B* **1988**, *37*, 785–789; c) P. J. Stephens, F. J. Devlin, C. F. Chabalowski, M. J. Frisch, *J. Phys. Chem.* **1994**, *98*, 11623–11627; d) S. H. Vosko, L. Wilk, M. Nusair, *Can. J. Phys.* **1980**, *58*, 1200–1211;

[6] a) F. Weigend, F. Furche, R. Ahlrichs, *J. Chem. Phys.* **2003**, *119*, 12753–12762; b) F. Weigend, R. Ahlrichs, *Phys. Chem. Chem. Phys.* **2005**, *7*, 3297–3305;

[7] D. Andrae, U. Huermann, M. Dolg, H. Stoll, H. Preu, *Theor. Chim. Acta* **1990**, *77*, 123–141.

[8] P. J. Knowles, C. Hampel, H.-J. Werner, *J. Chem. Phys.* **1993**, *99*, 5219–5227.

[9] H.-J. Werner, P. J. Knowles, G. Knizia, F. R. Manby, M. Schütz, P. Celani, W. Györffy, D. Kats, T. Korona, R. Lindh, A. Mitrushenkov, G. Rauhut, K. R. Shamasundar, T. B. Adler, R. D. Amos, S. J. Bennie, A. Bernhardsson, A. Berning, D. L. Cooper, M. J. O. Deegan, A. J. Dobbyn, F. Eckert, E. Goll, C. Hampel, A. Hesselmann, G. Hetzer, T. Hrenar, G. Jansen, C. Köppl, S. J. R. Lee,

Y. Liu, A. W. Lloyd, Q. Ma, R. A. Mata, A. J. May, S. J. McNicholas, W. Meyer, T. F. Miller III, M. E. Mura, A. Nicklass, D. P. O'Neill, P. Palmieri, D. Peng, K. Pflüger, R. Pitzer, M. Reiher, T. Shiozaki, H. Stoll, A. J. Stone, R. Tarroni, T. Thorsteinsson, M. Wang, M. Welborn, *MOLPRO, version 2019.2, a package of ab initio programs*.

[10] a) P. J. Knowles, H.-J. Werner, *Chem. Phys. Lett.* **1985**, *115*, 259–267; b) D. A. Kreplin, P. J. Knowles, H.-J. Werner, *The Journal of chemical physics* **2019**, *150*, 194106; c) H.-J. Werner, P. J. Knowles, *J. Chem. Phys.* **1985**, *82*, 5053–5063;

[11] a) C. Angeli, R. Cimiraglia, S. Evangelisti, T. Leininger, J.-P. Malrieu, *J. Chem. Phys.* **2001**, *114*, 10252–10264; b) C. Angeli, R. Cimiraglia, J.-P. Malrieu, *J. Chem. Phys.* **2002**, *117*, 9138–9153; c) C. Angeli, M. Pastore, R. Cimiraglia, *Theor Chem Acc* **2007**, *117*, 743–754;

[12] a) N. B. Balabanov, K. A. Peterson, *J. Chem. Phys.* **2005**, *123*, 64107; b) R. A. Kendall, T. H. Dunning, R. J. Harrison, *J. Chem. Phys.* **1992**, *96*, 6796–6806; c) T. H. Dunning, *J. Chem. Phys.* **1989**, *90*, 1007–1023;

[13] a) D. Figgen, K. A. Peterson, M. Dolg, H. Stoll, *J. Chem. Phys.* **2009**, *130*, 164108; b) K. A. Peterson, D. Figgen, M. Dolg, H. Stoll, *J. Chem. Phys.* **2007**, *126*, 124101;

[14] A. Wolf, M. Reiher, B. A. Hess, *J. Chem. Phys.* **2002**, *117*, 9215–9226.

[15] W. Jiang, N. J. DeYonker, A. K. Wilson, *J. Chem. Theory Comput.* **2012**, *8*, 460–468.

[16] J. Wang, S. Manivasagam, A. K. Wilson, *J. Chem. Theory Comput.* **2015**, *11*, 5865–5872.

[17] a) F. Neese, *WIREs Comput. Mol. Sci.* **2012**, *2*, 73–78; b) F. Neese, *WIREs Comput. Mol. Sci.* **2017**, *2*, e1327; c) J. N. Harvey, M. Aschi, H. Schwarz, W. Koch, *Theor Chem Acc* **1998**, *99*, 95–99;

[18] R. F. W. Bader, *Atoms in Molecules: A Quantum Theory;* Clarendon Press, **1994**.

[19] T. Lu, F. Chen, *J. Comput. Chem.* **2012**, *33*, 580–592.

[20] A. E. Reed, Schleyer, Paul v. R, *J. Am. Chem. Soc.* **1990**, *112*, 1434–1445.

[21] K. B. Wiberg, *Tetrahedron* **1968**, *24*, 1083–1096.

[22] E. D. Glendening, J. K. Badenhoop, A. E. Reed, J. E. Carpenter, J. A. Bohmann, C. M. Morales, P. Karafiloglou, C. R. Landis, F. Weinhold, *NBO 7.0;* Theoretical Chemistry Institute, University of Wisconsin, Madison, WI, **2018**.

[23] W. Humphrey, A. Dalke, K. Schulten, *Journal of Molecular Graphics* **1996**, *14*, 33-8, 27-8.

[24] X. Wang, L. Andrews, R. Lindh, V. Veryazov, B. O. Roos, *J. Phys. Chem. A* **2008**, *112*, 8030–8037.

[25] C. Vogel, F. W. Heinemann, J. Sutter, C. Anthon, K. Meyer, *Angew. Chem. Int. Ed.* **2008**, *47*, 2681–2684.

[26] T. Schlöder, T. Vent-Schmidt, S. Riedel, *Angew. Chem. Int. Ed.* **2012**, *51*, 12063–12067.

[27] A. K. Brisdon, E. G. Hope, J. H. Holloway, W. Levason, J. S. Ogden, *J. Fluorine Chem.* **1993**, *64*, 117–123.

[28] M. R. Sundberg, R. Ponec, *Inorg. Chim. Acta FIELD Full Journal Title:Inorganica Chimica Acta* **2006**, *359*, 899–906.

[29] M. Kaupp, *J. Comput. Chem.* **2007**, *28*, 320–325.

[30] B. O. Roos, A. C. Borin, L. Gagliardi, *Angew. Chem. Int. Ed.* **2007**, *46*, 1469–1472.

[31] J. J. Scepaniak, C. S. Vogel, M. M. Khusniyarov, F. W. Heinemann, K. Meyer, J. M. Smith, *Science* **2011**, *331*, 1049–1052.

[32] J. F. Berry, E. Bill, E. Bothe, S. D. George, B. Mienert, F. Neese, K. Wieghardt, *Science* **2006**, *312*, 1937–1941.

[33] a) M. E. Jacox, *J. Phys. Chem. Ref. Data* **1998**, *27*, 115–393; b) D. E. Milligan, M. E. Jacox, *J. Chem. Phys.* **1964**, *40*, 2461–2466;

[34] a) G. Sabenya, L. Lázaro, I. Gamba, V. Martin-Diaconescu, E. Andris, T. Weyhermüller, F. Neese, J. Roithova, E. Bill, J. Lloret-Fillol, M. Costas, *J. Am. Chem. Soc.* **2017**, *139*, 9168–9177; b) E. Andris, R. Navrátil, J. Jašík, G. Sabenya, M. Costas, M. Srnec, J. Roithová, *Chemistry – A European Journal* **2018**, *24*, 5078–5081;

[35] a) T. A. Betley, J. C. Peters, *J. Am. Chem. Soc.* **2004**, *126*, 6252–6254; b) J.-U. Rohde, T. A. Betley, T. A. Jackson, C. T. Saouma, J. C. Peters, Que, Lawrence, Jr, *Inorganic Chemistry (Washington, DC, United States)* **2007**, *46*, 5720–5726;

[36] L. Bucinsky, M. Breza, W.-T. Lee, A. K. Hickey, D. A. Dickie, I. Nieto, J. A. DeGayner, T. D. Harris, K. Meyer, J. Krzystek, A. Ozarowski, J. Nehrkorn, A. Schnegg, K. Holldack, R. H. Herber, J. Telser, J. M. Smith, *Inorg. Chem.* **2017**, *56*, 4752–4769.

[37] J. J. Scepaniak, M. D. Fulton, R. P. Bontchev, E. N. Duesler, M. L. Kirk, J. M. Smith, *J. Am. Chem. Soc.* **2008**, *130*, 10515–10517.

[38] M. Keilwerth, L. Grunwald, W. Mao, F. W. Heinemann, J. Sutter, E. Bill, K. Meyer, *J. Am. Chem. Soc.* **2021**, *143*, 1458–1465.

[39] W. D. Wagner, K. Nakamoto, *J. Am. Chem. Soc.* **1989**, *111*, 1590–1598.

[40] a) J. W. Buchler, C. Dreher, K. L. Lay, *Z. Naturforsch, B: Chem. Sci.* **1982**, *37*, 1155–1162; b) J. W. Buchler, C. Dreher, K.-L. Lay, A. Raap, K. Gersonde, *Inorg. Chem.* **1983**, *22*, 879–884; c) C. Campochiaro, J. A. Hofmann, D. F. Bocian, *Inorg. Chem.* **1985**, *24*, 449–450;

[41] J. T. Groves, T. Takahashi, W. M. Butler, *Inorg. Chem.* **1983**, *22*, 884–887.

[42] H.-X. Wang, L. Wu, B. Zheng, L. Du, W.-P. To, C.-H. Ko, D. L. Phillips, C.-M. Che, *Angewandte Chemie International Edition* **2020**.

[43] a) T. Petrenko, S. DeBeer George, N. Aliaga-Alcalde, E. Bill, B. Mienert, Y. Xiao, Y. Guo, W. Sturhahn, S. P. Cramer, K. Wieghardt, F. Neese, *J. Am. Chem. Soc.* **2007**, *129*, 11053–11060; b) N. Aliaga-Alcalde, S. DeBeer George, B. Mienert, E. Bill, K. Wieghardt, F. Neese, *Angew. Chem. Int. Ed.* **2005**, *44*, 2908–2912;

[44] N. B. Thompson, M. T. Green, J. C. Peters, *J. Am. Chem. Soc.* **2017**, *139*, 15312–15315.

[45] a) A. Halkier, T. Helgaker, P. Jorgensen, W. Klopper, H. Koch, J. Olsen, A. K. Wilson, *Chem. Phys. Lett.* **1998**, *286*, 243–252; b) T. Helgaker, W. Klopper, H. Koch, J. Noga, *J. Chem. Phys.* **1997**, *106*, 9639–9646;

[46] A. Halkier, T. Helgaker, P. Jørgensen, W. Klopper, J. Olsen, *Chem. Phys. Lett.* **1999**, *302*, 437–446.
